# Pnictogen-bonding catalysis: brevetoxin-type polyether cyclizations†
†Electronic supplementary information (ESI) available: Detailed procedures and results for all reported experiments. CCDC 1999316–1999326. For ESI and crystallographic data in CIF or other electronic format see DOI: 10.1039/d0sc02551h


**DOI:** 10.1039/d0sc02551h

**Published:** 2020-06-25

**Authors:** Andrea Gini, Miguel Paraja, Bartomeu Galmés, Celine Besnard, Amalia I. Poblador-Bahamonde, Naomi Sakai, Antonio Frontera, Stefan Matile

**Affiliations:** a Department of Organic Chemistry , University of Geneva , Geneva , Switzerland . Email: stefan.matile@unige.ch ; http://www.unige.ch/sciences/chiorg/matile/ ; Tel: +41 22 379 6523; b Department de Química , Universitat de les Illes Balears , Palma de Mallorca , Spain

## Abstract

Pnictogen-bond donors are attractive for use in catalysis because of deep σ holes, high multivalency, rich hypervalency, and chiral binding pockets. We here report natural product inspired epoxide-opening polyether cyclizations catalyzed by fluoroarylated Sb(v) > Sb(iii) > Bi > Sn > Ge. The distinctive characteristic found for pnictogen-bonding catalysis is the breaking of the Baldwin rules, that is selective *endo* cyclization into the *trans*-fused ladder oligomers known from the brevetoxins. Moreover, tris(3,4,5-trifluorophenyl)stibines and their hypervalent stiborane catecholates afford different *anti*-Baldwin stereoselectivity. Lewis (SbCl_3_), Brønsted (AcOH) and π acids fail to provide similar access to these forbidden rings. Like hydrogen-bonding catalysis differs from Brønsted acid catalysis, pnictogen-bonding catalysis thus emerges as the supramolecular counterpart of covalent Lewis acid catalysis.

## Abstract 2

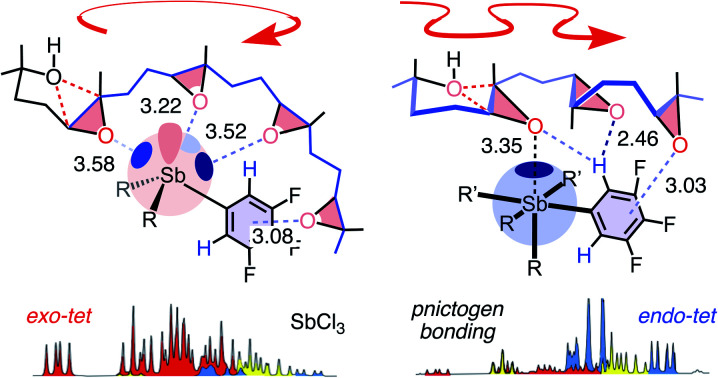
This study marks chemical space available for pnictogen-bonding catalysis, and demonstrates that reactivity accessible in this space is unique.

## 

Pnictogen and tetrel bonds refer to non-covalent interactions^1^–^7^ between electron-rich acceptors and σ holes on group V (15) and group IV (14) atoms, respectively (Fig. 1a–c).^3^,^4^ σ Holes are regions with positive electrostatic potential appearing at the side opposite to σ bonds to electron-withdrawing substituents R. Compared to better established halogen^5^ and chalcogen bonds,^6^ pnictogen- and, although less important in this study, also tetrel-bond donors are of higher valency and thus offer more σ holes. Moreover, pnictogen-bond donors can be interconversion-free^8^ stereogenic centers^9^ and at the origin of chiral axes.^10^ σ-Hole interactions are primarily electrostatic. They strengthen with the depth of the σ hole, which relates to polarizability, thus increases downward and toward the left in the periodic table.^1^,^2^

**Fig. 1:**
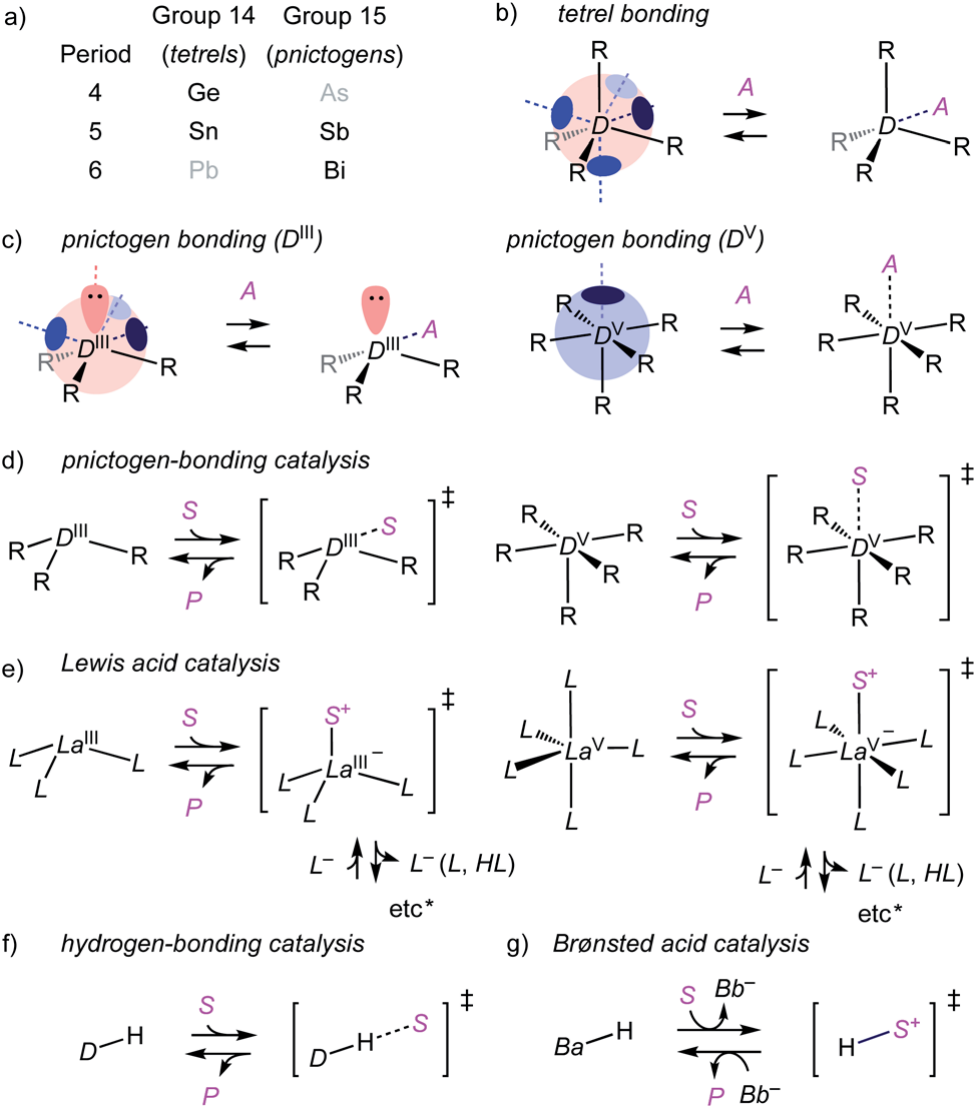
(a) Region of interest in the periodic table. (b and c) General structure of tetrel and pnictogen-bond donors (D; D^III^: trivalent; D^V^: pentavalent) interacting with their acceptors (A); blue circles, σ holes; red orbitals, lone pairs. (d) Pnictogen-bonding catalysis defined as a non-covalent counterpart of (e) Lewis acid catalysis (La), analogous to (f) hydrogen-bonding and (g) Brønsted acid catalysis (Ba, conjugate base: Bb^–^). S, substrate; P, product; etc.*: ligand (L) exchange, proton release from S upon addition to La, *etc.*

Here, we suggest to define pnictogen-bonding catalysis as the non-covalent, supramolecular counterpart of classical covalent Lewis acid catalysis (Fig. 1d and e). This is analogous to hydrogen-bonding and Brønsted acid catalysis, with interactions that become too strong transfer their proton and form new covalent bonds (Fig. 1f and g). Similarly overachieving cation-π and anion-π interactions can continue into electrophilic and nucleophilic aromatic substitution, respectively.^11^ Group 15 Lewis acids, however, have been studied exhaustively as reagents and catalysts.^2^,^12^–^14^ Except for a few recent examples,^2^,^12^,^13^ possible contributions from pnictogen bonds to these activities were either ignored or alluded to from different points of view.^14^ The question thus arises whether or not pnictogen-bonding catalysis is just a weak form of Lewis acid catalysis and thus essentially trivial. The differences between hydrogen-bonding and Brønsted acid catalysis are understood. The differences in structure and charge distribution between non-covalent pnictogen bonding and covalent ligand addition/exchange (Fig. 1d and e) further support that pnictogen-bonding catalysis should exist and matter. In the following, we show that this is indeed the case.

Most catalyst candidates **1–13** were readily accessible in a few steps from commercially available substrates (Fig. 2a, Schemes S1–S3,† X-ray structures: Fig. 2b, S75–S86†).^2^ Only Bi **7** was too unstable in our hands.^15^ Stibine **1** was obtained by nucleophilic substitution of SbCl_3_ with aryl anions derived from bromobenzene **14** (Fig. 2a). Sb(iii) **1** was oxidized with chloranil (Ch) **15**^12^ to give stiborane **2**. Molecular electrostatic potential surfaces (MEP, BP86-D3/def2-TZVP level) confirmed^12^ that this oxidation converts the three deep σ holes on Sb(iii) **1** into one deep σ hole on Sb(v) **2** (Fig. 2b and c). Consistent with increasing polarizability,^1^,^2^ Sn(iv) **3** excelled with four deep σ holes, whereas the σ holes of the smaller Ge(iv) **4** were not accessible. In **1–4**, the *ortho* fluorines of the original perfluorinated **5**^2^ were replaced by hydrogens because the crystal structure of **5** indicated the existence of Sb–F pnictogen bonds that weaken and obstruct all σ holes (Fig. 2b). The acidic *ortho* hydrogens in **1–4** should further assist σ-hole interactions with proximal C–H···A bonds (see below).

**Fig. 2:**
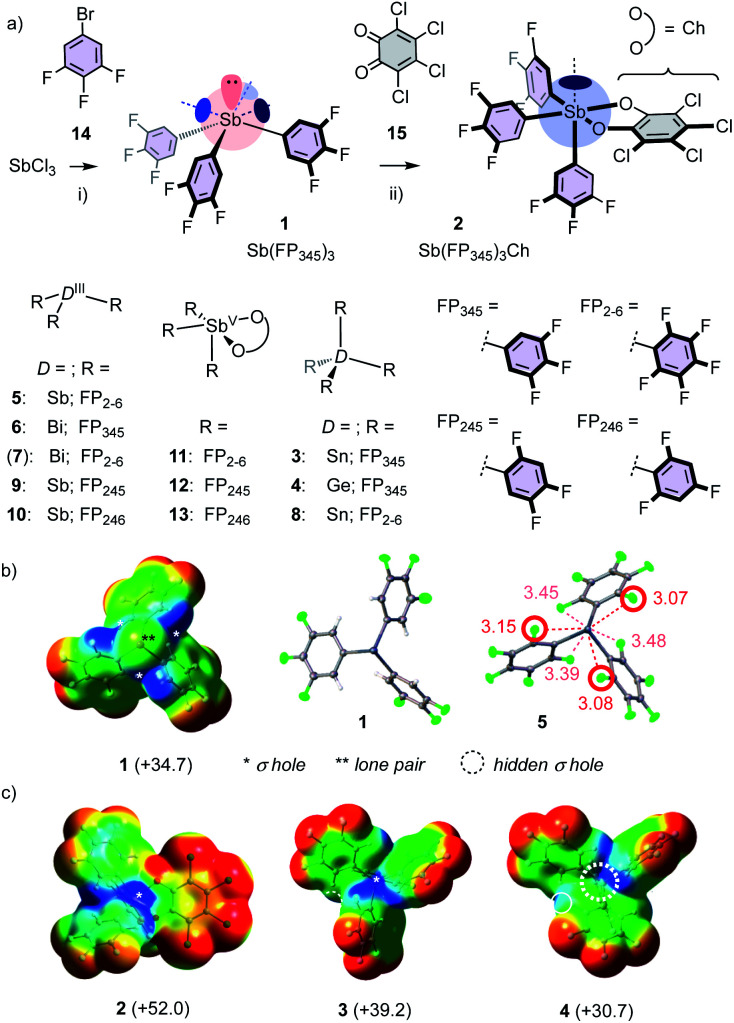
Catalyst synthesis and structures (a), with MEP and crystal structures (b and c); distances in Å; *: positive MEP maxima (kcal mol^–1^), corresponding to σ holes. Dashed circle: inaccessible σ hole. (i) *n*BuLi, Et_2_O, –78 °C to rt, 12 h, 45%; (ii) CH_2_Cl_2_, rt, 10 min, 78%.

The structural complexity of epoxide-opening ether cyclizations^16^–^18^ was considered as ideal to identify possible differences between pnictogen-bonding and Lewis acid catalysis. Initial studies focused on monomers **16–19** (Fig. 3a). According to the Baldwin (B) rules, their 5-*exo-tet* cyclization into oxolanes **20–23** is preferred over 6-*endo-tet anti*-Baldwin (A) oxanes **24–27**.^16^–^18^ After one day under standard conditions, Sb(FP_345_)_3_**1** converted 81% of *cis* epoxide **17**^17^,^18^ into (B)-**21** (Table 1, entry 1). Reactions were much slower with Bi **6**, Sn **3** and Ge **4** (entries 2–5). However, tetrel-bonding Sn **3** remained operational as catalyst, as confirmed with high conversion at 20 mol% (entry 4). FP_2-6_**5** and **8** were unstable, supporting that the *ortho* hydrogens in FP_345_ minimize not only σ-hole obstruction but also catalyst decomposition.

**Fig. 3:**
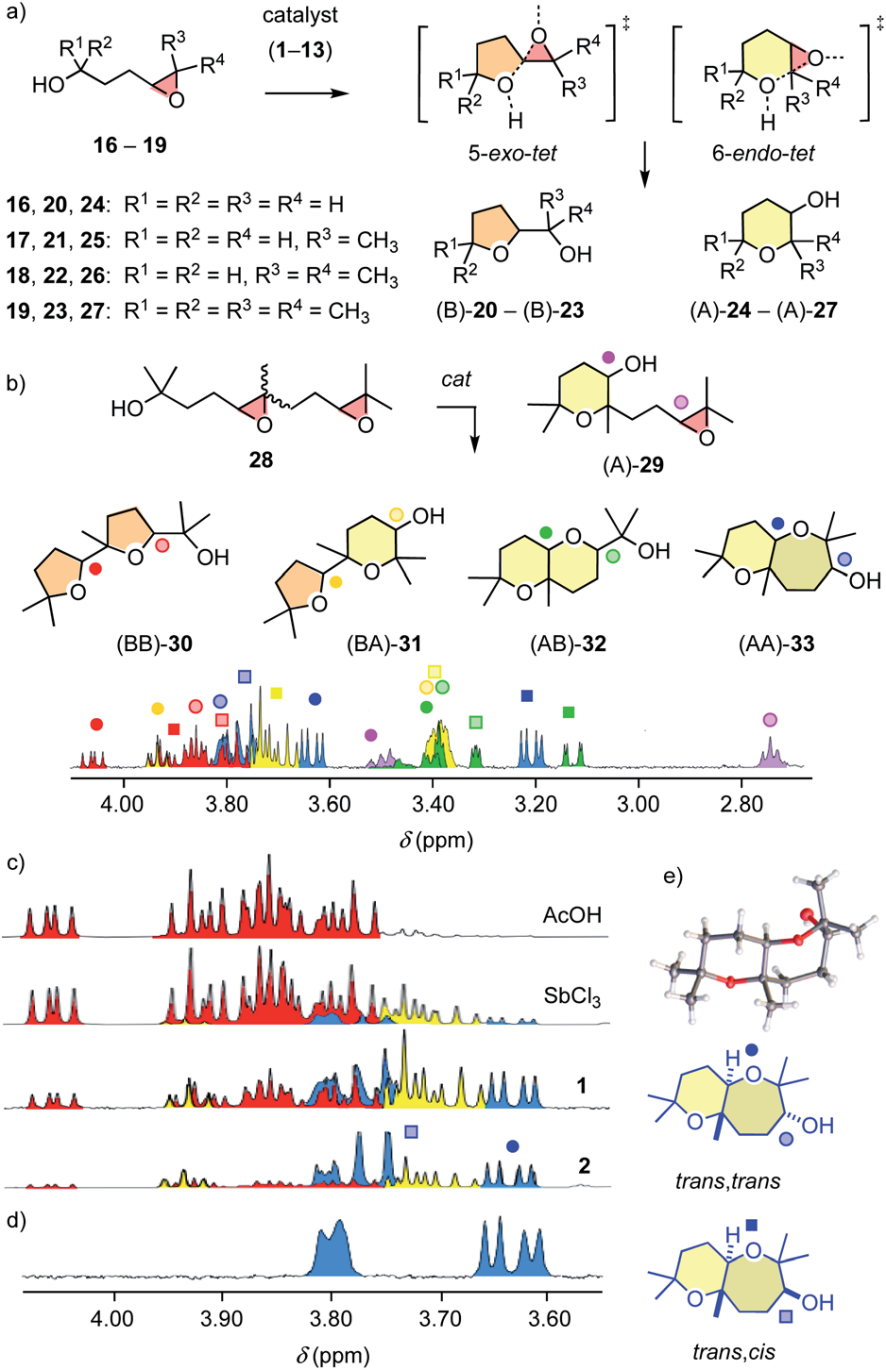
Cyclization of (a) monomers **16–19** and (b) dimers **28** with representative ^1^H NMR spectrum of a product mixture generated for **28** with **1**. (c) NMR fingerprints for **28** cyclized with AcOH, SbCl_3_, **1** and **2**. (d) ^1^H NMR spectrum and (e) crystal structure of *trans*,*trans* (AA)-**33**.

**Table 1:** Selected pnictogen- and tetrel-bonding catalysts^*a*^

	C^*b*^	*c*_C_ ^*c*^ (mol%)	S^*d*^	*c*_S_ ^*e*^ (M)	*T* ^*f*^ (°C)	*t* ^*g*^ (d)	*η*_t_ ^*h*^ (%)	B/A^*i*^
1	**1**	100	**17**	1.6	40	1	81	100 : 0
2	**6**	100	**17**	1.6	40	8	89	100 : 0
3	**3**	100	**17**	1.6	40	8	63	100 : 0
4	**3**	20	**17**	1.6	40	14	40	100 : 0
5	**4**	100	**17**	1.6	40	8	28	100 : 0
6	**1**	100	**19**	2.4	40	1	81	56 : 44
7	**1**	20	**19**	2.4	rt	9	71	59 : 41
8	**2**	10	**19**	1.0	rt	*vf*	>99	46 : 54
9	**2**	0.1	**19**	1.0	rt	*vf*	>99	25 : 75
10	**11**	1	**19**	1.0	rt	*vf*	>99	68 : 32
11	SbCl_3_	1	**19**	1.0	rt	*vf*	>99	80 : 20
12	AcOH	100	**19**	2.1	40	1	>99	92 : 8

^*a*^For more data, see Tables S1–S11.

^*b*^Catalysts.

^*c*^Concentration (CD_2_Cl_2_).

^*d*^Substrates.

^*e*^Concentration.

^*f*^Reaction temperature.

^*g*^Time to reach the given.

^*h*^Conversion; *vf*, very fast, <10 min.

^*i*^Selectivity, B = Baldwin, A = *anti*-Baldwin products.

With the permethylated monomer **19**,^17^,^18^ stibine **1** produced significant amounts of *anti*-Baldwin product (B/A ≈ 6:4, entry 6, 7). Access to *anti*-Baldwin selectivity depended on substrate (**18** > **19** > **17**, **16**) and catalyst structures (**2** > **13** > **12** > **1–9** > **11** > **10**, Tables 1, S2–S6†). Some small but significant irregularities in dependence of *endo*/*exo* selectivity on catalyst structure nicely illustrated the influence of the specific environment in the respective binding pockets with pnictogen-bonding catalysis. Proximity effects in binding pockets is a hallmark of supramolecular catalysis, much appreciated in hydrogen-bonding catalysis to access stereoselectivity, and absent in covalent “general” Brønsted acid catalysis, which is independent of the acid used. Very fast conversion on the “cyclopean” σ hole of Sb(v) stiborane **2** allowed for meaningful studies at lower temperature as well as lower catalyst loading, which caused the expected increase in *anti*-Baldwin selectivity (entry 8, 9). The *ortho*-fluorinated Gabbaï original **11**^12^ failed to break the Baldwin rules, as did Lewis and Brønsted acid controls (entries 10–12).

Access to *anti*-Baldwin cascade cyclizations was of general interest also because, in nature, Baldwin oligomers such as the monensin-like ionophores are complemented by the rich family of brevetoxin-like ladder oligomers.^16^–^18^ Minimalist cascade cyclizations were explored with a *cis–trans* mixture of diepoxide **28** to maximize the number of constitutional and stereoisomers contributing to catalyst fingerprints (Fig. 3b and c). ^1^H NMR spectroscopy and X-ray analyses of at least partially purified products and comparison with literature data^17^ allowed us to assign NMR signals to isomers **29–33** (Fig. 3b–e, S10–S13†). The *endo*/*exo* selectivity was estimated from the ratio of characteristic peaks in the spectra of the product mixtures. Isolated, easy to integrate peaks of *cis*,*cis*-(BB)-**30** and *trans*,*trans*-(AA)-**33** were selected because they originate from the same substrate isomer, *i.e.*, *trans*,*syn*-**28** (Fig. S13†). The results are described as BB/AA ratios (Table S11†).

Brønsted acid catalysis with AcOH afforded (BB)-**30** exclusively (Fig. 3c and Table S11†). With Lewis acid SbCl_3_, Baldwin selectivity persisted (BB/AA = 8 : 2). In contrast, pnictogen-bonding catalysts Sb(iii) **1** (BB/AA = 3 : 7) and Sb(v) **2** (BB/AA = 1 : 9) both broke the Baldwin rules. The Gabbaï original **11**, however, failed to do so (BB/AA = 6 : 4). The stereoselectivity of (AA)-**33** produced by Sb(iii) **1** and Sb(v) **2** differed. The according to the crystal structure (Fig. 3e and S87†) *trans*-fused *trans* epimer (AA)-**33** was reasonably accessible only with Sb(iii) **1** (*tt*/*tc* = 1 : 1), whereas the *trans*-fused *cis* epimer was the main product with the hypervalent Sb(v) **2** (*tt*/*tc* = 1 : 2; Fig. 3c, blue). The isolation of (A)-**29** as a dominant intermediate supported that the cascades are directional.

The cyclizations of trimers **34** and tetramers **35** were characterized mainly by comparing their ^1^H NMR fingerprint to those of dimers (Fig. 4, S18 and S20†). The products obtained from **34** with AcOH showed a cluster of signals between 3.75 ∼ 4.10 ppm, characteristic of Baldwin products (Fig. 4b). With pnictogen-bonding catalysts **1** and **2**, the appearance of up-field shifted peaks revealed *anti*-Baldwin selective cyclizations. NMR fingerprints of cascade cyclized tetramer **35** showed the same trends at increased complexity, containing up to 16 constitutional isomers, from (B_4_)-**36** to (A_4_)-**37** (Fig. S17†). In NMR fingerprints beyond dimers, differences between pnictogen-bonding and Lewis acid catalysis remained visible but became increasingly difficult to quantify. Gas chromatography (GC) proved more revealing, confirming the lessons learned on the dimer level: the reactivity of supramolecular pnictogen-bonding catalysts Sb(iii) **1** and Sb(v) **2** differs from covalent Lewis acid catalysts like SbCl_3_, and the former excel with an almost complete suppression of all-Baldwin products (Fig. 4c, S19 and S21†).

**Fig. 4:**
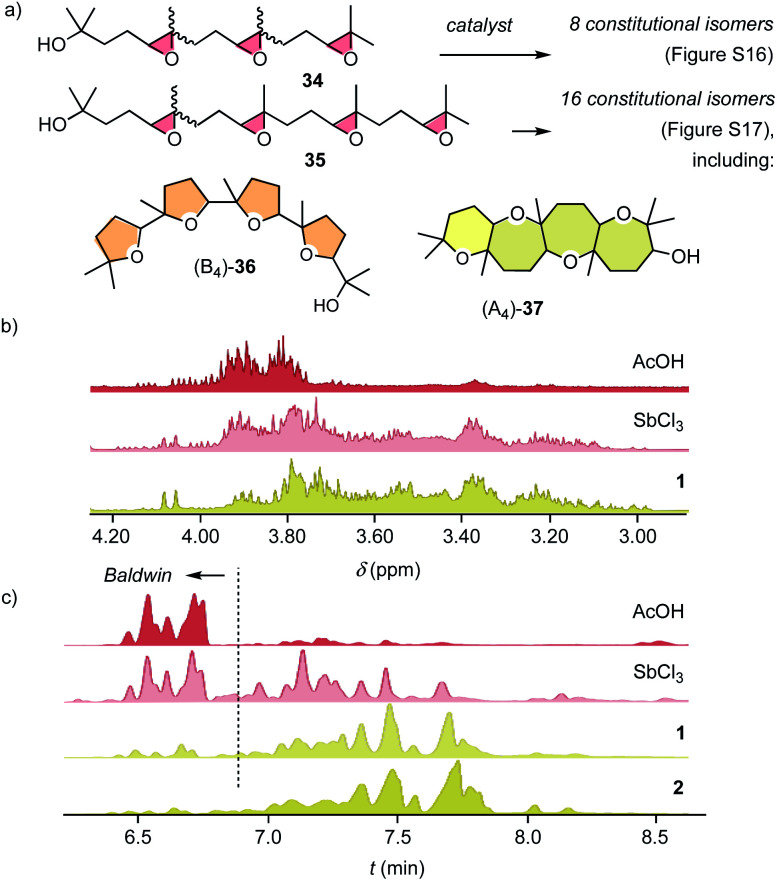
(a) Cascade cyclization of **34** and **35** with pure B and A oligomers shown as extreme products. (b) ^1^H NMR and (c) GC fingerprints of **34** converted with AcOH, SbCl_3_, **1** and **2** (c, Baldwin products: *t*_R_ 6.5–6.9 min, *anti*-Baldwin: *t*_R_ > 6.9 min, substrates: *t*_R_ < 4.0 min).

Computational studies were complicated by the high number of possible stereochemical and conformational isomers (Fig. S22–S28†). However, significant isolate observations could nevertheless be secured. Firstly, the binding of epoxide **19** to Sn **3** revealed a formal tetrel bond,^1^,^4^ shorter than the sum of vdW radii (3.69 Å) and longer than covalent bonds (2.03 Å, Fig. 5a). The smaller Ge **4** preserved the bidentate CH···O interactions but lost the tetrel bond (3.66 Å, vdW 3.62 Å). These findings were consistent with accessible σ holes on the MEP surface of Sn **3** but not Ge **4** (Fig. 2c). Although weak and presumably precedented in the Lewis acid literature,^1^,^4^,^14^ the cyclization of **17** with Sn **3** could thus be considered as one of the first examples of explicit tetrel-bonding catalysis (Table 1, entry 3, 4). Also worth noting were more than one tetrel bond with oligoepoxides (Fig. S27†), and four intermolecular tetrel bonds in the crystal structure of Sn **3** but not of Ge **4** (Fig. S77†).

**Fig. 5:**
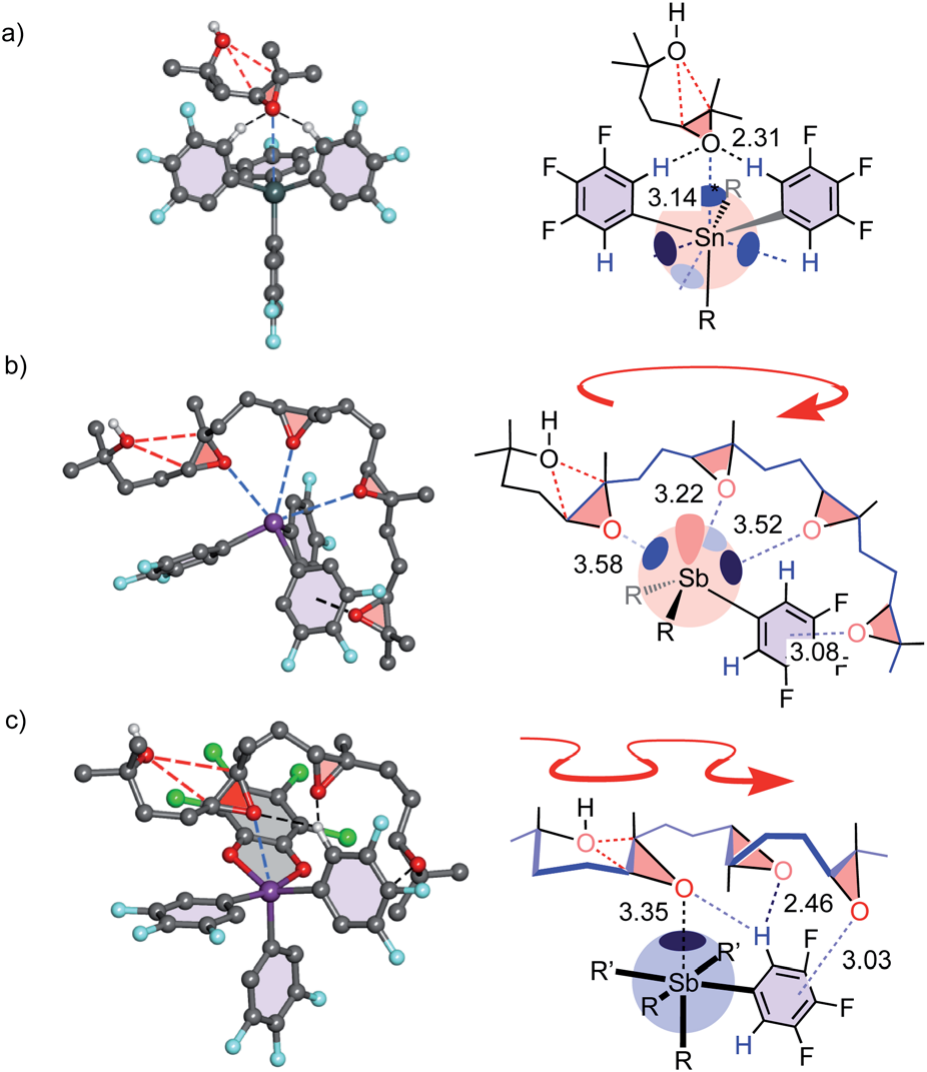
BP86-D3/def2-TZVP optimized intermediates with (a) **19** bound to **3**, (b) **35** to **1**, and (c) **34** to **2**, with schematic drawings, distances in Å, polyepoxide foldamers in (b) carousel-like with parallel and (c) snake-like conformation with antiparallel epoxides.

Most important were pnictogen bonds between epoxides of **35** to all three σ holes of **1** (Fig. 5b). This was not trivial because each pnictogen bond formed weakens the remaining σ holes.^7^ This finding thus supported contributions from multivalency, including entropy-driven substrate destabilization,^17^–^19^ to catalysis. The last epoxide is engaged in lonepair-π interactions, ready to occupy the σ hole liberated by the first ring formed. Finally, a single pnictogen bond to **34** confirmed the loss of multivalency of **2**, which was compensated by CH···O and lonepair-π interactions. Affinity gradients in the resulting “triad” would be compatible with **2** crawling along the antiparallel epoxides in snake-like foldamer tracks (Fig. 5c).

In summary, with the hypersensitive epoxide-opening polyether cascade cyclizations, we show that pnictogen-bonding catalysts are more than just weak Lewis acids. Naturally slower and not autocatalytic like on π-acidic aromatic surfaces (Fig. S6–S8†),^17^,^18^ the distinctive characteristic of pnictogen-bonding catalysis is the breaking of the Baldwin rules. Important differences in regio- and stereoselectivity exist also between multivalent Sb(iii) and hypervalent Sb(v) pnictogen-bonding catalysts. These initial results on pnictogen-bonding catalysis thus support the general expectation that the integration of unorthodox interactions^20^ will provide access to new reactivity. Attractive perspectives include antimony as stereogenic center^9^,^10^ combined with multivalency, and the integration into more advanced functional systems.^11^,^21^

The discussion about the difference between pnictogen-bonding and Lewis acid catalysis launched in this report will continue and spread into other, less affected σ-hole interactions. The need for such a distinction will have to be confirmed, and the tantalizing question where and how to draw the line will persist, particularly considering the underlying continuum and the dependence on the involved partner, either pnictogen-bond acceptor or Lewis base (*i.e.*, every weak enough pnictogen-bond acceptor will turn also a strong Lewis acid like SbCl_3_ into a pnictogen-bond donor^22^). Differences in bond length, changes in geometry, charge distribution or deprotonation (Fig. 1) are all convincing but indirect measures to draw this line; direct functional differences as identified in this study will ultimately be needed. What remains for certain is that the IUPAC definition restricts Lewis acids to reactions and covalency,^23^ while extrapolation from halogen bonding^24^ defines pnictogen-bond donors as the supramolecular counterpart, *i.e.*, electrophilic regions that interact non-covalently, rather than react covalently like Lewis acids. The comparison with non-covalent hydrogen-bonding catalysis and covalent Brønsted acids, presumably applicable to all σ-hole catalysis,^25^ could thus help in this situation because the same ambiguities exist but they are understood and appreciated. However, despite all compelling analogies, only the future will tell if σ-hole catalysis in general and pnictogen-bonding catalysis in particular will also become as important as the complementary hydrogen-bonding catalysis.

## Conflicts of interest

There are no conflicts to declare.

## Supplementary Material

Supplementary information

Crystal structure data
