# Supplementary material for: Pnictogen-bonding catalysis: brevetoxin-type polyether cyclizations
Source: Chem Sci. 2020 Jun 25;11(27):7086–91. doi: 10.1039/d0sc02551h (PMC7690316; doi:10.1039/d0sc02551h)
Supplement: Supplementary file 1 [file SC-011-D0SC02551H-s001.pdf]

Electronic Supplementary Material (ESI) for Chemical Science.
This journal is © The Royal Society of Chemistry 2020

Supplementary information

Pnictogen-bonding catalysis: Brevetoxin-type polyether cyclizations

Andrea Gini, Miguel Paraja, Bartomeu Galmés, Celine Besnard, Amalia I. Poblador-Bahamonde,

Naomi Sakai, Antonio Frontera, and Stefan Matile

Department of Organic Chemistry, University of Geneva, Geneva, Switzerland

stefan.matile@unige.ch

Table of content

1.	Materials and methods		S3
2.	Synthesis		S4
3.	Monoepoxide substrates		S13
	3.1.	Systems characterization	S13
	3.2.	Dependence on substrates	S17
	3.3.	Comparison with conventional catalysts	S23
	3.4.	Kinetics	S25
4.	Diepoxide substrates		S28
	4.1.	Systems characterization	S28
	4.2.	Product identification	S31
	4.3.	Comparison with conventional catalysts	S34
5.	Tri- and tetraepoxide oligomers		S39
	5.1.	Systems characterization	S39
	5.2.	Comparison with conventional catalysts	S45
6.	Computational studies		S49
7.	Supporting references		S80
8.	NMR spectra		S83
9.	X-ray crystallography		S110

1. Materials and methods

As in reference S1, supplementary information. Reagents for synthesis were purchased from Brunschwig, Fluka, Sigma-Aldrich, Apollo Scientific, and Acros. Column chromatography was carried out on silica gel (SiliaFlash® P60, 40-63 µm). Analytical TLC was performed on silica gel 60 (Merck, 0.2 mm). GC-FID analysis was performed on an HP 6890 series, using an HP1 column (length 30 m, ID 0.32 mm, thickness 0.25 µm). ^{1}H, ^{19}F, and ^{13}C NMR spectra were recorded (as indicated) either on a Bruker 300 MHz, 400 MHz, or 500 MHz spectrometers and are reported as chemical shifts (δ) in ppm relative to TMS (δ = 0). Spin multiplicities are reported as a singlet (s), doublet (d), triplet (t) and quartet (q), with coupling constants (J) given in Hz, or multiplet (m). Broad peaks are marked as br. ^{1}H and ^{13}C resonances were assigned with the aid of additional information from 1D and 2D NMR spectra (H,H-NOESY, H,H-COSY, DEPT 135, HSQC and HMBC).

Abbreviations. ax: Axial; DIPEA: *N,N*-Diisopropylethylamine; eq: Equatorial; GC-FID; Gas chromatography flame ionization detector; LA: Lewis acid; mCPBA: *meta*-Chloroperoxybenzoic acid; *n*-BuLi: *n*-Butyl lithium; NMR: Nuclear magnetic resonance; rt: Room temperature; THF: Tetrahydrofuran.

2. Synthesis

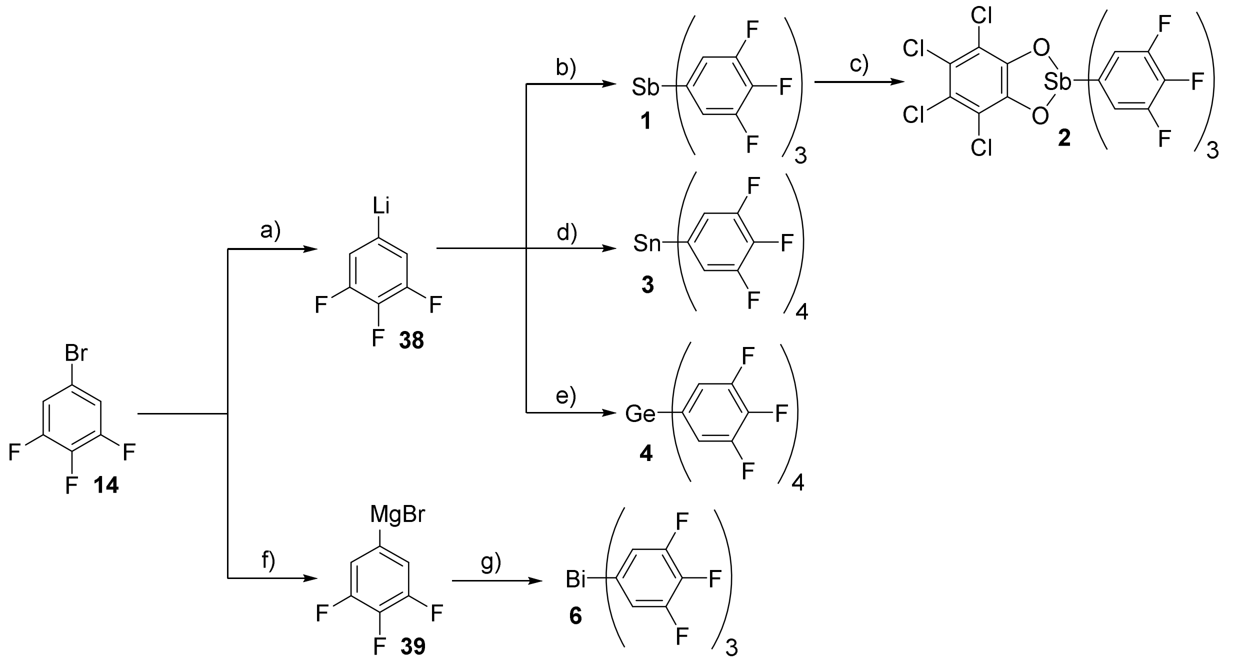

Scheme S1 (a) *n*-BuLi 1.6 M in pentane, Et_2O, -78 °C, 2 h, quantitative; (b) $SbCl_3$, diethyl ether, from -78 °C (2 h) to rt (overnight), 45%; (c) *o*-chloranil (**15**), CH_2Cl_2, rt, 10 min, 78%; (d) $SnCl_4$, diethyl ether, - 78 °C, 4 h, 43%; (e) $GeCl_4$, diethyl ether, from -78 °C (2 h) to rt (overnight), 22%; (f) Mg, THF, 0 °C to rt, 2 h, quantitative; (g) $BiCl_3$, THF, 0 °C to rt, 2 h, 48%.

Compound 38 was synthesized and utilized *in-situ* following the procedure reported in S2.

Compound 39 was synthesized and utilized *in-situ* following the procedure reported in S3.

Compound 1. To a suspension of *in-situ* generated **38** (6.62 g, 48.0 mmol) in dry Et_2O (50 mL) and pentane (30 mL) at -78 °C under nitrogen atmosphere was added dropwise a solution of $SbCl_3$ (3.54 g, 15.4 mmol) in dry Et_2O (20 mL). The solution was stirred for 2 h at -78 °C, gently warmed up to rt and stirred overnight. The reaction mixture was filtered through a pad of silica gel and the filtrate was concentrated under reduced pressure. The residue was recrystallized with pentane/CH_2Cl_2 to give the pure **1** as colorless crystals (3.64 g, 45%). The structure was proven by x-ray crystallography. Compound **1** could be reused several times without losing the activity after

recrystallization in pentane/CH_2Cl_2. ^1H NMR (500 MHz, $CDCl_3$): 6.98 (t, $^3J_{H\text{-}F}$ = $^4J_{H\text{-}F}$ = 6.3 Hz, 6H); ^{19}F NMR (282 MHz, $CDCl_3$): -131.9 (d, $^3J_{F\text{-}F}$ = 19.9 Hz, 6F), -156.9 (t, $^3J_{F\text{-}F}$ = 19.9 Hz, 3F); ^{13}C NMR (126 MHz, $CDCl_3$): 152.1 (CF, ddd, $^1J_{C\text{-}F}$ = 257.5 Hz, $^2J_{C\text{-}F}$ = 10.0 Hz, $^3J_{C\text{-}F}$ =2.3 Hz), 140.9 (CF, dt, $^1J_{C\text{-}F}$ = 256.2 Hz, $^2J_{C\text{-}F}$ =15.0 Hz), 132.7 – 130.6 (CSb, m), 119.5 (CH, dd, $^2J_{C\text{-}F}$ =14.9 Hz, $^3J_{C\text{-}F}$ = 5.0 Hz).

Compound 2. To a solution of **1** (1.03 g, 2.00 mmol) in CH_2Cl_2 (20 mL) at rt was added *o*-chloranil (0.492 g, 2.00 mmol). The formation of a yellow precipitate was immediately observed. After 10 min, the reaction mixture was diluted with pentane and cooled to 0 °C to promote the precipitation of **2**. The solid was collected by filtration and washed with pentane to give pure **2** as a yellow solid (1.19 g, 78%). Further recrystallization with pentane/CH_2Cl_2 gave **2** as yellow crystals.[S4] The structure was proven by x-ray crystallography. ^1H NMR (500 MHz, $CDCl_3$): 7.41 (t, $^3J_{H\text{-}F}$ = $^4J_{H\text{-}F}$ = 6.0 Hz, 6H); ^{19}F NMR (282 MHz, $CDCl_3$): -128.2 (d, $^3J_{F\text{-}F}$ = 19.4 Hz, 6F), -150.4 (t, $^3J_{F\text{-}F}$ = 19.4 Hz, 3F); ^{13}C NMR (126 MHz, $CDCl_3$): 152.4 (CF, dd, $^1J_{C\text{-}F}$ = 260.2 Hz, $^2J_{C\text{-}F}$ = 10.1 Hz), 143.2 (CF, dt, $^1J_{C\text{-}F}$ = 262.2 Hz, $^2J_{C\text{-}F}$ = 14.9 Hz), 142.9 (CO), 129.4 – 129.1 (CSb, m), 122.5 (CCl), 119.5 (CH, dd, $^2J_{C\text{-}F}$ = 15.7 Hz, $^3J_{C\text{-}F}$ = 5.6 Hz), 117.6 (CCl).

Compound 3. Dry $SnCl_4$ (0.65 g, 2.5 mmol) was added slowly to dry Et_2O (5 mL) at -78 °C (exothermic process) under strong agitation and nitrogen atmosphere. The suspension was gently warmed to allow a better stirring and then re-cooled down rapidly at -78 °C. An *in-situ* prepared suspension of **38** (1.38 g. 10.0 mmol) in dry Et_2O (10 mL) and pentane (6.3 mL) at -78 °C was gently warmed until a clear solution was obtained (WARNING: At rt **38** tends to polymerize in a strongly exothermic process) and then rapidly added to the suspension of $SnCl_4$. After 4 h, the obtained suspension was warmed up to rt, the reaction mixture was filtered through a pad of silica gel and the filtrate was concentrated under reduced pressure. The residue was recrystallized using pentane/CH_2Cl_2 to give pure **3** as colorless crystals (0.69 g, 43%). The structure

was proven by x-ray crystallography. ^{1}H NMR (300 MHz, $CDCl_3$): 7.22 – 6.86 (m, 8H); ^{19}F NMR (282 MHz, $CDCl_3$): -131.3 – -131.8 (m, 8F), -155.6 – -156.1 (m, 4F); ^{13}C NMR (126 MHz, $CDCl_3$): 154.1 – 150.7 (CF, m), 141.7 (CF, dt, $^{1}J_{C\text{-}F}$ = 257.5 Hz, $^{2}J_{C\text{-}F}$ = 14.9 Hz), 132.3 – 126.8 (CSn, m), 120.7 – 119.9 (CH, m).

Compound 4. To a suspension of *in-situ* generated **38** (1.38 g, 10.0 mmol) in dry Et_2O (10 mL) and pentane (6.3 mL) at -78 °C was added dropwise a solution of $GeCl_4$ (0.54 g, 2.5 mmol) in dry Et_2O (5 mL). The solution was stirred for 2 h at -78 °C and then gently warmed up to rt and stirred overnight. The reaction mixture was filtered through a pad of silica gel and the filtrate was concentrated under reduced pressure. Silica gel column chromatography of the residue (pentane, R_f: 0.53) followed by recrystallization in pentane gave pure **4** as colorless crystals (0.33 g, 22%). The structure was proven by x-ray crystallography. ^{1}H NMR (300 MHz, $CDCl_3$): 7.00 (t, $^{3}J_{H\text{-}F}$ = $^{4}J_{H\text{-}F}$ = 6.3 Hz, 8H); ^{19}F NMR (282 MHz, $CDCl_3$): -131.2 (d, $^{3}J_{F\text{-}F}$ = 20.0 Hz, 8F), -155.4 (t, $^{3}J_{F\text{-}F}$ = 20.0 Hz, 4F); ^{13}C NMR (101 MHz, $CDCl_3$): 152.2 (CF, ddd, $^{1}J_{C\text{-}F}$ = 257.6 Hz, $^{2}J_{C\text{-}F}$ = 10.0 Hz, $^{3}J_{C\text{-}F}$ = 3.1 Hz), 141.7 (CF, dt, $^{1}J_{C\text{-}F}$ = 257.9 Hz, $^{2}J_{C\text{-}F}$ = 15.2 Hz), 128.0 (CSb, q, $^{3}J_{C\text{-}F}$ = 4.1 Hz), 118.8 (CH, dd, $^{2}J_{C\text{-}F}$ = 14.1 Hz, $^{3}J_{C\text{-}F}$ = 5.7 Hz).

Compound 6. To a suspension of *in-situ* generated **39** (2.26 g, 9.6 mmol) in dry THF (10 mL) at 0 °C under nitrogen atmosphere was added dropwise a solution of $BiCl_3$ (0.95 g, 3.0 mmol) in dry THF (10 mL). Then, the mixture was stirred for 2 h at rt. The reaction mixture was cooled down to 0 °C to facilitate the precipitation of the various salts and then filtered through a pad of silica gel. The filtrate was concentrated under reduced pressure and the residue was triturated several times with pentane to give pure **6** as a pale-yellow solid (0.88 g, 48%). Further recrystallization in pentane/CH_2Cl_2 gave **6** as colorless crystals. The structure was proven by x-ray crystallography. ^{1}H NMR (300 MHz, $CDCl_3$): 7.37 – 7.26 (m, 6H); ^{19}F NMR (282 MHz, $CDCl_3$): -132.2 (d, $^{3}J_{F\text{-}F}$ = 19.6 Hz, 6F), -158.1 (t, $^{3}J_{F\text{-}F}$ = 19.6 Hz, 3F); ^{13}C NMR (101 MHz, $CDCl_3$): 154.5

(CF, dd, $^1J_{C\text{-}F}$ = 259.0 Hz, $^2J_{F\text{-}F}$ = 9.9 Hz), 151.5 – 149.2 (CBi, m), 140.3 (CF, dt, $^1J_{C\text{-}F}$ = 254.8 Hz, $^2J_{C\text{-}F}$ = 15.3 Hz), 120.9 (CH, dd, $^2J_{C\text{-}F}$ = 14.0 Hz, $^3J_{C\text{-}F}$ = 5.2 Hz).

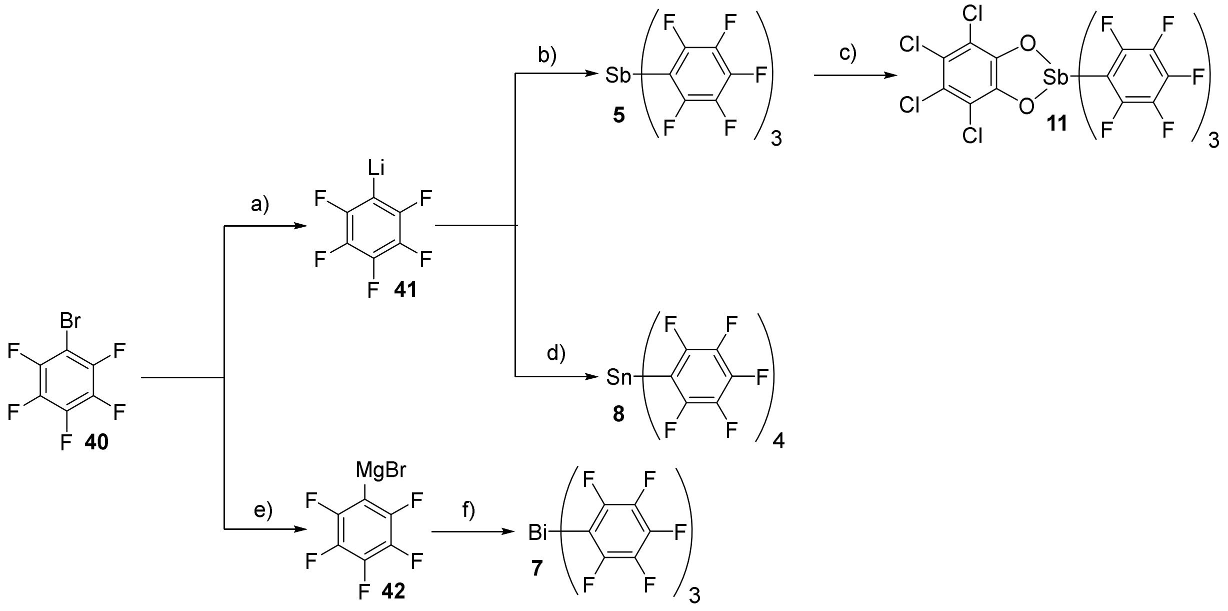

Scheme S2. (a) *n*-BuLi 1.6 M in pentane, Et_2O, -78 °C, 2 h, quantitative; (b) $SbCl_3$, diethyl ether, from -78 °C (2 h) to rt (2 h), 76%; (c) *o*-chloranil (**15**), CH_2Cl_2, rt, 10 min, 74%; (d) $SnCl_4$, diethyl ether, -78 °C, 4 h, 50%; (e) Mg, THF, 0 °C to rt, 2 h, quantitative; (f) $BiCl_3$, THF, -10 °C to rt, 1 h, decomposed.

Compound 41 was synthesized and utilized *in-situ* following the procedure reported in S2.

Compound 42 was synthesized and utilized *in-situ* following the procedure reported in S3.

Compound 5. To a suspension of *in-situ* generated **41** (6.70 g, 38.4 mmol) in dry Et_2O (20 mL) and pentane (24 mL) at -78 °C under nitrogen atmosphere was added dropwise a solution of $SbCl_3$ (2.74 g, 12.0 mmol) in dry Et_2O (15 mL). The solution was stirred for 2 h at -78 °C, gently warmed up to rt and stirred for 2 h. The reaction mixture was filtered through a pad layered with active charcoal, celite, and silica gel. The filtrate was concentrated under reduced pressure to give pure **5** as a colorless solid (5.72 g, 76%). Further recrystallization in pentane/CH_2Cl_2 gave **5** as

colorless crystals. The structure was proven by x-ray crystallography. Spectroscopic data of **5** were consistent with those reported in the literature.[S5]

Compound 11. To a solution of **5** (0.31 g, 0.60 mmol) in CH_2Cl_2 (3 mL) at rt was added *o*-chloranil (0.15 g, 0.60 mmol). The formation of a red precipitate was immediately observed. After 10 min, the reaction mixture was diluted with pentane and cooled to 0 °C to promote the precipitation of **11**. The solid was collected by filtration and washed with pentane to give pure **11** as a red/orange solid (0.39 g, 74%). Further recrystallization with pentane/CH_2Cl_2 gave **11** as orange crystals. Spectroscopic data of **11** were consistent with those reported in the literature.[S4]

Compound 8. Dry $SnCl_4$ (0.65 g, 2.5 mmol) was added slowly to dry Et_2O (5 mL) at -78 °C (exothermic process) under strong agitation and nitrogen atmosphere. The suspension was gently warmed to allow a better stirring and then re-cooled down rapidly at -78 °C. An *in-situ* prepared suspension of **41** (1.74 g. 10.0 mmol) in dry Et_2O (10 mL) and pentane (6.3 mL) at -78 °C was gently warmed until a clear solution was obtained (WARNING: At rt **41** tends to polymerize in a strongly exothermic process) and then rapidly added to the suspension of $SnCl_4$. After 4 h, the obtained suspension was warmed up to rt and the reaction mixture was filtered through a pad of silica gel. The filtrate was concentrated under reduced pressure and the residue was recrystallized using pentane/CH_2Cl_2 to give pure **8** as colorless crystals (0.98 g, 50%). Spectroscopic data of **8** were consistent with those reported in the literature. [S6]

Compound 7. To a suspension of *in-situ* generated **42** (2.3 g, 9.6 mmol) in dry THF (10 mL) at -10 °C under nitrogen atmosphere was added dropwise a solution of $BiCl_3$ (0.95 g, 3.0 mmol) in dry THF (10 mL). Next, the mixture was stirred for 1 h while gently warmed up to rt. The formation of **7** was confirmed by ^{19}F NMR analysis of the reaction mixture. However, it decomposed upon concentration to yellow unknown polymer and bismuth-based salt.

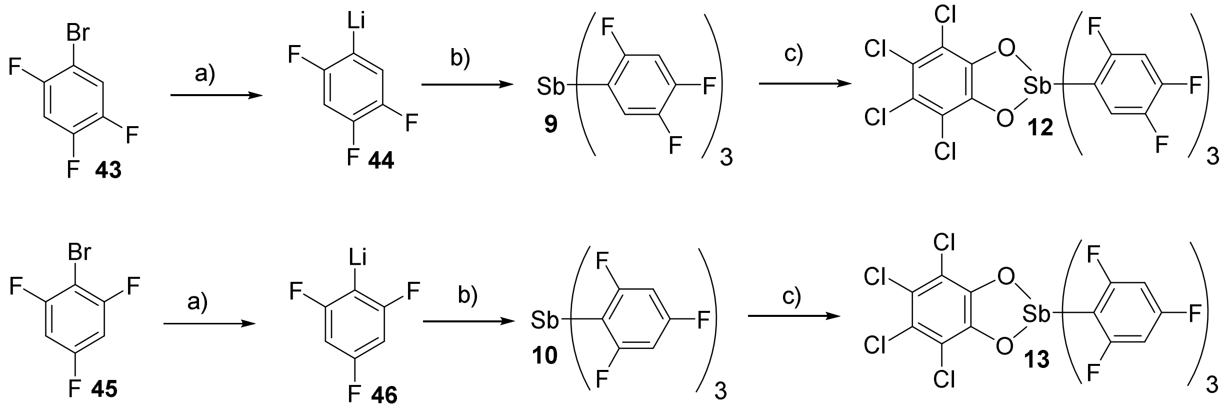

Scheme S3 (a) *n*-BuLi 1.6 M in pentane, Et_2O, -78°C, 2 h, quantitative; (b) $SbCl_3$, diethyl ether, from -78 °C (2 h) to rt (1 h) 46% (**9**) and 62% (**10**); (c) *o*-chloranil (**15**), CH_2Cl_2, rt, 10 min, 21% (**12**) and 38% (**13**).

Compound 44 was synthesized and utilized *in-situ* following the procedure reported in S2.

Compound 46 was synthesized and utilized *in-situ* following the procedure reported in S3.

Compound 9. To a suspension of *in-situ* generated **44** (1.24 g, 9.00 mmol) in dry Et_2O (5.0 mL) and pentane (5.6 mL) at -78 °C under nitrogen atmosphere was added dropwise a solution of $SbCl_3$ (0.68 g, 3.0 mmol) in dry Et_2O (3.0 mL). The solution was stirred for 2 h at -78 °C, gently warmed up to rt and stirred for 1 h. The reaction mixture was filtered through a pad of silica gel and the filtrate was concentrated under reduce pressure. The residue was washed with pentane to give pure **9** as a colorless solid (0.71 g, 46%). Further recrystallization with pentane/CH_2Cl_2 gave **9** as colorless crystals. The structure was proven by x-ray crystallography. ^{1}H NMR (500 MHz, $CDCl_3$): 7.04 (dt, $^3J_{H\text{-}F}$ = 9.9 Hz, $^4J_{H\text{-}F}$ = 6.2 Hz, 3H), 6.81 (td, $^3J_{H\text{-}F}$ = 8.9 Hz, $^4J_{H\text{-}F}$ = 4.3 Hz, 3H); ^{19}F NMR (282 MHz, $CDCl_3$): -97.9 (dd, $^4J_{F\text{-}F}$ = 15.4 Hz, $^5J_{F\text{-}F}$ = 5.2 Hz, 3F), -129.2 (dd, $^3J_{F\text{-}F}$ = 20.4 Hz, $^5J_{F\text{-}F}$ = 5.2 Hz, 3F), -140.6 (dd, $^3J_{F\text{-}F}$ = 20.4 Hz, $^4J_{F\text{-}F}$ = 15.4 Hz, 3F); ^{13}C NMR (126 MHz, $CDCl_3$): 160.8 (CF, ddd, $^1J_{C\text{-}F}$ = 237.9 Hz, $^3J_{C\text{-}F}$ = 8.9 Hz, $^4J_{C\text{-}F}$ = 2.6 Hz), 152.0 (CF, dt, $^1J_{C\text{-}F}$ = 255.6 Hz, $^2J_{C\text{-}F}$ = $^3J_{C\text{-}F}$ = 14.0 Hz), 148.4 (CF, ddd, $^1J_{C\text{-}F}$ = 250.2 Hz, $^2J_{C\text{-}F}$ = 12.4 Hz, $^3J_{C\text{-}F}$ = 4.3

Hz), 123.6 (CH, dd, $^{2}J_{C\text{-}F}$ = 18.5 Hz, $^{3}J_{C\text{-}F}$ = 12.0 Hz), 116.5 (CSb, d, $^{2}J_{C\text{-}F}$ = 36.5 Hz), 105.7 (CH, dd, $^{2}J_{C\text{-}F}$ = 32.4 Hz, $^{2}J_{C\text{-}F}$ = 20.9 Hz).

Compound 12. To a solution of **9** (206 mg, 0.400 mmol) in CH_2Cl_2 (2 mL) at rt was added *o*-chloranil (98 mg, 0.40 mmol). The formation of a green/grey precipitate was immediately observed. After 10 min, the reaction mixture was diluted with pentane and cooled to 0 °C to promote the precipitation of **12**. The solid was collected by filtration, washed with pentane and precipitated in pentane/CH_2Cl_2 to give pure **12** as an olive solid (64 mg, 21%).[S4] ^{1}H NMR (500 MHz, $CDCl_3$): 7.65 (td, $^{3}J_{H\text{-}F}$ = 9.5 Hz, $^{3}J_{H\text{-}F}$ = 3.6 Hz, 3H), 7.15 – 7.05 (m, 3H); ^{19}F NMR (282 MHz, $CDCl_3$): -97.7 – -104.2 (m, 3F), -123.7 (dd, $^{3}J_{F\text{-}F}$ = 20.4 Hz, $^{5}J_{F\text{-}F}$ = 6.7 Hz, 3F), -137.9 (dd, $^{3}J_{F\text{-}F}$ = 20.4 Hz, $^{4}J_{F\text{-}F}$ = 14.3 Hz, 3F); ^{13}C NMR (126 MHz, $CDCl_3$): 159.9 (CF, ddd, $^{1}J_{C\text{-}F}$ = 243.8 Hz, $^{3}J_{C\text{-}F}$ = 10.0 Hz, $^{4}J_{C\text{-}F}$ = 2.4 Hz), 153.7 (CF, dt, $^{1}J_{C\text{-}F}$ = 260.0 Hz, $^{2}J_{C\text{-}F}$ = $^{3}J_{C\text{-}F}$ = 13.7 Hz), 148.1 (CF, dd, $^{1}J_{C\text{-}F}$ = 251.4, $^{2}J_{C\text{-}F}$ = 13.4 Hz), 143.2 (CO), 122.5 (CH, dd, $^{2}J_{C\text{-}F}$ = 21.0 Hz, $^{3}J_{C\text{-}F}$ = 9.2 Hz), 122.4 (CCl), 120.1 (CSb, d, $^{2}J_{C\text{-}F}$ = 33.1 Hz), 117.2 (CCl), 107.1 (CH, dd, $^{2}J_{C\text{-}F}$ = 30.8 Hz, $^{2}J_{C\text{-}F}$ = 21.2 Hz).

Compound 10. To a suspension of *in-situ* generated **46** (1.24 g, 9.00 mmol) in dry Et_2O (5.0 mL) and pentane (5.6 mL) at -78 °C under nitrogen atmosphere was added dropwise a solution of $SbCl_3$ (0.68 g, 3.0 mmol) in dry Et_2O (3.0 mL). The solution was stirred for 2 h at -78 °C and then gently warmed up to rt and stirred for 1 h. The reaction mixture was filtered through silica and the solvent was removed under reduced pressure. Silica gel column chromatography of the residue (pentane) (R_f: 0.80) gave **10** as a colorless gel (0.96 g, 62%). Further recrystallization at -18 °C with pentane gave **10** as colorless crystals. The structure was proven by x-ray crystallography. ^{1}H NMR (500 MHz, $CDCl_3$): 6.65 (dd, $^{3}J_{H\text{-}F}$ = 8.8 Hz, $^{3}J_{H\text{-}F}$ = 5.9 Hz, 6H); ^{19}F NMR (282 MHz, $CDCl_3$): -90.7 (t, $^{4}J_{F\text{-}F}$ = 3.0 Hz, 3F), -90.7 (t, $^{4}J_{F\text{-}F}$ = 3.0 Hz, 3F), -105.7 – -105.8 (m, 3F); ^{13}C NMR

(126 MHz, $CDCl_3$): 166.2 (CF, dt, $^1J_{C-F}$ = 243.4 Hz, $^3J_{C-F}$ = 17.0 Hz), 165.1 (CF, dt, $^1J_{C-F}$ = 251.0 Hz, $^3J_{C-F}$ = 15.4 Hz), 104.0 (CSb, t, $^2J_{C-F}$ = 39.1 Hz), 101.0 – 99.6 (CH, m).

Compound 13. To a solution of **10** (206 mg, 0.400 mmol) in CH_2Cl_2 (20 mL) at rt was added *o*-chloranil (98 mg, 0.40 mmol). The formation of a yellow precipitate was immediately observed. After 10 min, the reaction mixture was diluted with pentane and cooled to 0 °C to favor the precipitation of **13**. The solid was collected by filtration and washed with pentane to give pure **13** as a yellow solid (117 mg, 38%). Further recrystallization with pentane/CH_2Cl_2 gave **13** as yellow crystals.[S4] The structure was proven by x-ray crystallography. ^{1}H NMR (500 MHz, $CDCl_3$): 6.80 (dd, $^3J_{H-F}$ = 8.4 Hz, $^3J_{H-F}$ = 6.1 Hz, 6H); ^{19}F NMR (282 MHz, $CDCl_3$): -94.6 – -94.8 (m, 6F), -99.2 – -99.5 (m, 3F); ^{13}C NMR (126 MHz, $CDCl_3$): 167.5 – 165.1 (CF, m), 164.2 (CF, dt, $^1J_{C-F}$ = 249.1 Hz, $^3J_{C-F}$ = 14.8 Hz), 143.53 (CO), 122.0 (CCl), 116.9 (CCl), 112.1 (CSb, t, $^2J_{C-F}$ = 35.3 Hz), 101.8 (CH, dd, $^2J_{C-F}$ = 30.0 Hz, $^2J_{C-F}$ = 25.3 Hz).

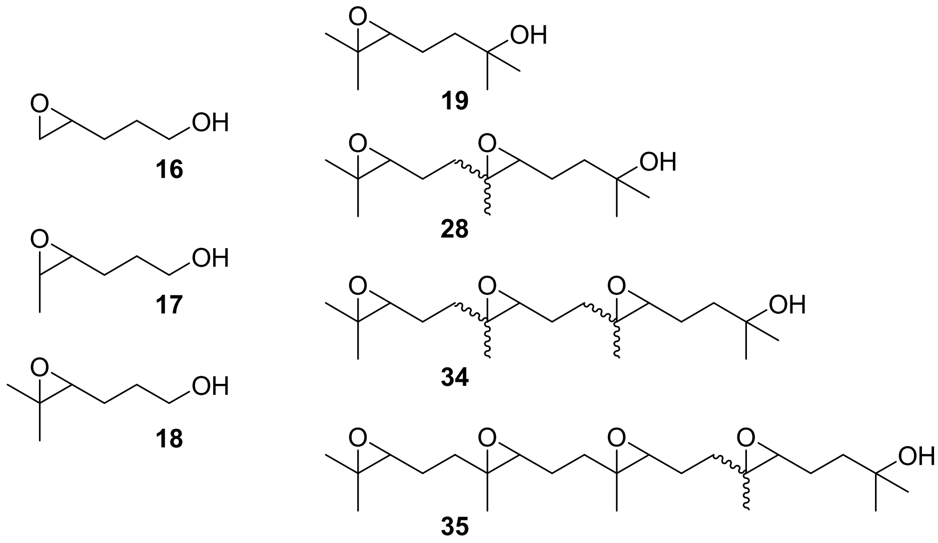

Scheme S4 Substrate structures used in this paper. Compounds **16**, **17**, and **18** were synthesized following the general procedure in ref. S7. Compound **19** was synthesized following the general procedure in ref. S8. Compound **28** was synthesized following the general procedure in ref. S8, using an *E*/*Z* isomeric mixture of 6,10-dimethyl-5,9-undecadien-2-one (*E*/*Z* ratio: 6:4).[S9] Compound **34** was synthesized following the general procedure in ref. S8 using an *E*/*Z* isomeric mixture of farnesylacetone (a mixture of isomers, major 5*E*,9*E*).[S9] Compound **35** was synthesized following the general procedure in ref. S8 using an *E*/*Z* isomeric mixture of teprenone (a mixture of 5*E*,9*E*,13*E* isomer and 5*Z*,9*E*,13*E* isomer).

3. Monoepoxide substrates

3.1. Systems characterization

General procedure. To a solution of the corresponding epoxide in CD_2Cl_2 was added the corresponding catalyst, then the mixture was heated at the corresponding temperature. The consumption of the starting material was followed by ^1H NMR spectroscopy. The conversion was calculated from the ^1H NMR spectrum of the reaction mixture by comparing the integrals of the signals assigned to the substrate and the catalyst (used also as an internal standard). The starting material was converted only in the Baldwin and anti-Baldwin cyclic ethers. When the catalyst signal was not detectable, CH_2Br_2 was added as an internal standard (0.3 M).

Table S1 Preliminary catalyst screening with **19** as a substrate[a]

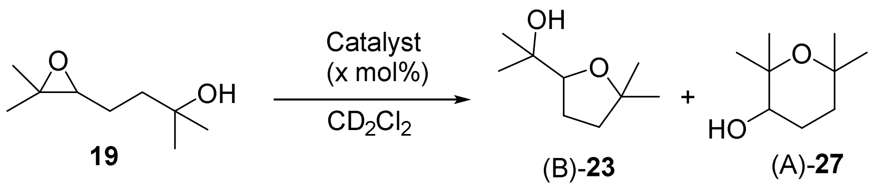

Entry		C (mol%)[b]	η_t (%)[c]	B/A[d]
1	-	-	13	100:0
2	**1**	$Sb(FP_{345})_3$ (20)	83	66:34
3	**9**	$Sb(FP_{245})_3$ (20)	68	68:32
4	**10**	$Sb(FP_{246})_3$ (20)	38	100:0

[a]The general procedure for systems characterization was used, **19** as a substrate (0.50 M), rt, CD_2Cl_2 as a solvent, 30 d. [b]Catalysts, FP = fluorophenyls, numbers indicate position of fluorines. In bracket, catalyst concentration in mol%. [c]Substrate conversion determined by ^1H NMR spectroscopy. [d]Selectivity, B = Baldwin, A = anti-Baldwin products. Estimated error: 6%.

Table S2 Conditions screening with **1** as a catalyst and **19** as a substrate[a]

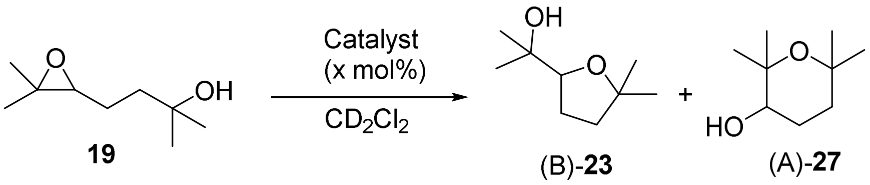

19 (B)-**23** (A)-**27**

Entry		C (mol%)[b]	c (M)[c]	T (°C)[d]	t (d)[e]	η_t (%)[f]	B/A[g]
1	**1**	$Sb(FP_{345})_3$ (20)	0.9	rt	9	54	64:36
2	**1**	$Sb(FP_{345})_3$ (20)	1.0	rt	9	58	61:39
3	**1**	$Sb(FP_{345})_3$ (20)	2.4	rt	9	71	59:41
4	**1**	$Sb(FP_{345})_3$ (50)	2.4	rt	5	73	62:38
5	**1**	$Sb(FP_{345})_3$ (100)	2.4	rt	5	80	61:39
6	**1**	$Sb(FP_{345})_3$ (200)	1.5	rt	5	82	61:39
7	**1**	$Sb(FP_{345})_3$ (500)	0.5	rt	4	86	57:43
8	**1**	$Sb(FP_{345})_3$ (100)	2.4	40	1	81	56:44

[a]The general procedure for systems characterization was used, **19** as a substrate, CD_2Cl_2 as a solvent. [b]Catalysts, FP = fluorophenyls, numbers indicate the position of fluorines. In bracket, catalyst concentration in mol%. [c]Substrate concentration. [d]Reaction temperature. [e]Reaction time to reach the indicated conversion. [f]Substrate conversion determined by ^{1}H NMR spectroscopy. [g]Selectivity, B = Baldwin, A = anti-Baldwin products. Estimated error: 6%.

Table S3 New catalyst screening with **19** as a substrate[a]

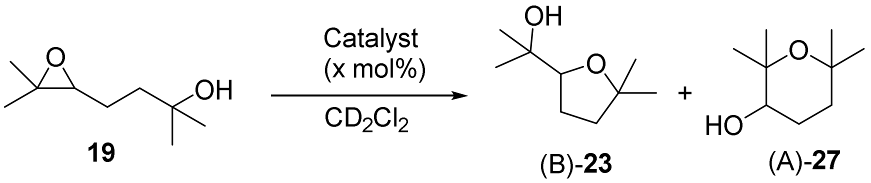

Entry		C (mol%)[b]	*c* (M)[c]	*T* (°C)[d]	*t* (d)[e]	η_t (%)[f]	B/A[g]
1	**1**	$Sb(FP_{345})_3$ (100)	2.4	40	1	81	56:44
2[h]	**5**	$Sb(FP_{2\text{-}6})_3$ (100)	2.4	40	-	-	-
3	**9**	$Sb(FP_{245})_3$ (100)	2.4	40	1	79	57:43
4	**10**	$Sb(FP_{246})_3$ (100)	2.4	40	5	33	89:11
5	**6**	$Bi(FP_{345})_3$ (100)	2.4	40	4	91	81:19
6	**3**	$Sn(FP_{345})_4$ (100)	2.4	40	4	61	83:17
7	**2**	$Sb(FP_{345})_3Ch$ (10)	1.0	rt	*vf*[i]	>99	46:54
8	**2**	$Sb(FP_{345})_3Ch$ (1)	1.0	rt	*vf*[i]	>99	30:70
9	**2**	$Sb(FP_{345})_3Ch$ (0.1)	1.0	rt	*vf*[i]	>99	25:75
10	**12**	$Sb(FP_{245})_3Ch$ (1)	1.0	rt	*vf*[i]	>99	45:55
11	**13**	$Sb(FP_{246})_3Ch$ (1)	1.0	rt	*vf*[i]	>99	40:60
12	**11**	$Sb(FP_{2\text{-}6})_3Ch$ (1)	1.0	rt	*vf*[i]	>99	68:32

[a]The general procedure for systems characterization was used, **19** as a substrate, CD_2Cl_2 as a solvent. [b]Catalysts, FP = fluorophenyls, numbers indicate the position of fluorines. In bracket, catalyst concentration in mol%. [c]Substrate concentration. [d]Reaction temperature. [e]Reaction time to reach the indicated conversion. [f]Substrate conversion determined by ^{1}H NMR spectroscopy. [g]Selectivity, B = Baldwin, A = anti-Baldwin products. Estimated error: 6%. [h]The catalyst decomposed during the reaction. [i]Very fast reaction time (<5 min).

Table S4 Condition screening with **17** as a substrate[a]

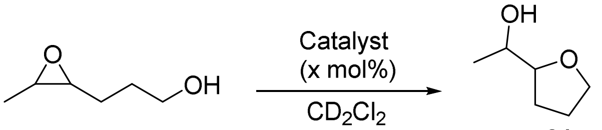

Entry		C (mol%)[b]	c (M)[c]	T (°C)[d]	t (d)[e]	η_t (%)[f]
1	-	-	0.5	rt	23	13
2	**1**	$Sb(FP_{345})_3$ (20)	0.5	rt	6	90
3	**9**	$Sb(FP_{245})_3$ (20)	0.5	rt	9	70
4	**10**	$Sb(FP_{246})_3$ (20)	0.5	rt	9	-[g]
5	**5**	$Sb(FP_{2-6})_3$ (20)	0.5	rt	9	59
6	**6**	$Bi(FP_{345})_3$ (20)	0.5	rt	9	35
7	**3**	$Sn(FP_{345})_4$ (20)	0.5	rt	9	-[g]
8	**4**	$Ge(FP_{345})_4$ (20)	0.5	rt	9	-[g]
9	**1**	$Sb(FP_{345})_3$ (50)	1.6	rt	1	60
10	**1**	$Sb(FP_{345})_3$ (500)	0.5	rt	1	93
11	**1**	$Sb(FP_{345})_3$ (20)	1.6	40	1	60
12	**1**	$Sb(FP_{345})_3$ (50)	1.6	40	1	84
13	**1**	$Sb(FP_{345})_3$ (100)	1.6	40	1	81

[a]The general procedure for systems characterization was used, **17** as a substrate, CD_2Cl_2 as a solvent. [b]Catalysts, FP = fluorophenyls, numbers indicate the position of fluorines. In bracket, catalyst concentration in mol%. [c]Substrate concentration. [d]Reaction temperature. [e]Reaction time to reach the indicated conversion. [f]Substrate conversion determined by ^1H NMR spectroscopy. [g]Similar conversion to the blank reaction.

3.2. Dependence on substrates

To a solution of the corresponding epoxide (2.0, or 2.4 M) in CD_2Cl_2 was added **1** (100 mol%), then the mixture was heated at 40 °C. The consumption of the starting material was followed by ^1H NMR spectroscopy. The conversion was calculated from the ^1H NMR spectrum of the reaction mixture by comparing the integrals of the signals assigned to the substrate and the catalyst (used also as an internal standard). The Baldwin and anti-Baldwin products were characterized as in references S7 and S8.

Table S5 Substrate comparison with **1** as a catalyst[a]

	S[b]		P[c]	t (d)[d]	η_t (%)[e]	B/A[f]
1	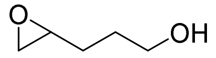 2.0 M **16**		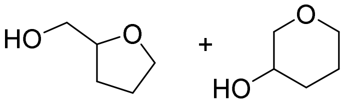 (B)-**20** (A)-**24**	1	73	100:0
2	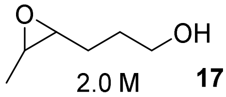 2.0 M **17**	CD_2Cl_2<br>40 °C<br>$Sb(FP_{345})_3$<br>(100 mol%)	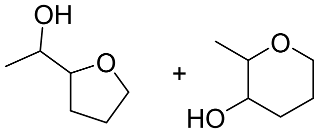 (B)-**21** (A)-**25**	1	71	100:0
3	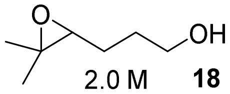 2.0 M **18**		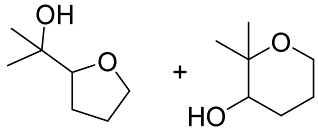 (B)-**22** (A)-**26**	3	79	4:96
4	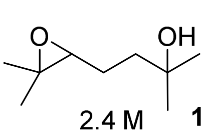 2.4 M **19**		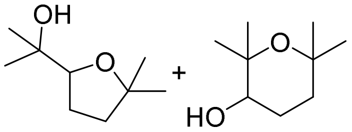 (B)-**23** (A)-**27**	1	85	56:44

[a]The general procedure for dependence on substrates was used, **1** as a catalyst (100 mol%), 40 °C, CD_2Cl_2 as a solvent. [b]Substrate and its concentration. [c]Corresponding Baldwin and anti-Baldwin product. [d]Reaction time to reach the indicated conversion. [e]Substrate conversion determined by ^1H NMR spectroscopy. [f]Selectivity, B = Baldwin, A = anti-Baldwin products. Estimated error: 6%.

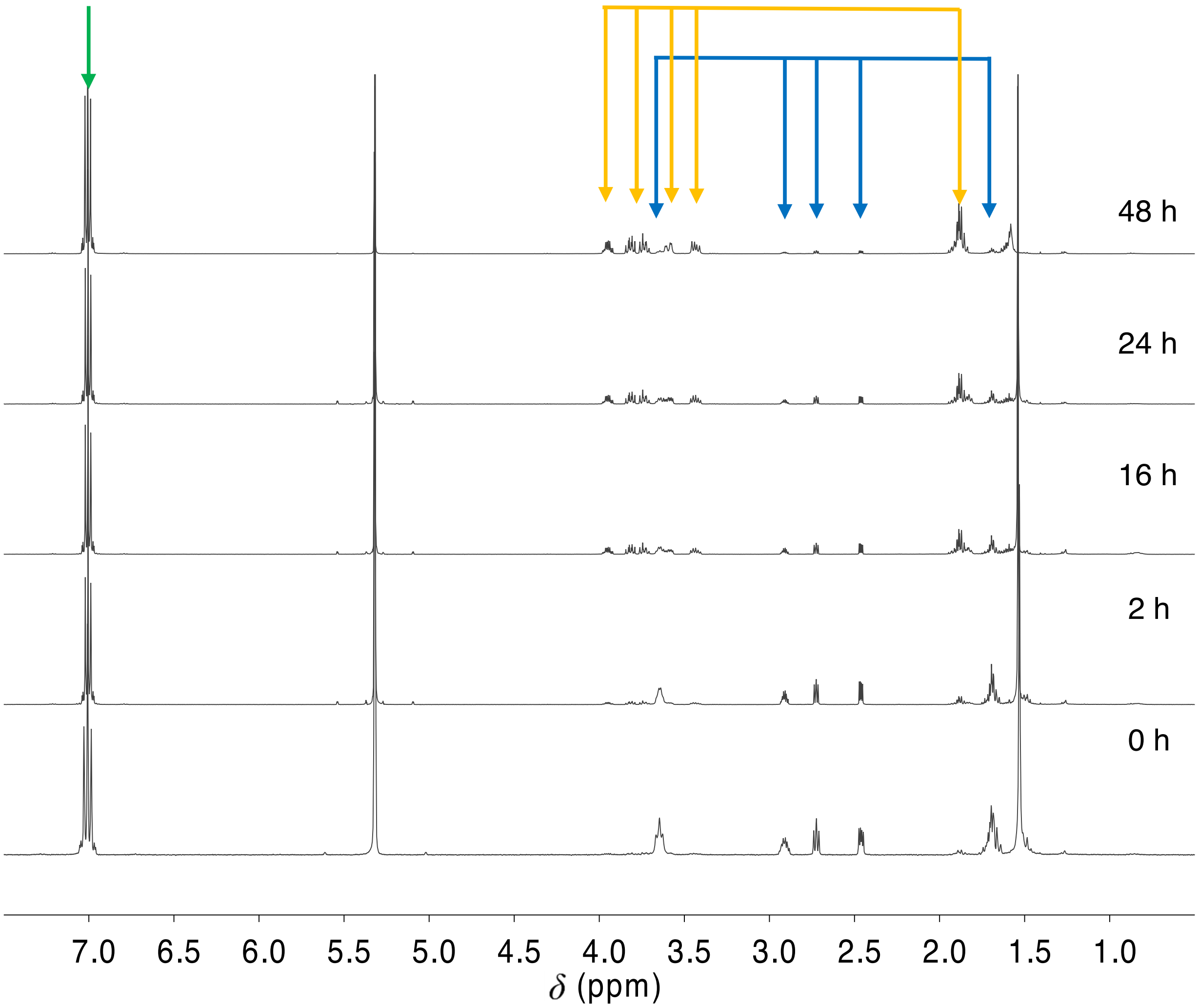

Fig. S1 ^{1}H NMR spectra of a mixture of substrate **16** (2.0 M) and **1** (100 mol%) in CD_2Cl_2 at 40 °C. The blue arrows show the consumption of **16**, the green one the catalyst, and the yellow ones the formation of the product.

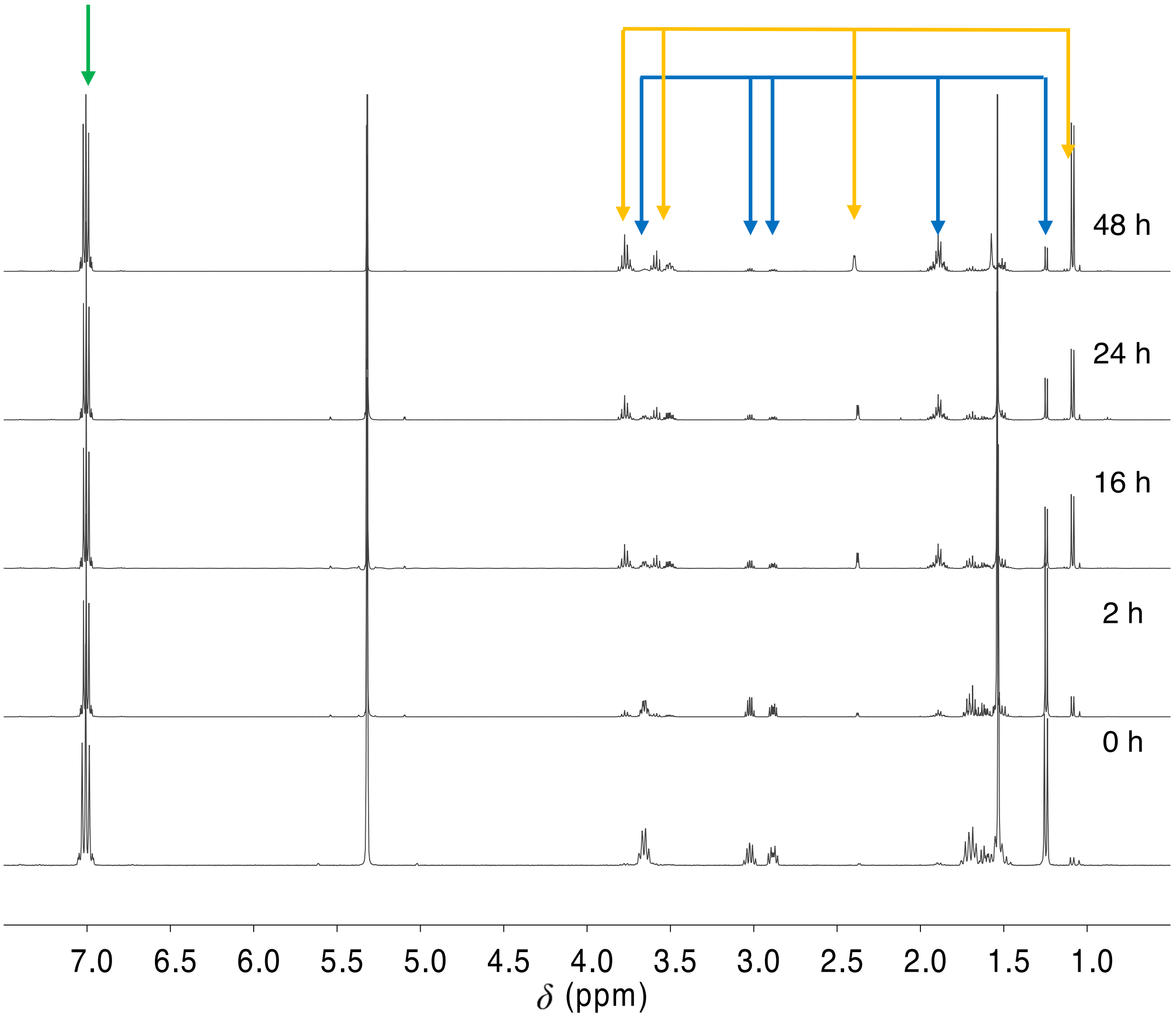

Fig. S2 ^{1}H NMR spectra of a mixture of substrate **17** (2.0 M) and **1** (100 mol%) in CD_2Cl_2 at 40 °C. The blue arrows show the consumption of **17**, the green one the catalyst, and the yellow ones the formation of the product.

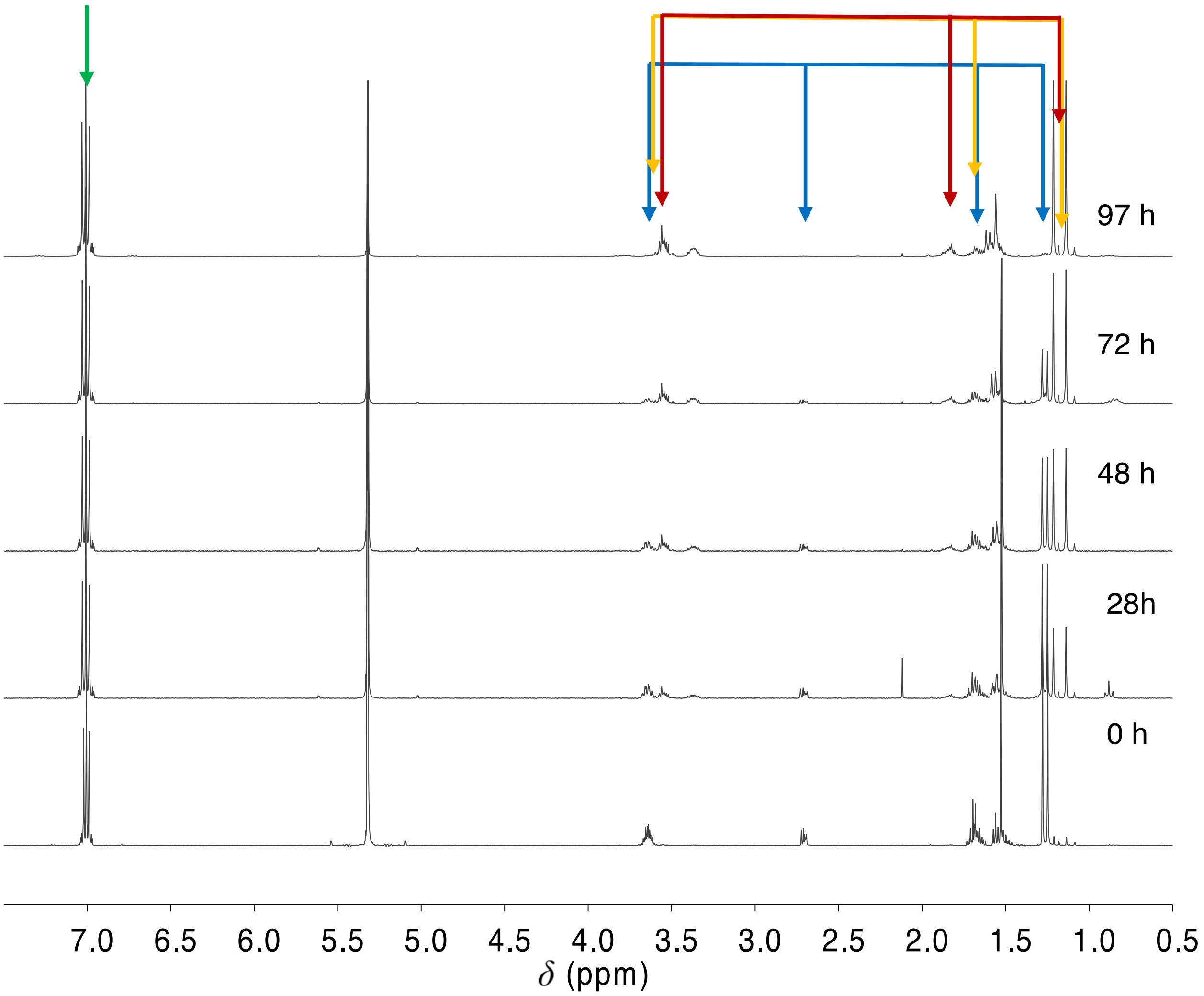

Fig. S3 ^{1}H NMR spectra of a mixture of substrate **18** (2.0 M) and **1** (100 mol%) in CD_2Cl_2 at 40 °C. The blue arrows show the consumption of **18**, the green one the catalyst, the yellow ones the formation of the Baldwin product, and the red ones the formation of the anti-Baldwin product.

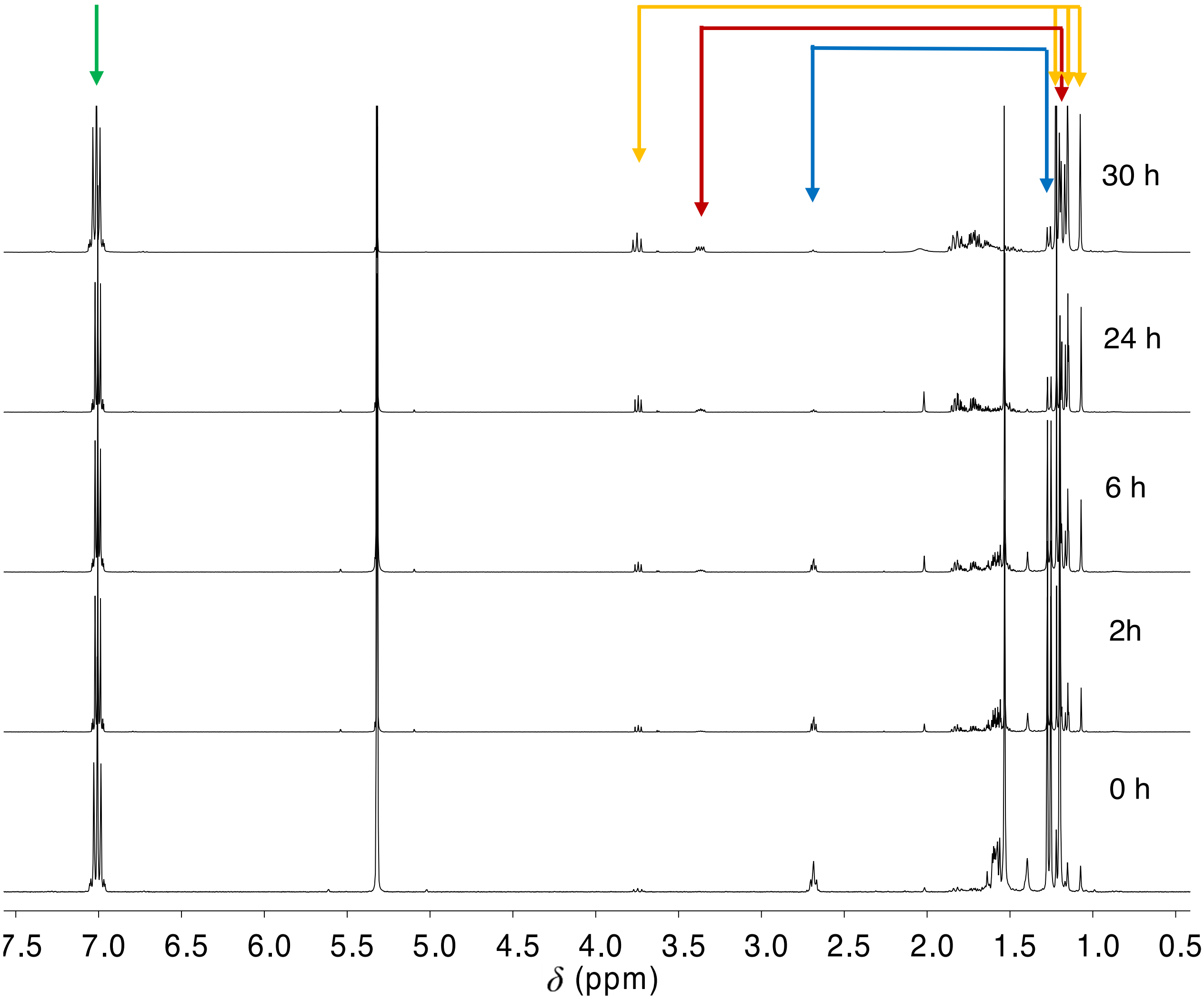

Fig. S4 ^{1}H NMR spectra of a mixture of substrate **19** (2.4 M) and **1** (100 mol%) in CD_2Cl_2 at 40 °C. The blue arrows show the consumption of **19**, the green one the catalyst, the yellow ones the formation of the Baldwin product, and the red ones the formation of the anti-Baldwin product.

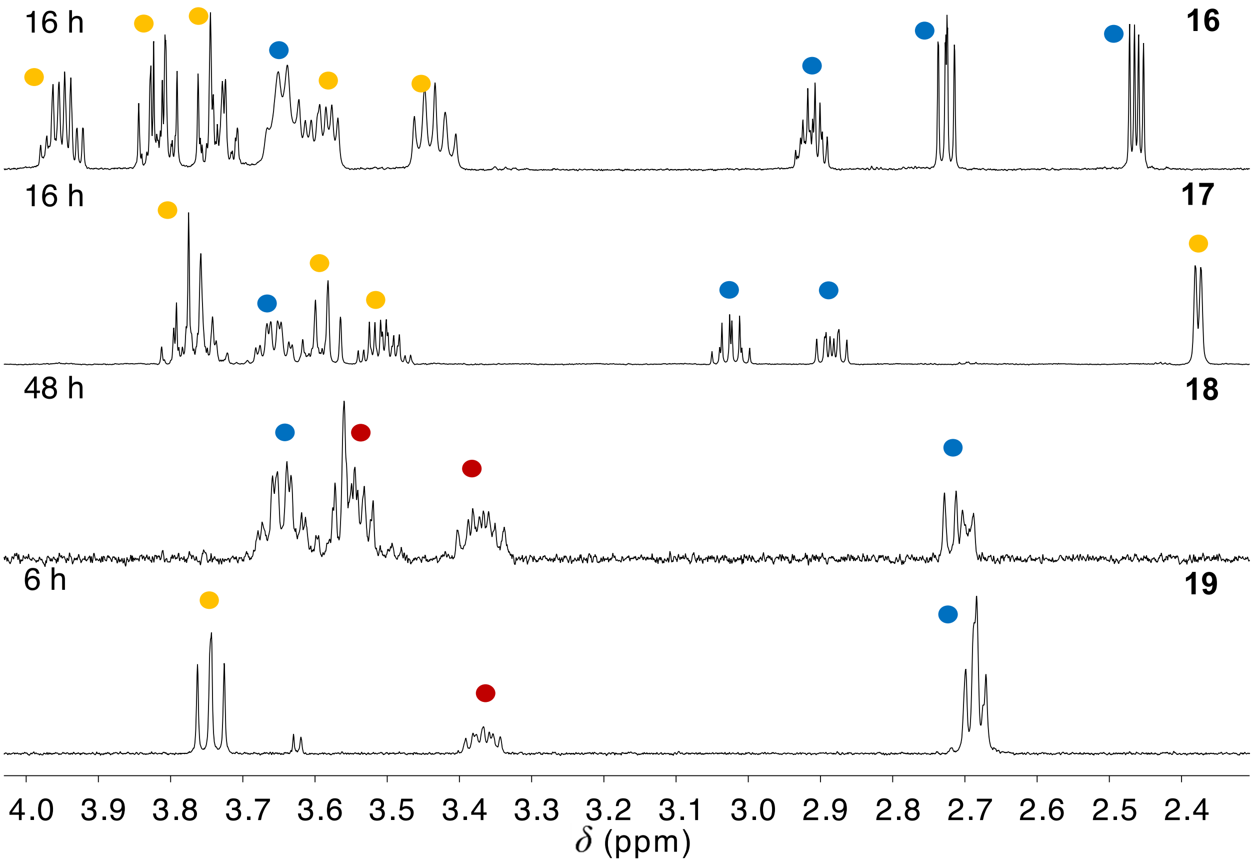

Fig. S5 Zoomed ^{1}H NMR spectra of reaction mixtures at the corresponding time (left) of the corresponding substrate from top to bottom, **16**, **17**, **18** and **19** (2.0 or 2.4 M) (blue filled circles), Baldwin product (yellow filled circles), anti-Baldwin product (red filled circles) and **1** (100 mol%) in CD_2Cl_2 at 40 °C.

3.3. Comparison with conventional catalysts

Procedure. i) To a solution of **19** (2.1 M) and CH_2Br_2 (0.3 M) in CD_2Cl_2 was added AcOH (100 mol%), then the mixture was stirred at 40 °C. The conversion was calculated from the ^1H NMR spectrum of the reaction mixture by comparing the integrals of the signals assigned to the substrate and the internal standard (CH_2Br_2). The starting material was converted only in Baldwin and anti-Baldwin cyclic ethers.

ii) To a solution of **19** (1.0 M) in CD_2Cl_2 was added $SbCl_3$ (1 mol%), then the mixture was stirred at rt. The starting material was completely consumed after < 5 minutes. The reaction mixture was diluted with CH_2Cl_2, washed twice with 1 M NaOH aqueous solution, once with water, dried over Na_2SO_4 and the solvent removed under reduced pressure. The conversion was calculated from the ^1H NMR spectrum of the reaction mixture by comparing the integrals of the signals assigned to the substrate and the products. The starting material was converted only in Baldwin and anti-Baldwin cyclic ethers.

iii) To a solution of **19** (2.4 M) in CD_2Cl_2 was added the **1** (100 mol%), then the mixture was stirred at 40 °C. The consumption of the starting material was followed by ^1H NMR spectroscopy. The conversion was calculated from the ^1H NMR spectrum of the reaction mixture by comparing the integrals of the signals assigned to the substrate and the catalyst (used also as internal standard). The starting material was converted only in Baldwin and anti-Baldwin cyclic ethers.

iv) To a solution of **19** (1.0 M) in CD_2Cl_2 was added the corresponding catalyst (1 mol%), then the mixture was stirred at rt. The consumption of the starting material was followed by ^1H NMR spectroscopy. The conversion was calculated from the ^1H NMR spectrum of the reaction mixture by comparing the integrals of the signals assigned to the substrate and the products. The starting material was converted only in Baldwin and anti-Baldwin cyclic ethers.

Table S6 Activities of Brønsted and Lewis acids and pnictogen bonding catalysts

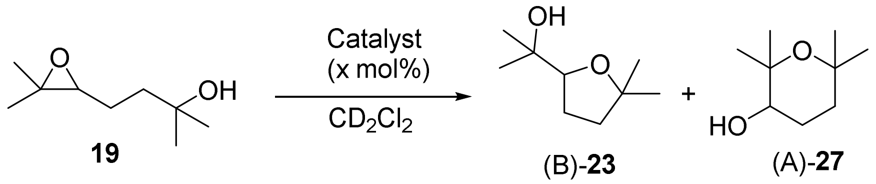

Entry		C (mol%)[a]	*c* (M)[b]	*T* (°C)[c]	*t* (h)[d]	η_t (%)[e]	B/A[f]
1[g]	-	AcOH (100)	2.1	rt	30	>99	93:7
2[g]	-	AcOH (100)	2.1	40	18	>99	92:8
3[h]	-	$SbCl_3$ (100)	2.4	rt	< 0.1	>99	76:24
4[h]	-	$SbCl_3$ (1)	1.0	rt	< 0.1	>99	80:20
5[i]	**1**	$Sb(FP_{345})_3$ (100)	2.4	40	24	81	56:44
6[j]	**2**	$Sb(FP_{345})_3Ch$ (1)	1.0	rt	< 0.1	>99	30:70
7[j]	**11**	$Sb(FP_{2\text{-}6})_3Ch$ (1)	1.0	rt	< 0.1	>99	68:32

[a]Catalysts, FP = fluorophenyls, numbers indicate the position of fluorines. In bracket, catalyst concentration in mol%. [b]Substrate concentration. [c]Reaction temperature. [d]Reaction time to reach the indicated conversion. [e]Substrate conversion determined by ^{1}H NMR spectroscopy. [f]Selectivity, B = Baldwin, A = anti-Baldwin products. Estimated error: 6%. [g]Procedure i) was followed. [h]Procedure ii) was followed. [i]Procedure iii) was followed. [j]Procedure iv) was followed.

3.4. Kinetics

Procedure. i) To a solution of **17** (1.6 M) in CD_2Cl_2 was added **1** (100 mol%), then the mixture was heated at 40 °C. The consumption of the starting material was followed by ^{1}H NMR spectroscopy. The substrate concentration was calculated from the ^{1}H NMR spectrum of the reaction mixture by comparing the integrals of the signals assigned to the substrate and the catalyst (used also as an internal standard). The starting material was converted only in Baldwin and/or anti-Baldwin cyclic ethers.

ii) To a solution of **17** (0.16 M) in CD_2Cl_2 in an NMR tube at -78 °C was added a stock solution of the corresponding catalyst (1 mol%). The NMR tube was quickly inserted in the spectrometer (approximatively 10 s). The progress of the reaction at -30 °C was monitored by measuring ^{1}H NMR spectra every 5, 10, 20, or 60 s. The substrate concentration was calculated by comparing the integrals of the signals assigned to the substrate and the product.

Kinetic studies. The pseudo-first-order rate constant (*k*) was estimated by fitting the data to the equation (S1):

$$c^{\mathrm{s}} = c_0^{\mathrm{s}} e^{(-kt)} \qquad \text{(S1)}$$

where c^s corresponds to the substrate concentration and c_0^s to the substrate concentration at $t = 0$. When $SbCl_3$ was used as a catalyst its slight decomposition led to a poor fit with equation (S1). Thus, the rate constant *k* was approximated from the equation:

$$k = v_{\mathrm{ini}}/c_0^{\mathrm{s}} \qquad \text{(S2)}$$

where v_{ini} is the initial velocity, calculated from the equation:

$$c^{\mathrm{s}} = c_0^{\mathrm{s}} - v_{\mathrm{ini}}t \qquad \text{(S3)}$$

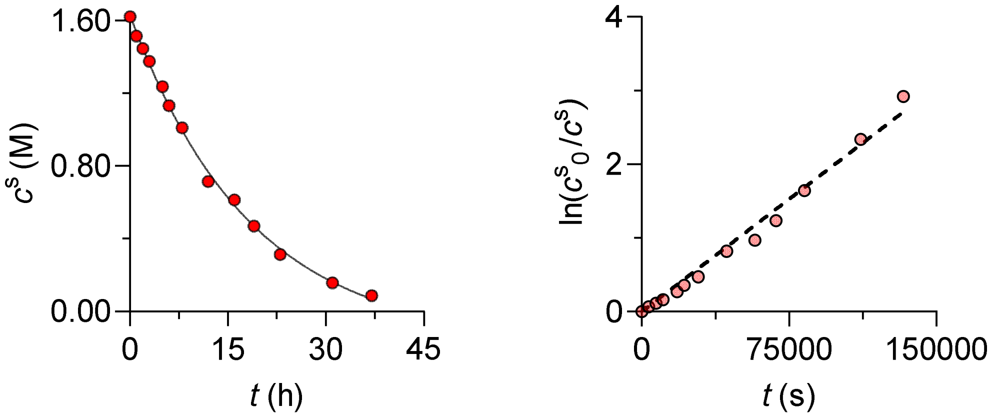

Fig. S6 Left: The conversion of substrate **17** (c_0^s = 1.6 M) over time using **1** as a catalyst (100 mol%) at 40 °C. Right: Plot of [$\ln(c_0^s/c^s)$] against t (s). The slope of the linear fit corresponds to the rate constant k.

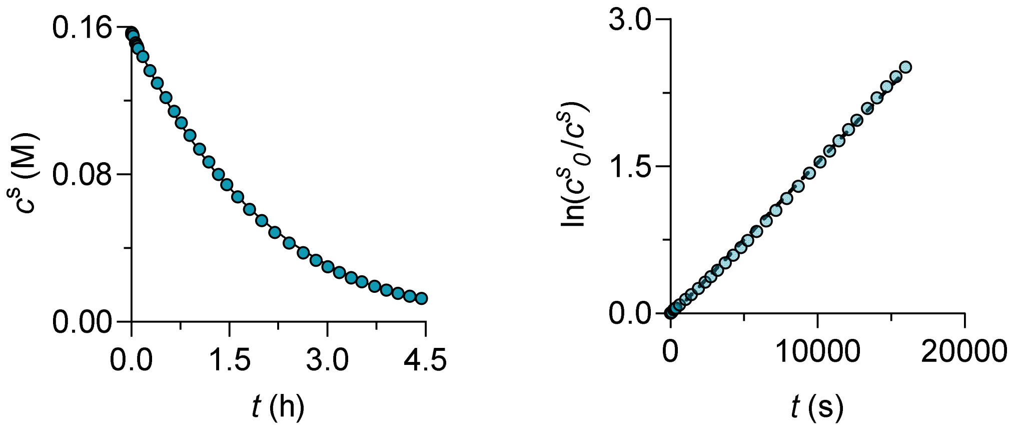

Fig. S7 Left: The conversion of substrate **17** (c_0^s = 0.16 M) over time using **2** as a catalyst (1 mol%) at -30 °C against t (s). Right: Plot of [$\ln(c_0^s/c^s)$]. The slope of the linear fit corresponds to the rate constant k.

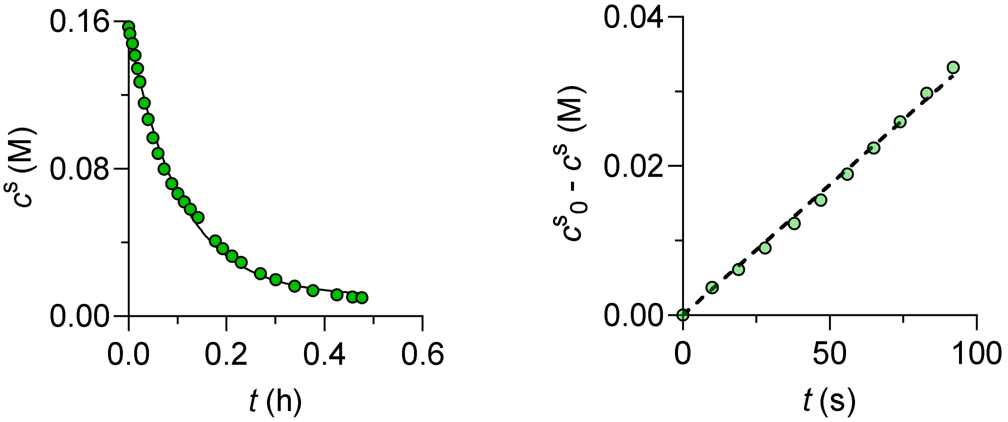

Fig. S8 Left: The conversion of substrate **17** (c_0^s = 0.16 M) over time using **2** as a catalyst (1 mol%) at -30 °C against *t* (s). Right: Initial velocity of the reaction.

Table S7 Kinetic studies with substrate **17**.

Entry		C (mol%)[a]	T (°C)[b]	t (h)[c]	η_t (%)[d]	k (s^{-1})[e]
1[f]	**1**	$Sb(FP_{345})_3$ (100)	40	37	95	2.0 x 10^{-5}
2[g]	**2**	$Sb(FP_{345})_3Ch$ (1)	-30	4.5	92	1.6 x 10^{-4}
3[g]	-	$SbCl_3$ (1)	-30	0.5	93	2.2 x10^{-4}

[a]Catalysts, FP = fluorophenyls, numbers indicate the position of fluorines. In bracket, catalyst concentration in mol%. [b]Reaction temperature. [c]Reaction time to reach the indicated. [d]Substrate conversion determined by ^{1}H NMR spectroscopy. [e]Kinetic constant. [f]Procedure i) was followed. [g]Procedure ii) was followed.

4. Diepoxide substrates

4.1. Systems characterization

General procedure. To a solution of **28** in CD_2Cl_2 was added the corresponding catalyst, then the mixture was heated at the corresponding temperature. The consumption of the epoxides was followed by ^1H NMR spectroscopy. The conversion was calculated from the ^1H NMR spectrum of the reaction mixture by comparing the integrals of the signals assigned to epoxides and the catalyst (used also as internal standard). When the catalyst signal was not detectable, CH_2Br_2 was added as an internal standard (0.3 M).

Table S8 Condition screening for substrate **28** with **1** as a catalyst[a]

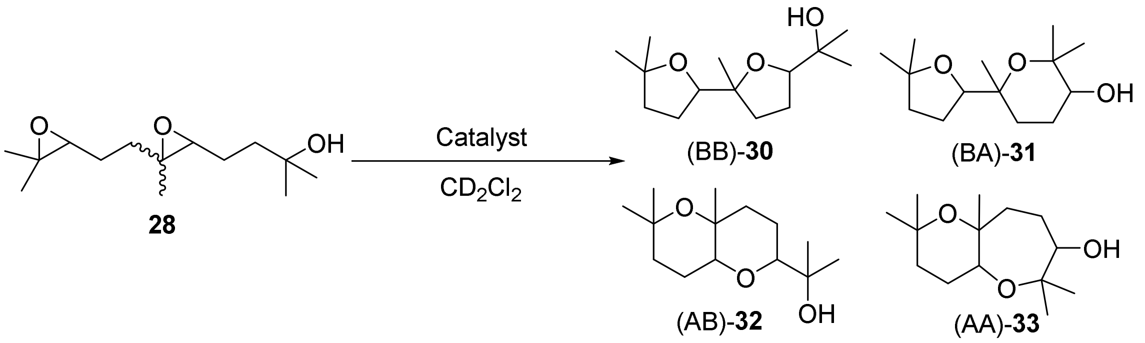

Entry		C (mol%)[b]	*c* (M)[c]	*T* (°C)[d]	*t* (d)[e]	η_t (%)[f]
1	**1**	$Sb(FP_{345})_3$ (20)	2.0	rt	24	80
2	**1**	$Sb(FP_{345})_3$ (500)	0.5	rt	11	66
3	**1**	$Sb(FP_{345})_3$ (100)	2.0	40	8	83
4	**1**	$Sb(FP_{345})_3$ (500)	0.5	60	2	93

[a]The general procedure for systems characterization was used, **28** as a substrate, CD_2Cl_2 as the solvent. [b]Catalysts, FP = fluorophenyls, numbers indicate the position of fluorines. In bracket, catalyst concentration in mol%. [c]Substrate concentration. [d]Reaction temperature. [e]Reaction time to reach the indicated conversion. [f]Epoxide conversion determined by ^1H NMR spectroscopy.

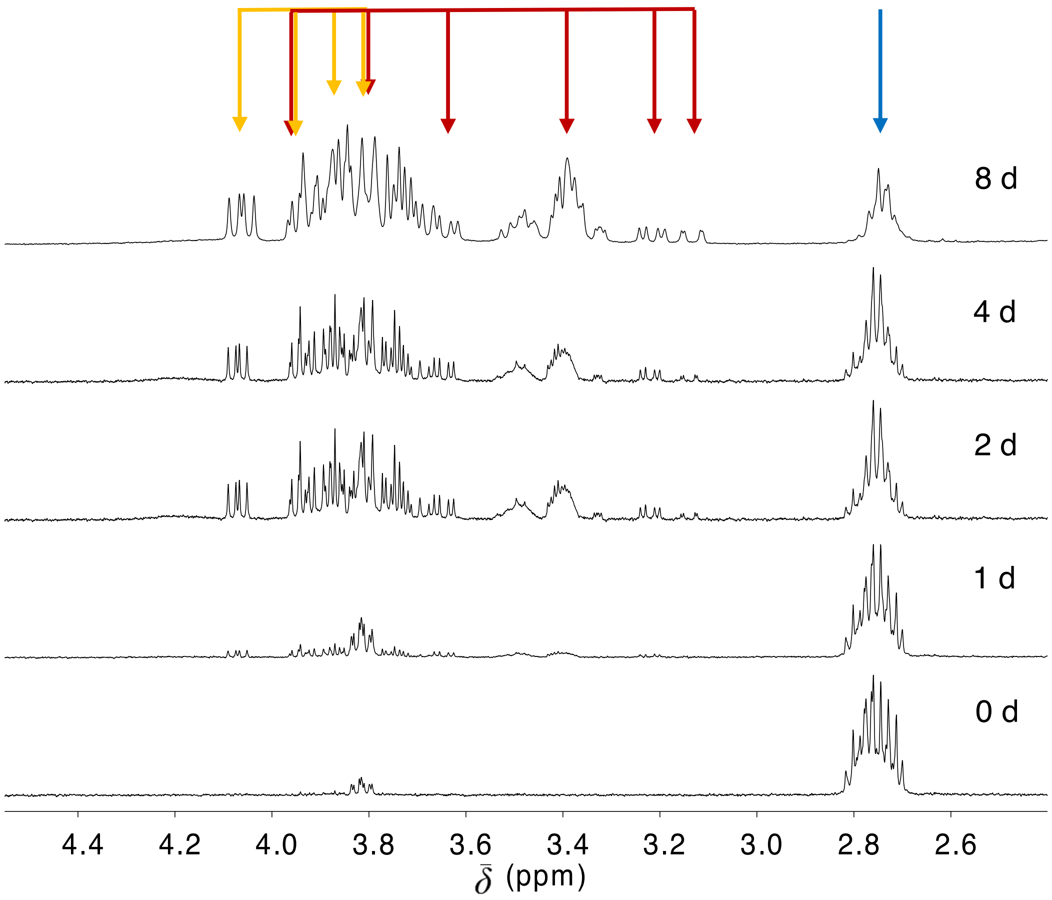

Fig. S9 ^{1}H NMR spectra of a mixture of substrate **28** (2.0 M) and **1** (100 mol%) in CD_2Cl_2 at 40 °C. The blue arrow shows the consumption of epoxides in **28** and the intermediates, the yellow ones the formation of the BB cyclization product, and the red ones the formation of the AB, BA, and/or AA products.

Table S9 Catalyst screening with substrate **28**[a]

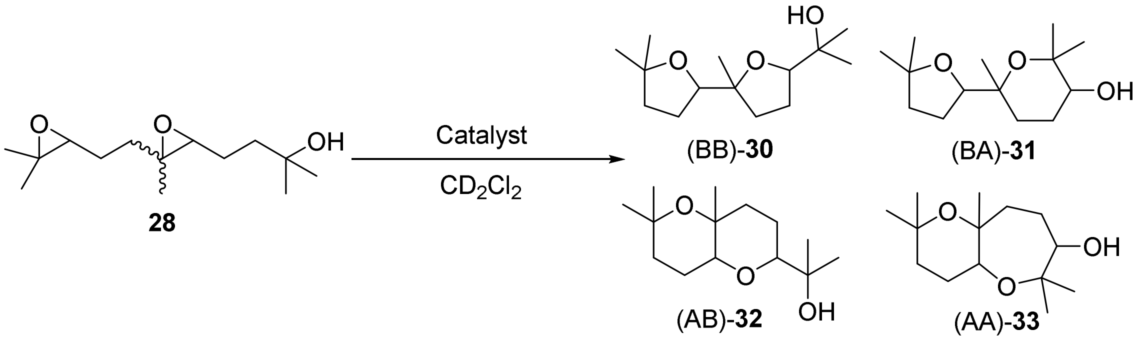

Entry		C (mol%)[b]	c (M)[c]	T (°C)[d]	t (d)[e]	η_t (%)[f]
1	**1**	Sb(FP$_{345}$)$_3$ (100)	2.0	40	8	83
2	**9**	Sb(FP$_{245}$)$_3$ (100)	2.0	40	8	89
3	**10**	Sb(FP$_{246}$)$_3$ (100)	2.0	40	8	55
4[g]	**5**	Sb(FP$_{2\text{-}6}$)$_3$ (100)	2.0	40	-	-
5	**2**	Sb(FP$_{345}$)$_3$Ch (1)	1.0	rt	*vf*[h]	97
6	**11**	Sb(FP$_{2\text{-}6}$)$_3$Ch (1)	1.0	rt	*vf*[i]	97

[a]The general procedure for systems characterization was used, **28** as a substrate, CD_2Cl_2 as the solvent. [b]Catalysts, FP = fluorophenyls, numbers indicate the position of fluorines. In bracket, catalyst concentration in mol%. [c]Substrate concentration. [d]Reaction temperature. [e]Reaction time to reach the indicated conversion. [f]Epoxides conversion determined by ^{1}H NMR spectroscopy. [g]The catalyst decomposed during the reaction. [h]Very fast reaction time in day scale (2 h). [i]Very fast reaction time in day scale (1 h).

4.2. Product identification

Procedure. To a solution of **28** (3.0 mmol) in CD_2Cl_2 (3 mL) was added **2** (1 mol%), then the mixture was stirred at rt for 2 h. The solvent was evaporated under reduced pressure. The various isomers were separated via iterative silica gel column chromatography of the first purification using pentane/EtOAc as a mobile phase. First purification (gradient): pentane/EtOAc 4:1 (700 mL, fraction A-F); pentane/EtOAc 1:1 (200 mL, fraction F-H). Further purification (for fractions B, E, F, G): pentane/EtOAc 3:2. The product *trans,trans* (AA)-**33** was recrystallized from fraction C using pentane.

trans,trans (BB)-**30** and *trans,cis* (BB)-**30**. R_f (EtOAc/Pentane 1:1): 0.57. *cis,trans* (BB)-**30**. R_f (EtOAc/Pentane 1:1): 0.50; NMR: as reported in reference S10. *cis,cis* (BB)-**30**. From fraction B. R_f (EtOAc/Pentane 1:1): 0.54; NMR: as reported in reference S10.

(BA)-**31**: isomer-a. From fraction B. R_f (EtOAc/Pentane 1:1): 0.54; NMR: as reported in reference S8. (BA)-**31**, isomer-b. From fractions D and E. R_f (EtOAc/Pentane 1:1): 0.46; ^{1}H NMR (400 MHz, $CDCl_3$): 3.75 (t, $^3J_{H-H}$ = 7.2 Hz, 1H), 3.45 – 3.34 (m, 1H), 2.04 – 1.39 (m, 10H), 1.24 (s, 3H), 1.23 (s, 3H), 1.23 (s, 3H), 1.22 (s, 3H), 1.20 (s, 3H); ^{13}C NMR (101 MHz, $CDCl_3$): 86.0 (CH), 81.1 (C), 75.5 (CH), 74.8 (C), 74.4 (C), 38.5 (CH_2), 30.4 (CH_2), 29.9 (CH_3), 28.6 (CH_3), 27.9 (CH_3), 26.5 (CH_2), 25.0 (CH_2), 23.0 (CH_3), 21.6 (CH_3). (BA)-**31**, isomer-c. From fractions D-F. R_f (EtOAc/Pentane 1:1): 0.44; ^{1}H NMR (300 MHz, $CDCl_3$): 3.94 (t, $^3J_{H-H}$ = 7.2 Hz, 1H), 3.42 (dd, $^3J_{H-H}$ = 5.6 Hz, 2.9 Hz, 1H), 2.00 – 1.36 (m, 10H), 1.27 (s, 3H), 1.26 (s, 13H), 1.21 (s, 3H), 1.20 (s, 3H), 1.14 (s, 3H).

(AB)-**32**, isomer-a. From fractions A and B. R_f (EtOAc/Pentane 1:1): 0.60; ^{1}H NMR (400 MHz, $CDCl_3$): 3.32 (dd, $^3J_{H-H}$ = 3.7 Hz, $^3J_{H-H}$ = 2.2 Hz, 1H), 3.13 (dd, $^3J_{H-H}$ = 11.5 Hz, $^3J_{H-H}$ = 1.9 Hz, 1H), 2.76 (s, 1H), 2.09 – 1.98 (m, 1H), 1.93 – 1.82 (m, 2H), 1.76 – 1.65 (m, 1H), 1.68 – 1.57 (m, 1H), 1.35 – 1.30 (m, 2H), 1.28 (dd, $^3J_{H-H}$ = 4.0 Hz, $^3J_{H-H}$ = 2.8 Hz, 1H), 1.26 (s, 3H), 1.20 (s,

6H), 1.19 (s, 3H), 1.16 (s, 3H); ^{13}C NMR (101 MHz, $CDCl_3$): 83.1 (CH), 74.1 (CH), 72.1 (C), 71.2(C), 69.0 (C), 38.7 (CH_2), 33.4 (CH_3), 30.1 (CH_2), 28.3 (CH_3), 26.8 (CH_3), 25.5 (CH_3), 23.7 (CH_3), 22.4 (CH_2), 21.9 (CH_2). (AB)-**32**, isomer-b. From fraction G. ^{1}H NMR (300 MHz, $CDCl_3$): 3.47 (dd, $^3J_{H\text{-}H}$ = 11.1 Hz, $^3J_{H\text{-}H}$ = 2.6 Hz, 1H), 3.41 (t, $^3J_{H\text{-}H}$ = 3.7 Hz, 1H), 2.03 – 1.91 (m, 2H), 1.84 – 1.74 (m, 2H), 1.71 – 1.64 (m, 2H), 1.57 – 1.49 (m, 2H), 1.29 (s, 3H), 1.22 (s, 3H), 1.21 (s, 6H), 1.14 (s, 3H); ^{13}C NMR (101 MHz, $CDCl_3$): 80.1 (CH), 77.1 (C), 75.2 (C), 71.0 (C), 68.6 (CH), 38.7 (CH_2), 33.1 (CH_3), 30.2 (CH_3), 30.0 (CH_2), 28.3 (CH_2), 27.5 (CH_3), 26.8 (CH_3), 24.5 (CH_2), 17.7 (CH_3). (AB)-**32**, isomer-c. From fraction B. R_f (EtOAc/Pentane 1:1): 0.54. Characteristic peaks: 3.64 (dd, $^3J_{H\text{-}H}$ = 11.4 Hz, $^3J_{H\text{-}H}$ = 4.1 Hz, 1H), 3.41 (dd, $^3J_{H\text{-}H}$ = 7.8 Hz, $^3J_{H\text{-}H}$ = 2.6 Hz, 1H).

trans,trans (AA)-**33**. Isolated from fractions B-D. R_f (EtOAc/Pentane 1:1): 0.49; ^{1}H NMR (500 MHz, $CDCl_3$): 3.88 – 3.74 (m, 1H), 3.63 (dd, $^3J_{H\text{-}H}$ = 11.7 Hz, $^3J_{H\text{-}H}$ = 4.4 Hz, 1H), 1.94 – 1.85 (m, 1H), 1.85 – 1.78 (m, 2H), 1.72 – 1.63 (m, 1H), 1.63 – 1.57 (m, 1H), 1.55 – 1.45 (m, 2H), 1.28 (s, 3H), 1.25 (s, 3H), 1.24 (s, 3H), 1.17 (s, 3H), 1.11 (s, 3H); ^{13}C NMR (126 MHz, $CDCl_3$): 78.1 (C), 76.7 (C), 76.6 (CH), 73.6 (CH), 71.1(C), 37.4 (CH_2), 36.3 (CH_2), 33.2 (CH_3), 28.8 (CH_3), 27.2 (CH_3), 25.5 (CH_2), 25.1 (CH_2), 22.1 (CH_3), 20.0 (CH_3). *trans,cis* (AA)-**33**. From fractions E and F. R_f (EtOAc/Pentane 1:1): 0.37; ^{1}H NMR (500 MHz, $CDCl_3$): 3.76 (dd, $^3J_{H\text{-}H}$ = 10.6 Hz, $^3J_{H\text{-}H}$ = 1.2 Hz, 1 H), 3.21 (dd, $^3J_{H\text{-}H}$ = 11.7 Hz, $^3J_{H\text{-}H}$ = 4.3 Hz, 1H), 1.96 – 1.41 (m, 10H), 1.29 (s, 3H), 1.25 (s, 3H), 1.22 (s, 3H), 1.16 (s, 3H); ^{13}C NMR (126 MHz, $CDCl_3$): 78.9 (CH), 77.2 (C), 76.1 (C), 72.9 (CH), 71.2 (C), 42.7 (CH_2), 37.5 (CH_2), 33.2 (CH_3), 29.9 (CH_2), 27.1 (CH_3), 25.0 (CH_3), 24.9 (CH_2), 21.7 (CH_3), 20.3 (CH_3).

(A)-**29**, isomer-a. From fraction H. R_f (EtOAc/Pentane 1:1): 0.31; ^{1}H NMR (300 MHz, $CDCl_3$): 3.62 – 3.38 (m, 1H), 2.76 (t, $^3J_{H\text{-}H}$ = 6.0 Hz, 1H), 1.83 – 1.72 (m, 3H), 1.71 – 1.53 (m, 7H), 1.31 (s, 3H), 1.29 (s, 3H), 1.23 (s, 3H), 1.21 (s, 3H), 1.16 (s, 3H).

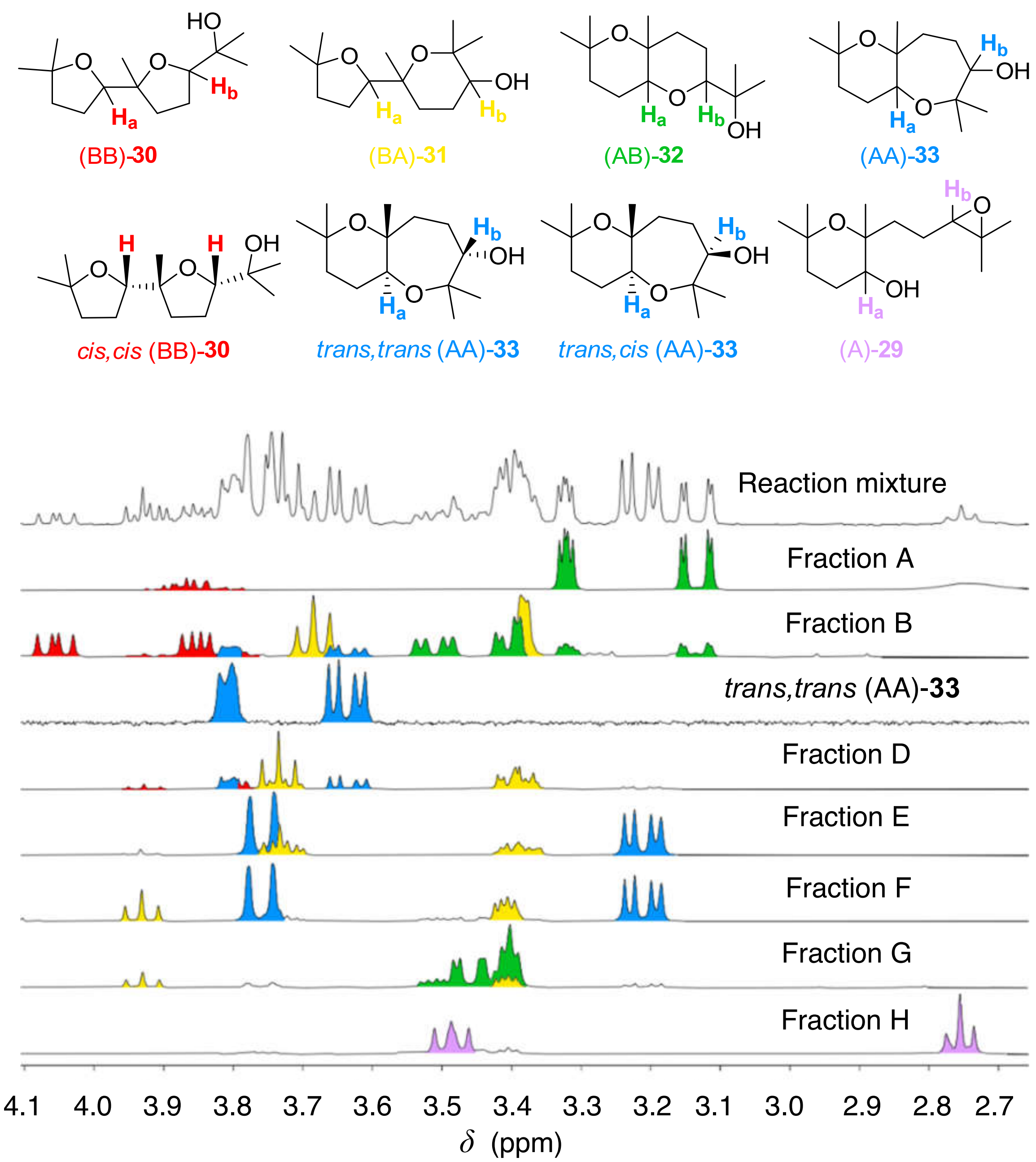

Fig. S10 Structures and ^{1}H NMR spectra of the cyclization products obtained from **28** and **2**. ^{1}H NMR spectra are of the reaction mixture, chromatographic fractions, and pure *trans*,*trans* (AA)-**33**. Identified characteristic peaks are colored according to the color code used in the structures.

4.3. Comparison with conventional catalysts

Procedure. i) To a solution of **28** (2.1 M) and CH_2Br_2 (0.3 M) in CD_2Cl_2 was added AcOH (100 mol%), then the mixture was stirred at 40 °C. The conversion was calculated from the ^1H NMR spectrum of the reaction mixture by comparing the integrals of the signals assigned to the epoxides and the internal standard (CH_2Br_2). The starting material was converted only in Baldwin poly-cyclic ethers.[S9]

ii) To a solution of **28** (1.0 M) in CD_2Cl_2 was added $SbCl_3$ (x mol%), then the mixture was stirred at rt. The reaction mixture was diluted with $CDCl_3$, washed twice with 1 M NaOH aqueous solution, once with water, and dried over Na_2SO_4. The conversion was calculated from the ^1H NMR spectrum of the reaction mixture by comparing the integrals of the signals assigned to the epoxides and the products. The starting material was converted only in Baldwin and anti-Baldwin poly-cyclic ethers.

iii) To a solution of **28** (2.0 M) in CD_2Cl_2 was added the corresponding catalyst (500 mol%), then the mixture was stirred at 60 °C. The consumption of the starting material was followed by ^1H NMR spectroscopy. The conversion was calculated from the ^1H NMR spectrum of the reaction mixture by comparing the integrals of the signals assigned to the epoxides and the catalyst (used also as internal standard). The starting material was converted only in Baldwin and anti-Baldwin poly-cyclic ethers.

iv) To a solution of **28** (1.0 M) in CD_2Cl_2 was added the corresponding catalyst (1 mol%), then the mixture was stirred at rt. The consumption of the starting material was followed by 1H NMR spectroscopy. The conversion was calculated from the ^1H NMR spectrum of the reaction mixture by comparing the integrals of the signals assigned to the epoxides and the internal standard (CH_2Br_2). The starting material was converted only in Baldwin and anti-Baldwin poly-cyclic ethers.

Table S10 Selectivity evaluation with **28**

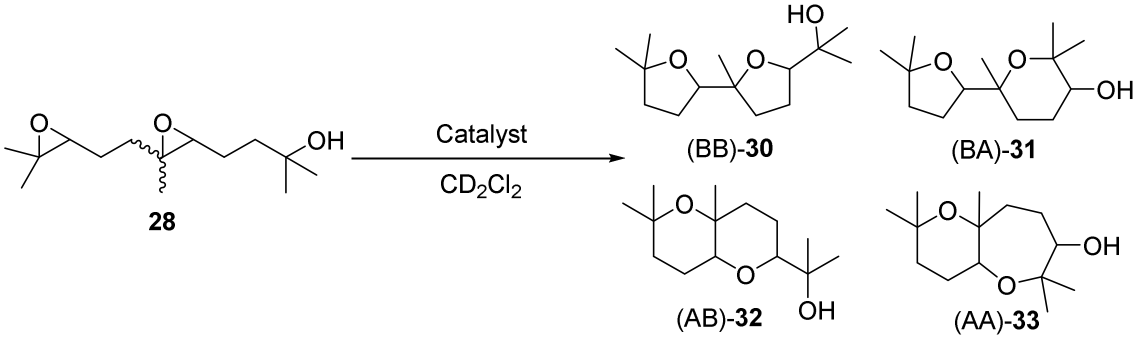

Entry	C (mol%)[a]	c (M)[b]	T (°C)[c]	t (h)[d]	η_t (%)[e]	BB/AA[f]	*tt*/*tc*[g]
1[h]	AcOH (100)	1.0	rt	24	97	99:1[i]	-
2[j]	$SbCl_3$(1)	1.0	rt	30	<60[k]	83:17	69:31
3[j]	$SbCl_3$ (100)	1.0	rt	< 0.5	>97	79:21	53:47
4[l]	**1** (500)	0.5	60	48	93	27:73	49:51
5[m]	**2** (1)	1.0	rt	2	97	11:89	36:64
6[m]	**11** (1)	1.0	rt	1	97	61:39	39:61

[a]Catalysts, FP = fluorophenyls, numbers indicate the position of fluorines. In bracket, catalyst concentration in mol%. [b]Substrate concentration. [c]Reaction temperature. [d]Reaction time to reach the indicated conversion. [e]Epoxides conversion determined by ^{1}H NMR spectroscopy. [f]Selectivity, BB: *cis,cis* (BB)-**30** product, AA: *trans,trans* (AA)-**33** product, derived from the same *trans,syn* **28** isomer. Estimated error: 6%. [g]Selectivity, *tt*: *trans,trans* (AA)-**33** product (derived from *trans,syn* **28**), *tc*; *trans,cis* (AA)-**33** product (derived from *trans,anti* **28**). Estimated error: 6%. [h]Procedure i) was followed. [i]Traces of anti-Baldwin products. [j]Procedure ii) was followed. [k]The reaction stops due to the decomposition of the catalyst (white precipitate). [l]Procedure iii) was followed. [m]Procedure iv) was followed.

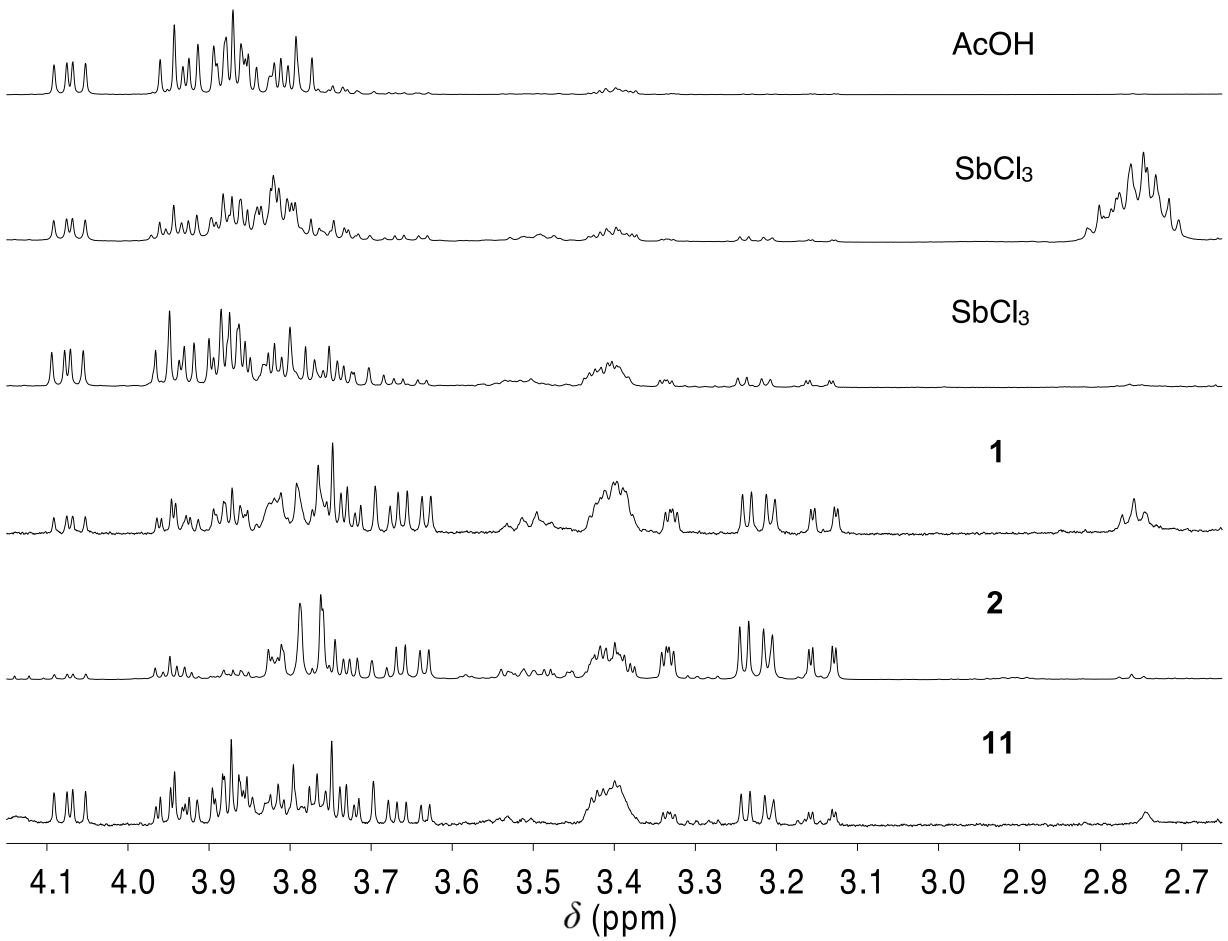

Fig. S11 Comparison of ^{1}H NMR spectra of reaction mixtures from the cyclization of **28** using different catalysts. In order from top: AcOH (100 mol%) at 40 °C, $SbCl_3$ (1 mol%) at rt, $SbCl_3$ (100 mol%) at rt, **1** (500 mol%) at 60 °C, **2** (1 mol%) at rt, **11** (1 mol%) at rt.

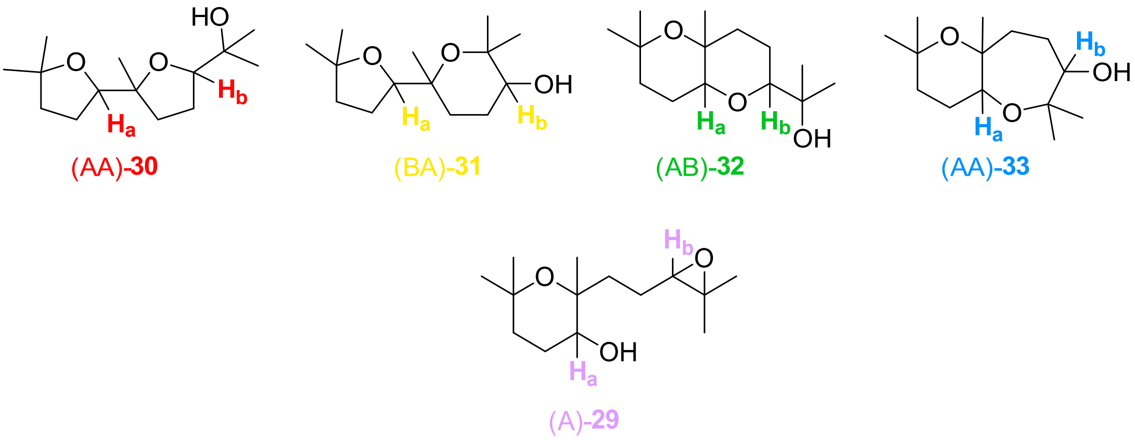

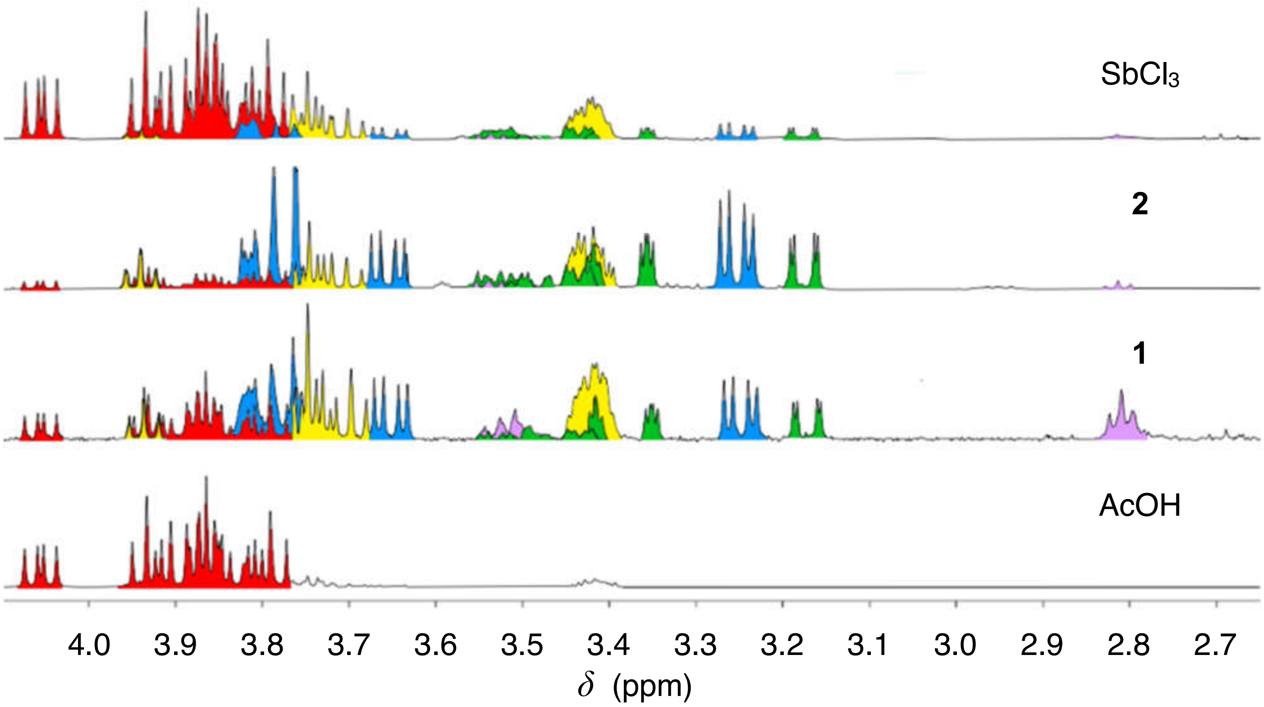

Fig. S12 ^{1}H NMR spectra of product mixtures obtained from **28** with different catalysts. In order from top: $SbCl_3$ (100 mol%) at rt, **2** (1 mol%) at rt, **1** (500 mol%) at 60 °C, AcOH (100 mol%) at 40 °C. Signals are colored according to the structures of the products.

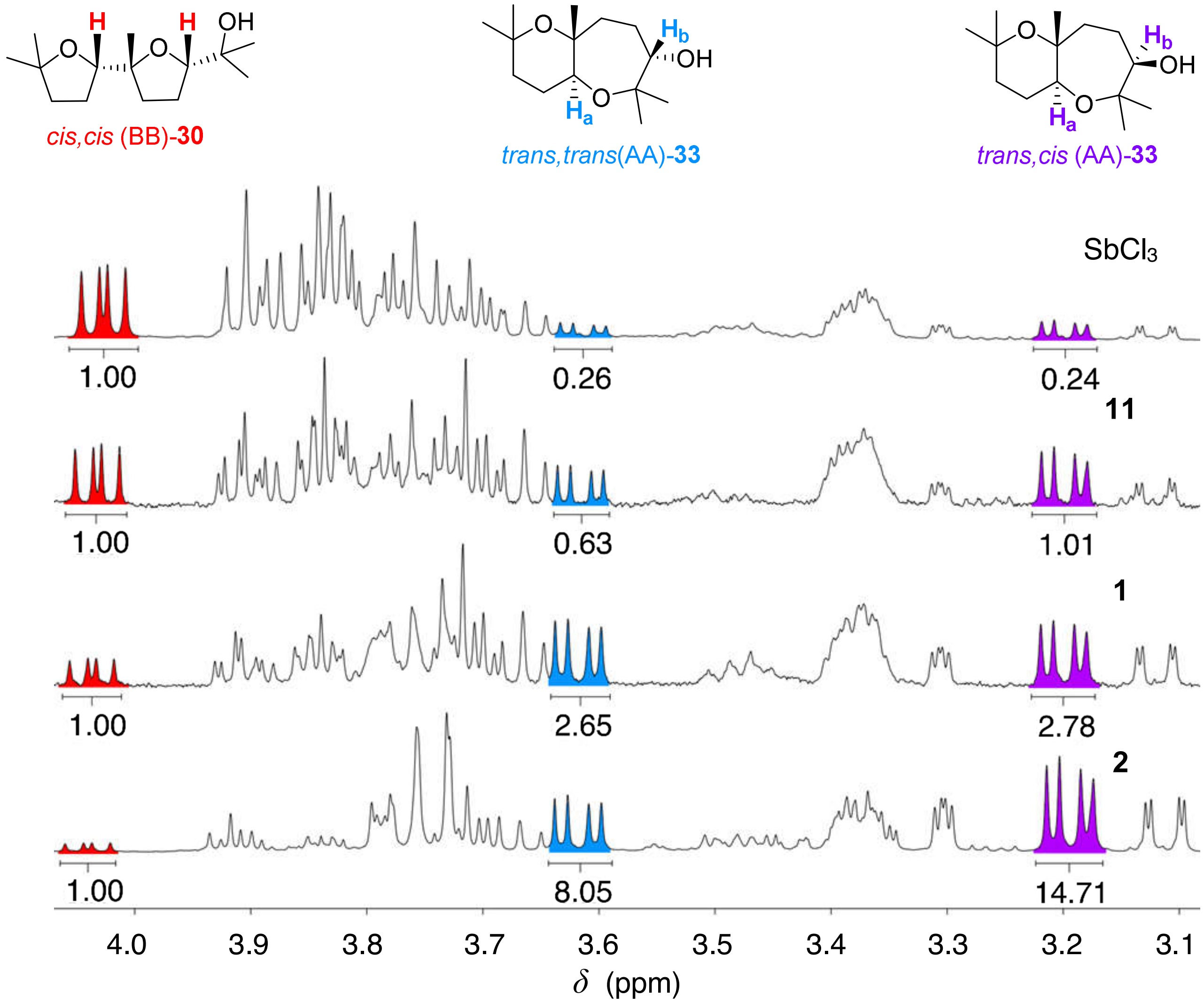

Fig. S13. Close-up of Fig. S12 for the evaluation of *cis,cis* (BB)-**30** (red), *trans,trans* (AA)-**33** (blue), both formed from the same *trans,syn* **28** diepoxide and *trans,cis* (AA)-**33** (purple), formed from *trans,anti* **28** diepoxide. In order from top: $SbCl_3$ (100 mol%) at rt, **11** (1 mol%) at rt, **1** (500 mol%) at 60 °C, **2** (1 mol%) at rt.

5. Tri- and tetraepoxide oligomers

5.1. Systems characterization

Procedure. i) To a solution of the corresponding oligoepoxide (0.5 M) in CD_2Cl_2 was added **1** (500 mol%), then the mixture was heated at 60 °C. The consumption of the starting material was followed by ^1H NMR spectroscopy. The conversion of epoxides was calculated from the ^1H NMR spectrum of the reaction mixture by comparing the integrals of the signals assigned to epoxides and the catalyst.

ii) To a solution of the corresponding oligoepoxide (1.0 M) in CD_2Cl_2 was added **2** (1 mol%), then the mixture was stirred at rt. The consumption of the starting material was followed by ^1H NMR spectroscopy. The conversion of epoxides was calculated from the ^1H NMR spectrum of the reaction mixture by comparing the integrals of the signals assigned to epoxides and the products.

iii) To a solution of the corresponding oligoepoxide (1.0 M) and CH_2Br_2 (0.3 M) in CD_2Cl_2 was added AcOH (100 mol%), then the mixture was stirred at the corresponding temperature. The conversion of epoxides was calculated from the ^1H NMR spectrum of the reaction mixture by comparing the integrals of the signals assigned to epoxides and the internal standard (CH_2Br_2).

iv) To a solution of the corresponding oligoepoxide (1.0 M) in CD_2Cl_2 was added $SbCl_3$ (100 mol%), then the mixture was stirred at rt. The reaction mixture was diluted with $CDCl_3$, washed twice with 1 M NaOH aqueous solution, once with water, and dried over Na_2SO_4. The conversion of epoxides was calculated from the ^1H NMR spectrum of the reaction mixture by comparing the integrals of the signals assigned to epoxides and the products.

Table S11 Condition screening for oligo-epoxide

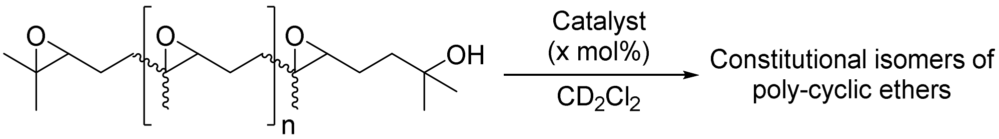

34: n = 1
35: n = 2

Entry		C (mol%)[a]	S[b]	*c* (M)[c]	*T* (°C)[d]	*t* (d)[e]	η_t (%)[f]
1[g]	**1**	$Sb(FP_{345})_3$ (500)	**34**	0.5	60	9	95
2[h]	**2**	$Sb(FP_{345})_3Ch$ (1)	**34**	1.0	rt	1	>95
3[i]	-	AcOH (100)	**34**	1.0	rt	10	90
4[i]	-	AcOH (100)	**34**	1.0	40	5	89
5[j]	-	$SbCl_3$ (100)	**34**	1.0	rt	*vf*[k]	>95
6[g]	**1**	$Sb(FP_{345})_3$ (500)	**35**	0.5	60	9	95
7[h]	**2**	$Sb(FP_{345})_3Ch$ (1)	**35**	1.0	rt	1	>95
8[i]	-	AcOH (100)	**35**	1.0	40	5	80
10[j]	-	$SbCl_3$ (100)	**35**	1.0	rt	*vf*[l]	>95

[a]Catalysts, FP = fluorophenyls, numbers indicate position. In bracket, catalyst concentration in mol%. [b]Substrate used in the reaction. [c]Substrate concentration. [d]Reaction temperature. [e]Reaction time to reach the indicated conversion. [f]Epoxides conversion determined by ^{1}H NMR spectroscopy. [g]Procedure i) was followed. [h]Procedure ii) was followed. [i]Procedure iii) was followed. [j]Procedure iv) was followed. [k]Very fast in day scale (<15 min). [l]Very fast in day scale (<30 min).

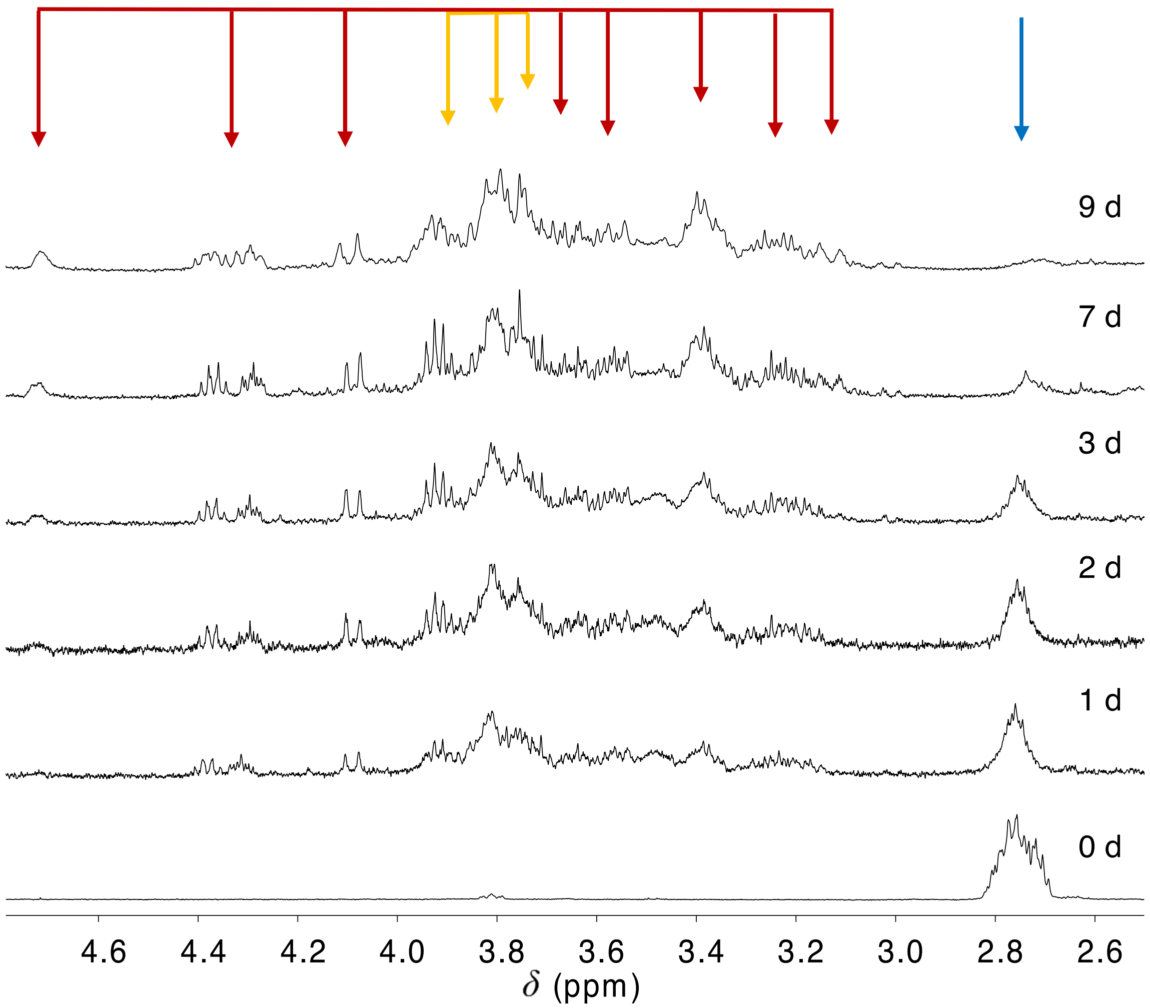

Fig. S14 ^{1}H NMR spectra of a mixture of substrate **34** (0.5 M) and **1** (500 mol%) in CD_2Cl_2 at 60 °C. The blue arrow shows the consumption of epoxides in **34** and the intermediates, the yellow ones the formation of the BBB cyclization product and the red ones the formation of the ABB, AAB, BAB, BBA, BAA, ABA, and/or AAA products.

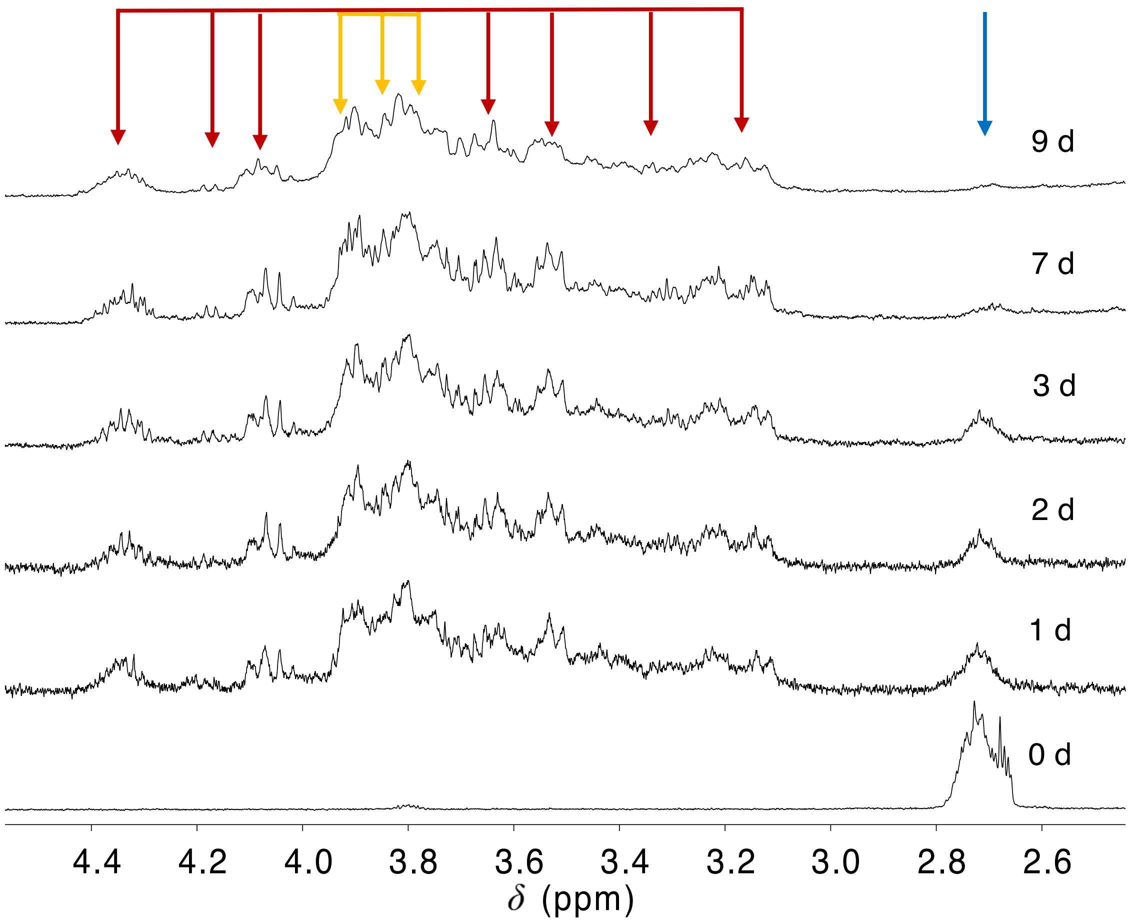

Fig. S15 ^{1}H NMR spectra of a mixture of substrate **35** (0.5 M) and **1** (500 mol%) in CD_2Cl_2 at 60 °C. The blue arrow shows the consumption of epoxides in **35** and the intermediates, the yellow ones the formation of the BBBB cyclization product and the red ones the formation of the ABBB, BABB, BBAB, BBBA, AABB, ABAB, ABBA, BABA, BBAA, BAAB, BAAA, ABAA, AABA, AAAB and/or AAAA products.

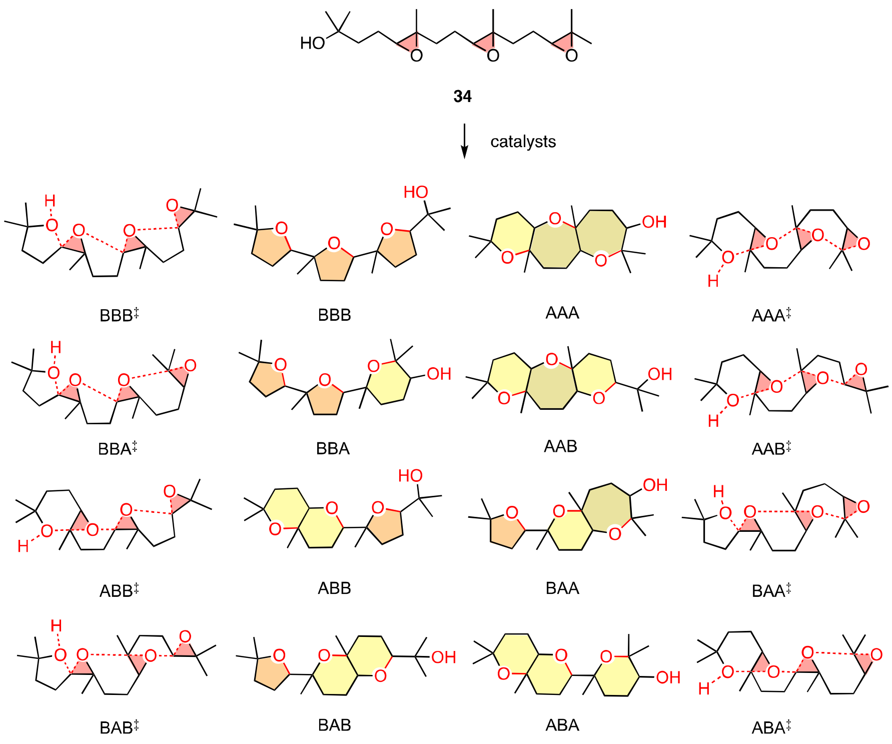

Fig. S16 Constitutional isomers accessible by cyclization of trimer **34**.

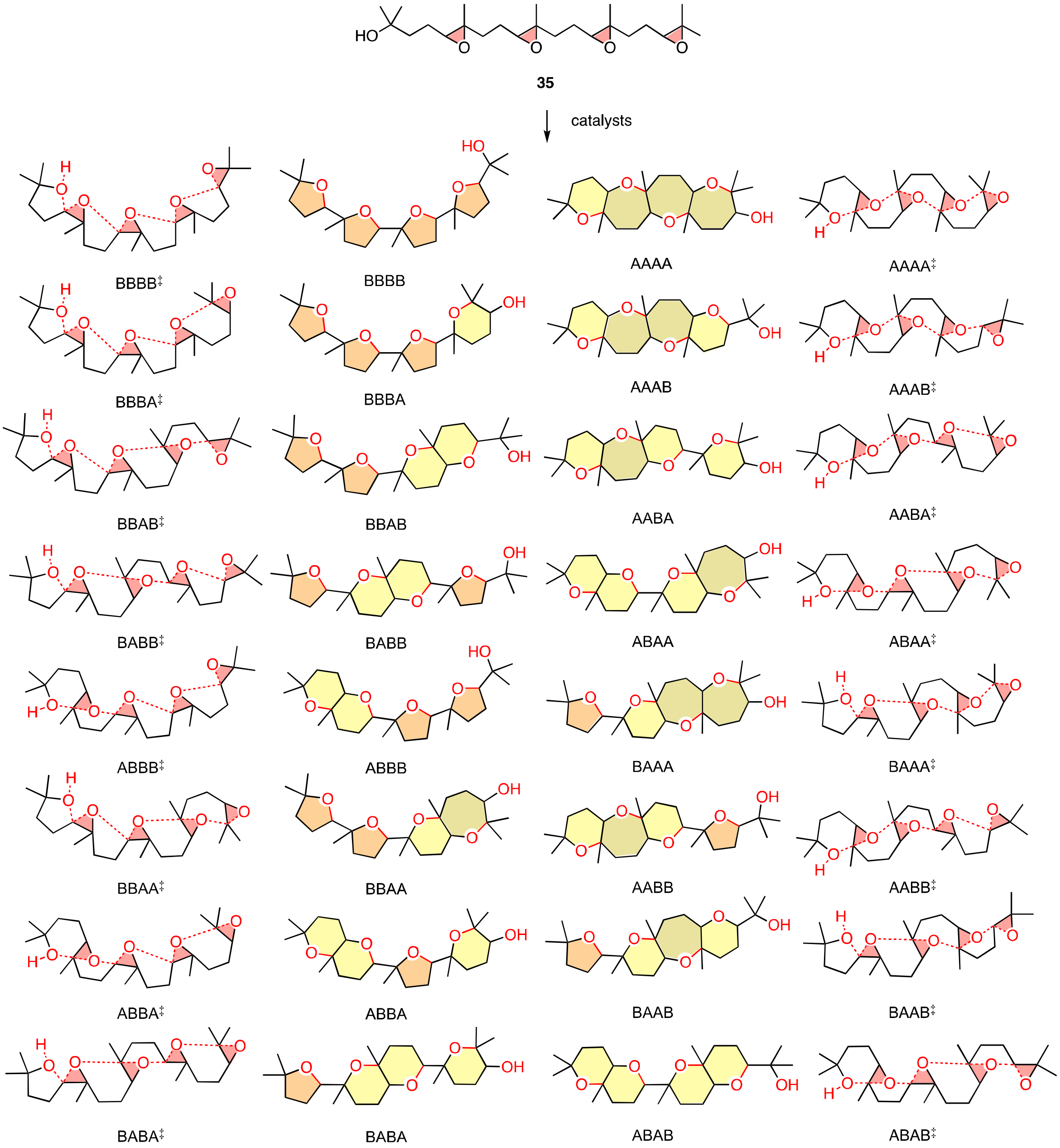

Fig. S17 Constitutional isomers accessible by cyclization of tetramer **35**.

5.2. Comparison with conventional catalysts

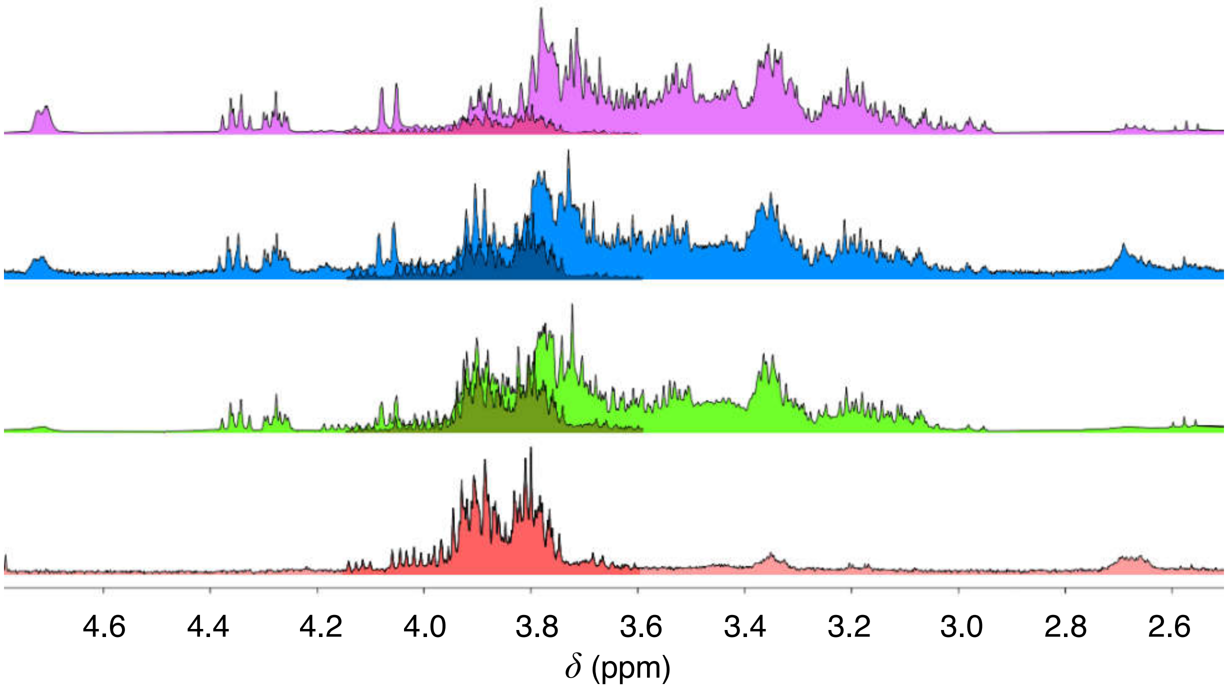

Fig. S18 Comparison of ^{1}H NMR spectra of reaction mixtures from the cyclization of substrate **34** using different catalysts. The darker regions represent the overlaid BBB signature from the reaction mixture of substrate **34** with AcOH (red). In order from top: **2** (1 mol%) at rt (purple), **1** (500 mol%) at 60 °C (cyan), $SbCl_3$ (100 mol%) at rt (green), AcOH (100 mol%) at 40 °C (red).

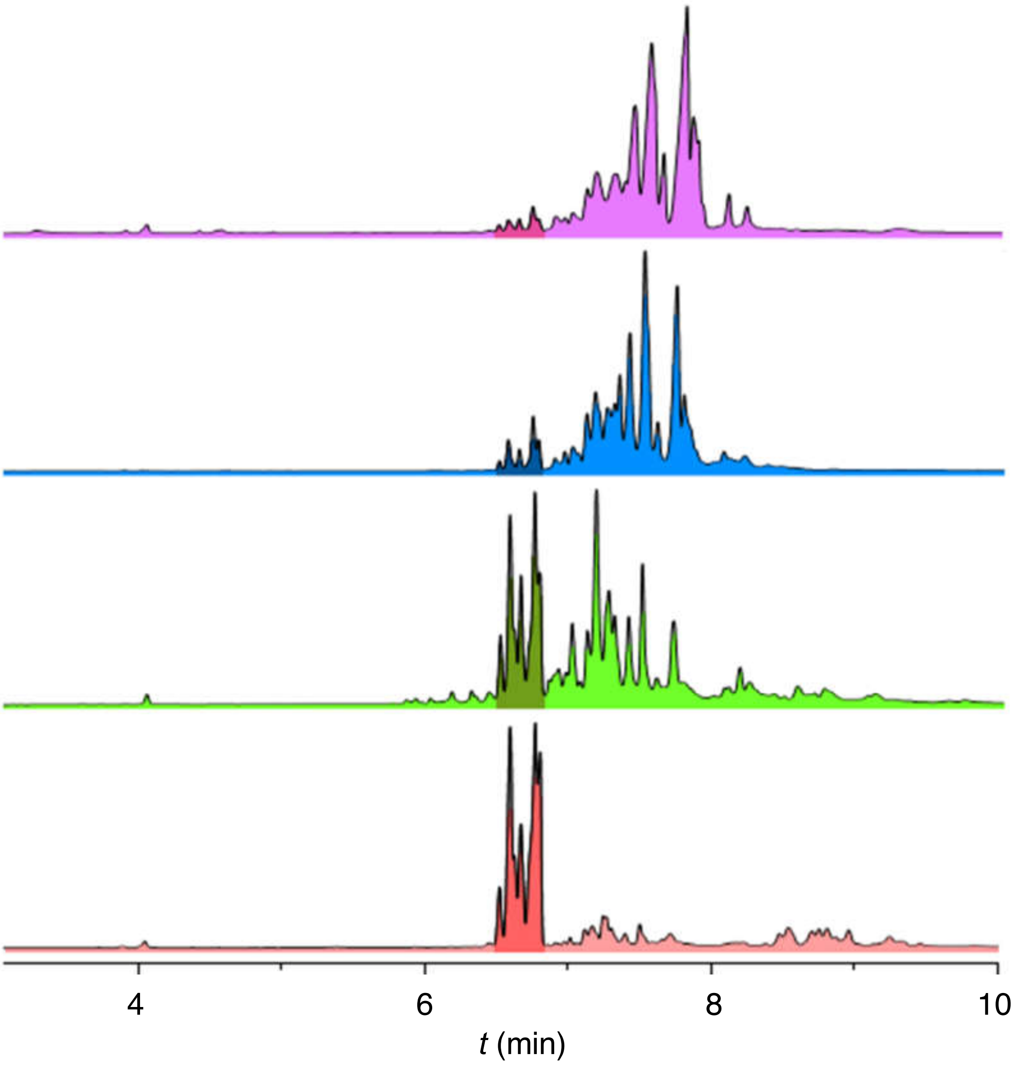

Fig. S19 Comparison of GC-FID chromatograms of reaction mixtures from the cyclization of substrate **34** using different catalysts. The darker regions represent the overlaid BBB signature from the reaction mixture of substrate **34** with AcOH (red). In order from top: **2** (1 mol%) at rt (purple), **1** (500 mol%) at 60 °C (cyan), $SbCl_3$ (100 mol%) at rt (green), AcOH (100 mol%) at 40 °C (red). Method: Inlet 150 °C; Oven 150 °C (2 min), from 150 °C to 250 °C (20 °C/min), 250 °C (5 min).

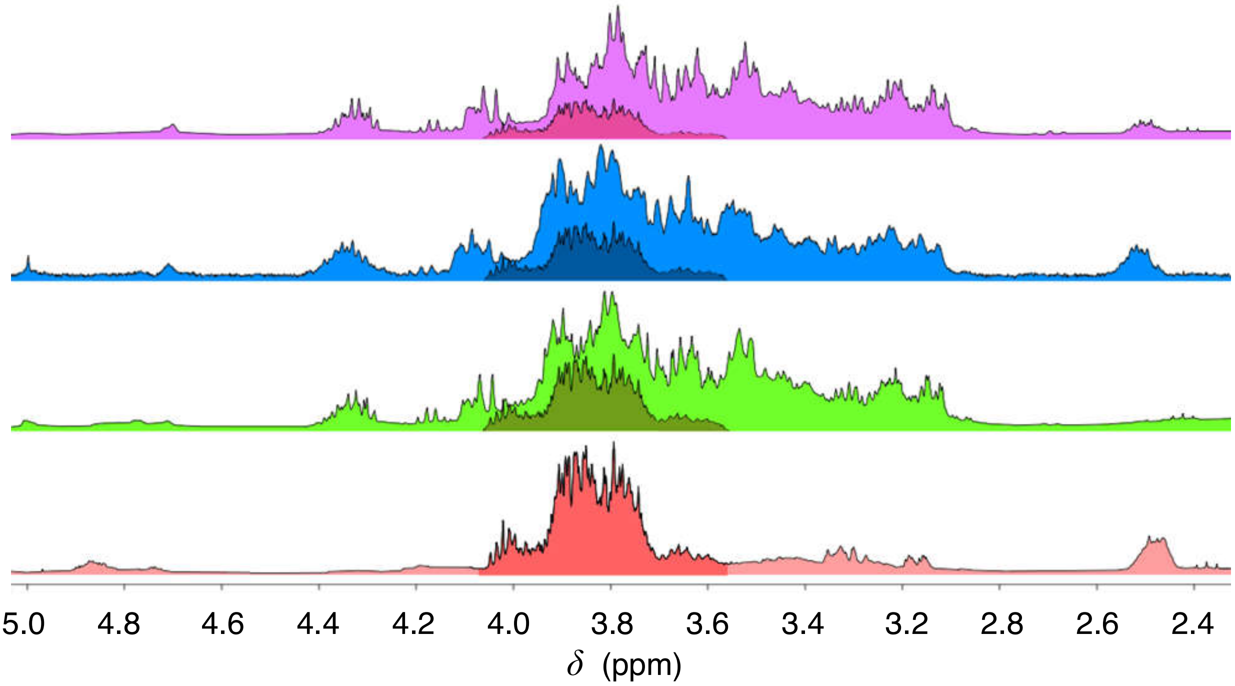

Fig. S20 Comparison of ^{1}H NMR spectra of reaction mixtures from the cyclization of substrate **35** using different catalysts. The darker regions represent the overlap Baldwin region from the reaction mixture of substrate **35** with AcOH (red). In order from top: **2** (1 mol%) at rt (purple), **1** (500 mol%) at 60 °C (cyan), $SbCl_3$ (100 mol%) at rt (green), AcOH (100 mol%) at 40 °C (red).

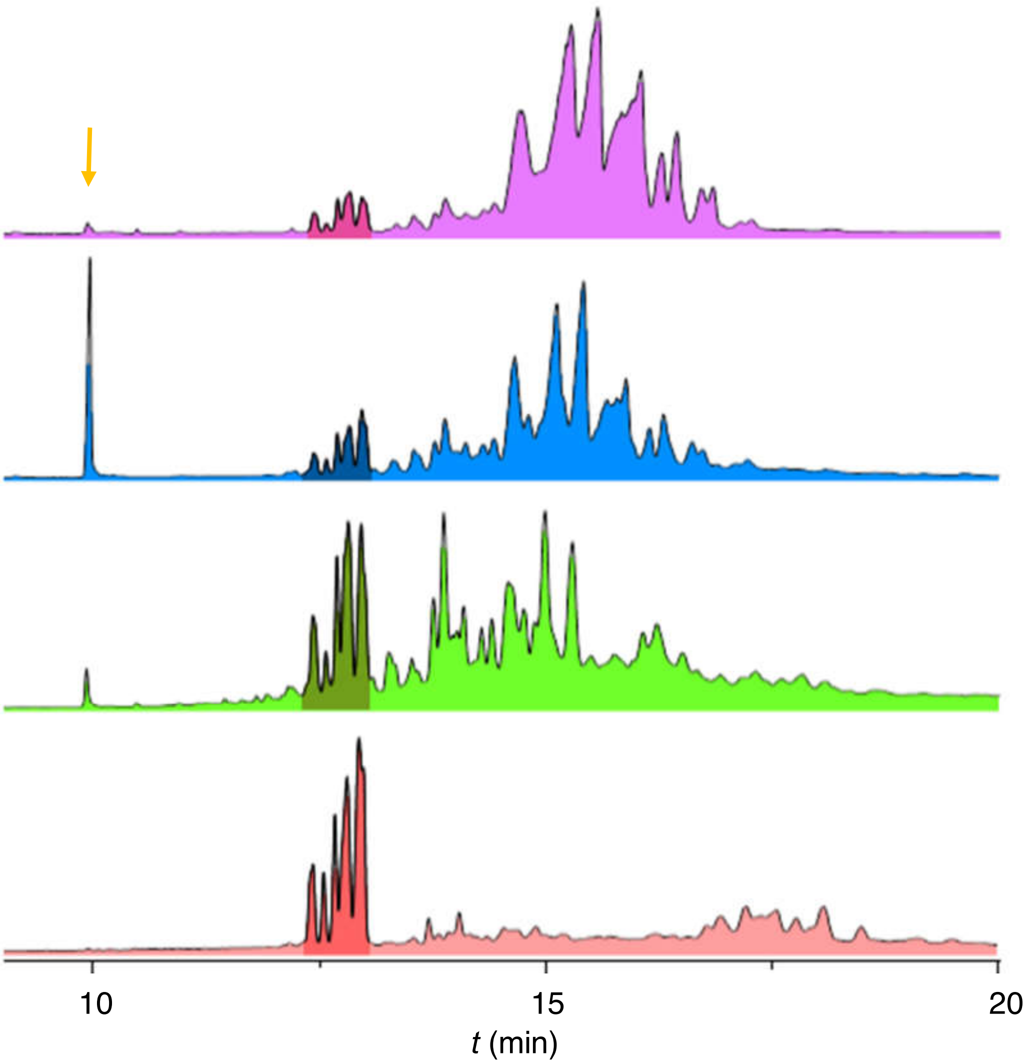

Fig. S21 Comparison of GC-FID chromatograms of reaction mixtures from the cyclization of substrate **35** using different catalysts. The darker regions represent the overlaid of the BBBB signature from the reaction mixture of substrate **35** with AcOH (red). The yellow arrow indicates the not-fully cyclized intermediates. In order from top: **2** (1 mol%) at rt (purple), **1** (500 mol%) at 60 °C (cyan), $SbCl_3$ (100 mol%) at rt (green), AcOH (100 mol%) at 40 °C (red). Method: Inlet 150 °C; Oven 150 °C (2 min), from 150 °C to 250 °C (10 °C/min), 250 °C (5 min).

6. Computational studies

The energies of all complexes included in this study were computed at the BP86-D3/def2-TZVP level of theory. The calculations have been performed by using the program TURBOMOLE version 7.0.[S12] For the calculations the BP86 functional with the latest available correction for dispersion (D3) was employed.[S13] The minimum nature of the compounds has been confirmed by performing frequency calculations. The MEP surfaces have been carried out employing the SPARTAN software.[S14] To reproduce solvent effects, the conductor-like screening model COSMO was used,[S15] which is a variant of the dielectric continuum solvation models.[S16] THF was used as a solvent continuum ($\varepsilon = 7.6$), values for CH_2Cl_2 are given in figure legends ($\varepsilon = 8.9$).

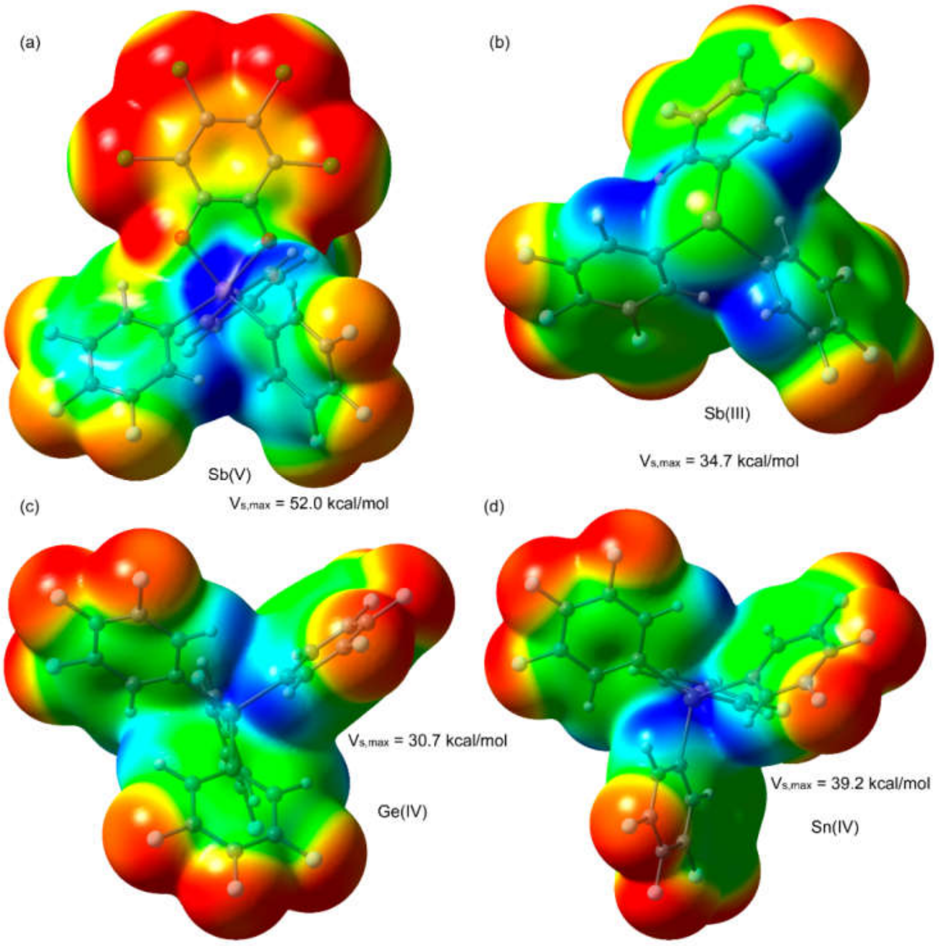

Fig. S22 MEP surfaces (0.001 a.u. isosurface) for (a) **2**, (b) **1**, (c) **4** and (d) **3**. The maximum of MEP values is indicated at the PBE0-D3/def2TZVP.

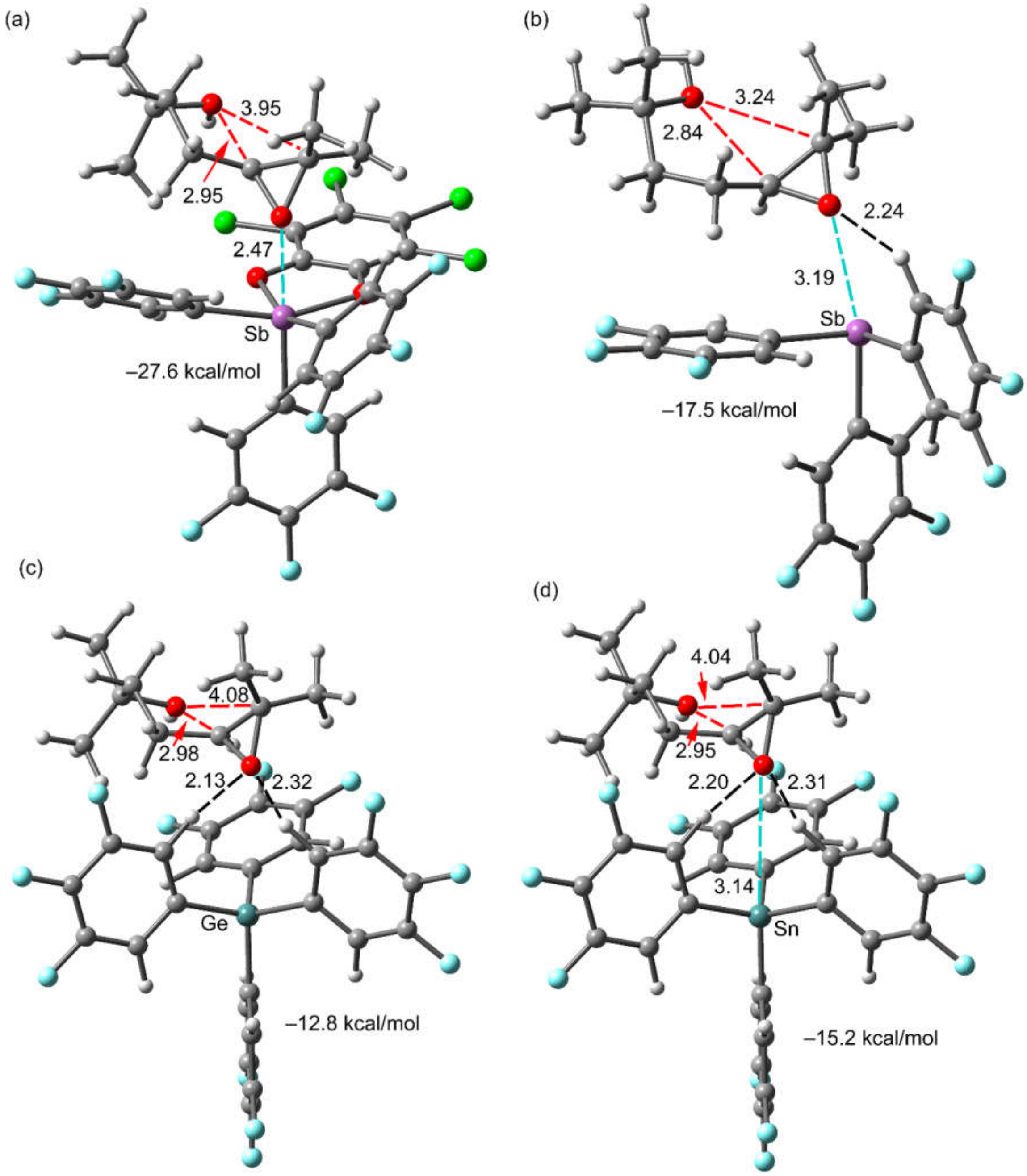

Fig. S23 BP86-D3/def2-TZVP optimized geometries of the complexes between catalysts **1-4** and mono-epoxide **19**. σ-Hole interactions represented as blue dashed lines. Distances in Å. (In CH_2Cl_2 solvent continuum: –22.0, –13.9, –10.7, –12.7 kcal mol^{-1}).

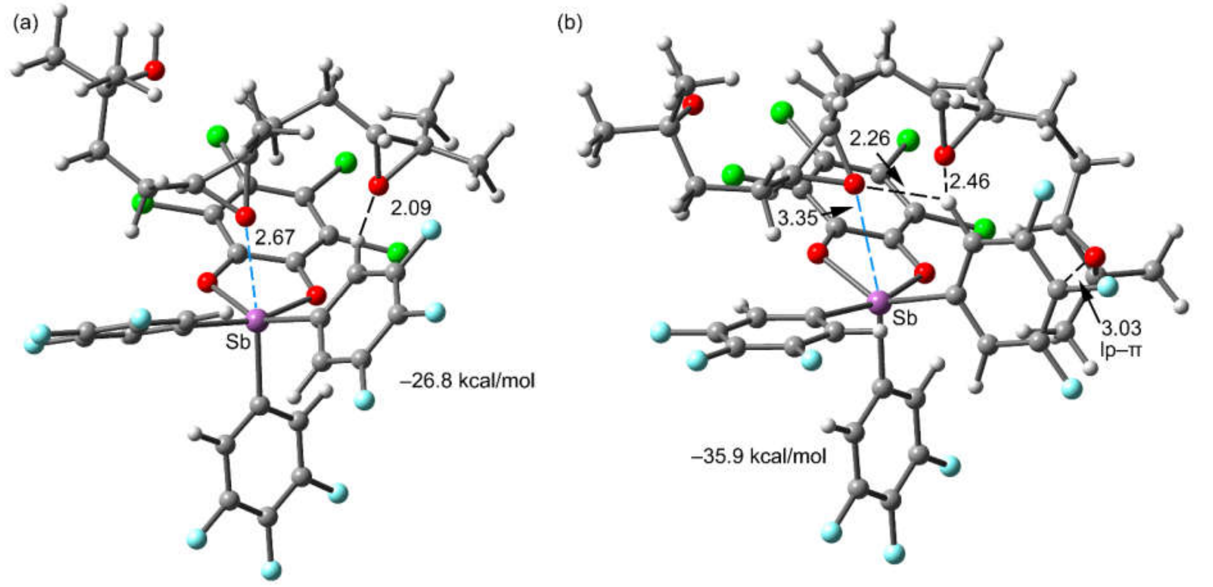

Fig. S24 BP86-D3/def2-TZVP optimized complexes of Sb(V) catalyst **2** with (a) *trans*-di-epoxide **28** and (b) tri-epoxide **34**. σ-Hole interactions represented as blue dashed lines. Distances in Å. (In CH_2Cl_2 solvent continuum: –19.5, –27.7 kcal mol^{-1}).

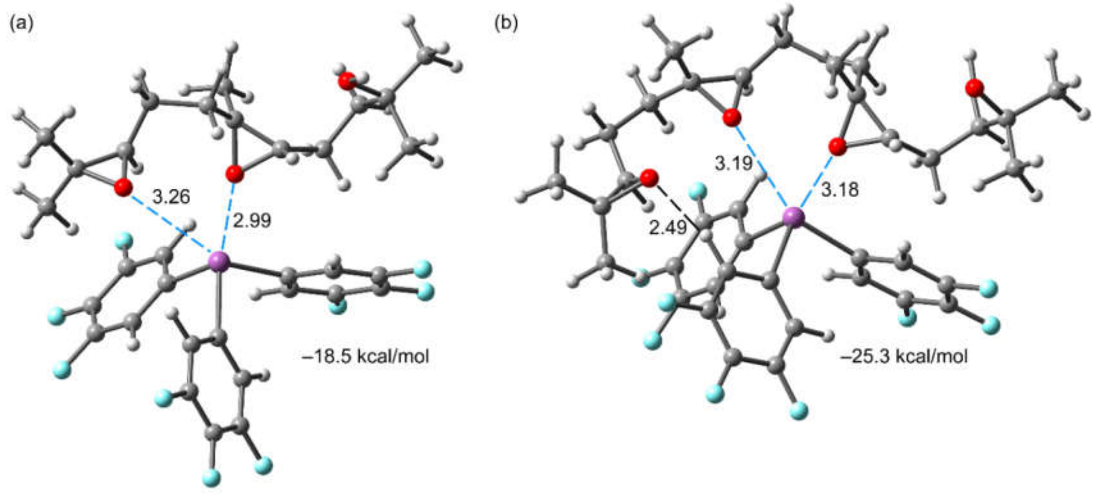

Fig. S25 BP86-D3/def2-TZVP optimized complexes of the Sb(III) catalyst **1** with (a) *trans*-di-epoxide **28** and (b) tri-epoxide **34**. σ-Hole interactions represented as blue dashed lines. Distances in Å. (In CH_2Cl_2 solvent continuum: –13.8, –20.4 kcal mol^{-1}).

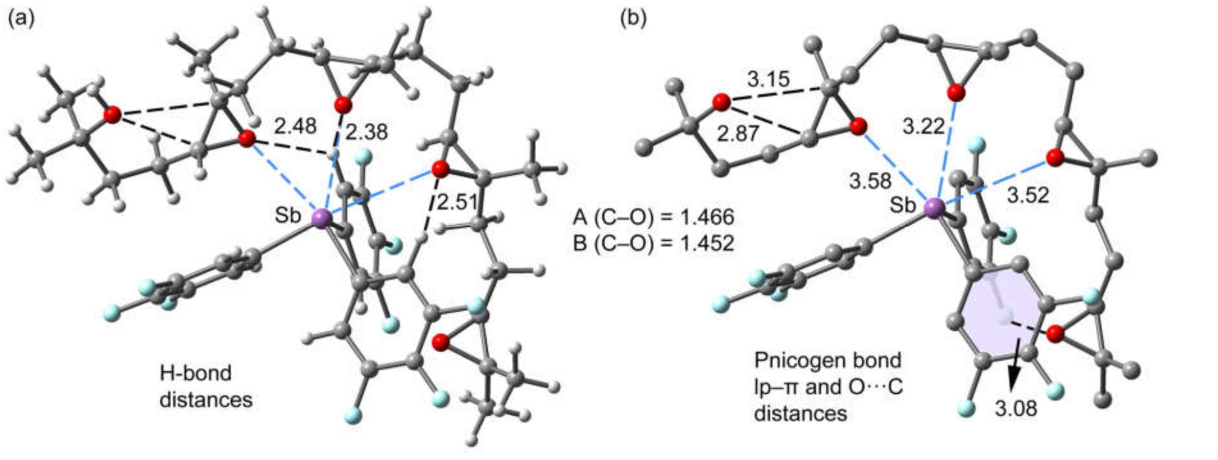

Fig. S26 BP86-D3/def2-TZVP optimized complexes of the Sb(III) catalyst **1** with tetra-epoxide **35** without H-atoms (right) and with H-atoms (left). σ-Hole interactions represented as blue dashed lines. Distances in Å.

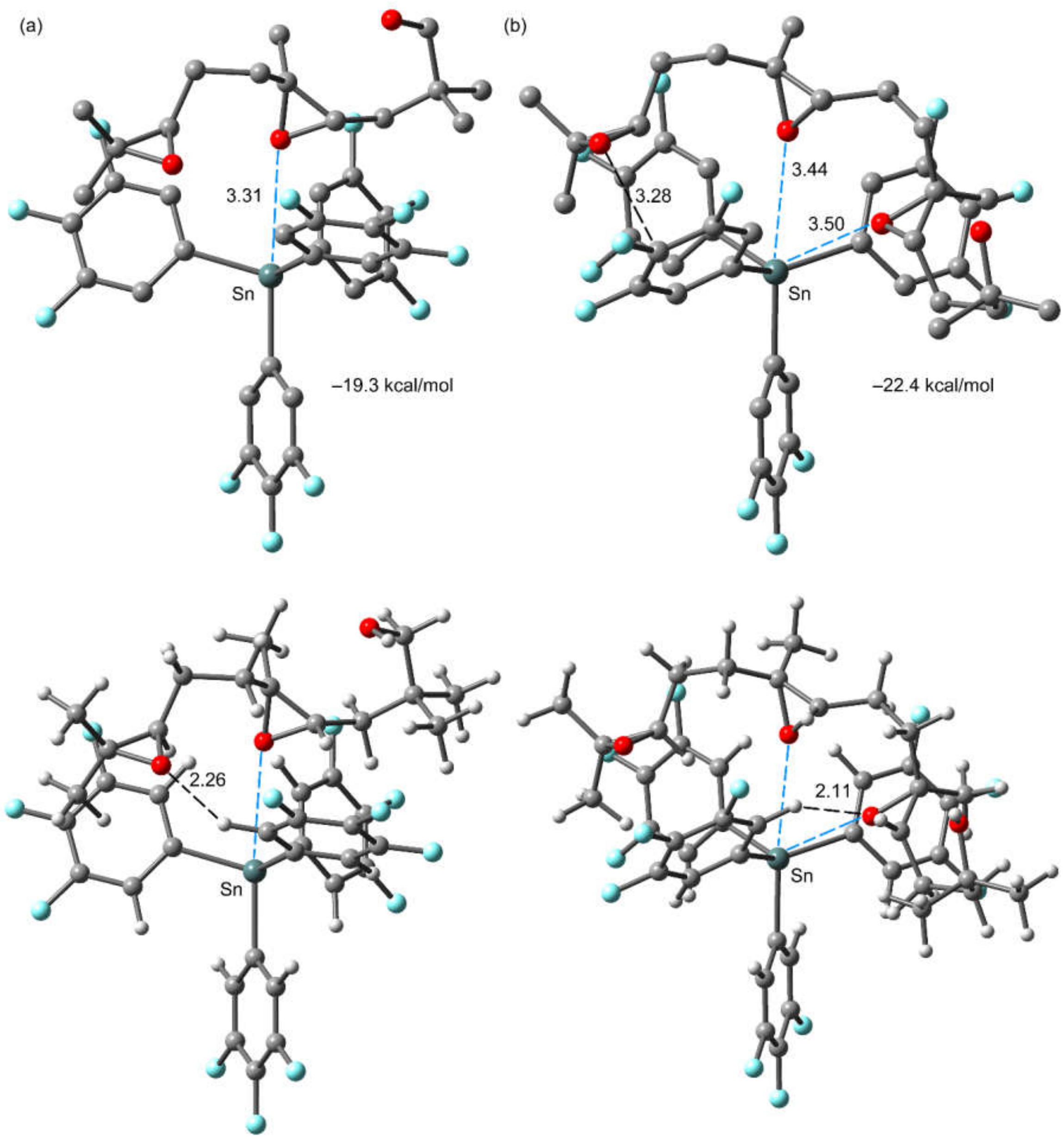

Fig. S27 BP86-D3/def2-TZVP optimized complexes of the Sn(IV) catalyst **3** with (a) *trans*-di-epoxide **28** and (b) tri-epoxide **34** without H-atoms (top) and with H-atoms (bottom). σ-Hole interactions represented as blue dashed lines. Distances in Å. (In CH_2Cl_2 solvent continuum: –14.0, –17.4 kcal mol^{-1}).

The geometries and interaction energies of the catalysts complexed to notional anionic alcoholate intermediates using substrate **28** were also computed. In Fig. S28 the complexes for the Sn catalyst and also their differences in energy was showed (positive values indicate that the anti-Baldwin intermediate is more stable and negative values that the Baldwin intermediate is more stable). Most importantly, both complexes documented structural flexibility deviating from the tetrahedron without ligands. In the anti-Baldwin complex with two tetrel bonds in *cis* position in a hexacoordinated octahedron, and the catalyst opening up into an almost perfect seesaw structure with the known proximal deep σ holes.[S16]

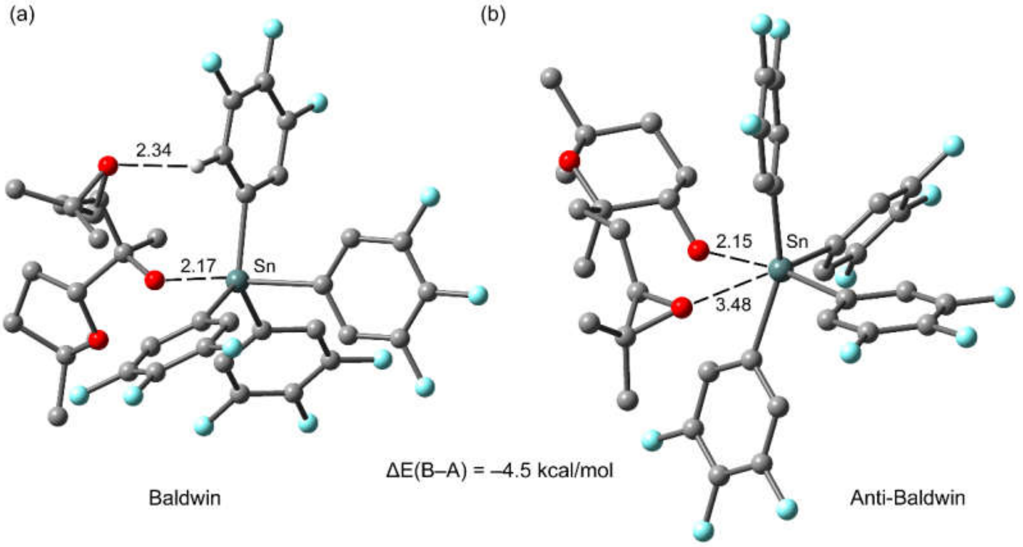

Fig. S28 PB86-D3/def2-TZVP geometries and energies of hypothetic Baldwin (a) and anti-Baldwin (b) anionic alcoholate intermediates from substrate **28** complexed to Sn catalyst **3**. Distances in Å. The energy difference between both catalysts is also given.

Fig. S23a

Sb	1.3707997	-0.1568836	0.7391108
O	0.3594904	-1.5513848	-0.4452336
O	2.4443585	0.0012940	-1.0286316
C	2.7062573	-1.5582536	1.6542712
C	2.3731705	-2.1678546	2.8694374
H	1.4340340	-1.9685182	3.3876914
C	3.2655007	-3.0720065	3.4379754
C	4.4780185	-3.3790492	2.8134705
C	4.7887767	-2.7560524	1.5997202
C	3.9167762	-1.8464756	1.0131000
H	4.1953856	-1.3799450	0.0680111
C	2.2433405	1.7151780	1.3405062
C	2.5586139	1.8918516	2.6916735
H	2.4107781	1.1100215	3.4386111
C	3.0827760	3.1125192	3.1079675
C	3.2989646	4.1517459	2.1980380
C	2.9887331	3.9417300	0.8495502
C	2.4623500	2.7329728	0.4111420
H	2.2458527	2.5992161	-0.6469854
C	-0.3459161	-0.2424719	2.0432346
C	-1.1085770	-1.4148557	2.1076227
H	-0.8453268	-2.3107837	1.5470598
C	-2.2686436	-1.4208575	2.8745605
C	-2.6744379	-0.2866270	3.5858484
C	-1.8895435	0.8685452	3.5132373
C	-0.7330670	0.9039568	2.7430886
H	-0.1767661	1.8388184	2.6832208
C	0.4052149	-2.5561813	-2.6289269
C	0.9106558	-1.6826874	-1.6597363
C	2.0210964	-0.8583216	-1.9709557
C	2.6031267	-0.9198569	-3.2414467
C	2.0969165	-1.8072005	-4.2138257
C	0.9952808	-2.6262117	-3.9070812
Cl	0.3589649	-3.7194268	-5.0887959
Cl	2.8331052	-1.8787857	-5.7782270
Cl	3.9265645	0.1465483	-3.5766229
Cl	-0.9554620	-3.5488074	-2.1997917
F	2.9753645	-3.6753075	4.6078166
F	5.3273438	-4.2559470	3.3655469
F	5.9595230	-3.0595886	1.0083545
F	-2.2886057	1.9593161	4.1965300
F	-3.7948908	-0.3051693	4.3206281
F	-3.0386219	-2.5243926	2.9364173
F	3.3865472	3.3200709	4.4040990
F	3.7952359	5.3260513	2.6113508
F	3.2066020	4.9463571	-0.0225697
C	-0.5949748	2.1033762	-1.6110669

C	-1.4068201	0.9906540	-1.0650310
H	-1.2768198	0.0344074	-1.5743812
O	-0.1892280	1.4096164	-0.3526153
C	0.2702050	1.8427048	-2.8161096
H	-0.2338943	2.2350707	-3.7108029
H	0.4447313	0.7720278	-2.9666271
C	-2.7050518	1.1139532	-0.3135771
H	-2.6423891	0.4646930	0.5726305
H	-2.8335214	2.1366920	0.0677989
C	-3.9278757	0.7218734	-1.1605452
C	-0.9493049	3.5480903	-1.3694318
H	-0.0349303	4.1573831	-1.3387922
H	-1.5764548	3.9202543	-2.1922982
H	-1.4867057	3.6856927	-0.4249231
C	-3.9822698	-0.7346015	-1.6687833
H	1.2421036	2.3485291	-2.7283331
H	-4.8294446	0.9034897	-0.5560495
H	-4.0007596	1.3781267	-2.0422764
C	-5.3792625	-1.0268502	-2.2329054
H	-5.3990233	-2.0271761	-2.6890220
H	-6.1469087	-0.9947835	-1.4454050
H	-5.6361599	-0.2923745	-3.0082682
C	-3.6202027	-1.7444201	-0.5739022
H	-2.5641251	-1.6580625	-0.2881735
H	-4.2426980	-1.6009708	0.3211411
H	-3.7838244	-2.7713658	-0.9326986
O	-3.0213078	-0.8072239	-2.7553459
H	-2.7987438	-1.7450955	-2.9042679

Fig. S23b

Sb	-0.9592169	0.6296280	-2.0126524
C	0.0454919	-1.0476562	-1.0527150
C	1.4335676	-1.2153638	-1.1144758
C	-0.7353809	-1.9693565	-0.3416105
C	2.0260770	-2.2944771	-0.4655228
H	2.0777533	-0.5175130	-1.6498536
C	-0.1241052	-3.0393527	0.3025270
H	-1.8201276	-1.8799059	-0.2627104
C	1.2612571	-3.2173733	0.2534563
C	0.8657548	1.4814741	-2.8420598
C	1.2206867	1.2801506	-4.1824509
C	1.6916224	2.2488930	-2.0063760
C	2.3973893	1.8371396	-4.6728010
H	0.6114246	0.6868533	-4.8644999
C	2.8635301	2.7933283	-2.5197440
H	1.4587998	2.4081508	-0.9529554
C	3.2357595	2.5969422	-3.8532311
C	-1.4225039	-0.5190294	-3.8138944
C	-2.4294087	-0.0447912	-4.6660911

C	-0.7235850	-1.6863413	-4.1500049
C	-2.7254125	-0.7346656	-5.8381372
H	-3.0022265	0.8585060	-4.4481176
C	-1.0353050	-2.3630770	-5.3233780
H	0.0708120	-2.0872004	-3.5190634
C	-2.0382078	-1.9016374	-6.1817813
C	-0.4264828	2.1960295	1.9449438
C	-0.3180403	0.7384021	1.7673491
H	-1.2354419	0.2375148	1.4294487
O	0.2424834	1.6328451	0.7630782
C	-1.7513107	2.8610362	1.6579885
H	-2.3361412	2.9388492	2.5857015
H	-2.3299426	2.2809924	0.9278708
C	0.6446883	-0.1932951	2.4596675
H	1.1780268	-0.7419153	1.6680808
H	1.4141809	0.3686055	3.0061409
C	-0.0473085	-1.2184243	3.3680000
F	-0.8600722	-3.9242871	1.0095321
F	1.8449633	-4.2432396	0.8934880
F	3.3632402	-2.4682892	-0.5081284
F	-0.3698453	-3.4867454	-5.6630112
F	-2.3371797	-2.5679741	-7.3076434
F	-3.6908068	-0.2900958	-6.6701021
F	3.6769198	3.5292662	-1.7323406
F	4.3729677	3.1241319	-4.3340919
F	2.7574633	1.6478624	-5.9601329
C	0.4797118	2.9524205	2.8837697
H	0.6487438	3.9723888	2.5087600
H	0.0091968	3.0260875	3.8749244
H	1.4554326	2.4643738	2.9899636
C	-0.7164378	-0.6774482	4.6432012
H	-1.5999814	3.8748329	1.2584424
H	-0.8188392	-1.7487033	2.7865855
H	0.6885477	-1.9785471	3.6701022
C	0.2779147	0.0515967	5.5552061
H	1.1017115	-0.6119542	5.8558785
H	0.7001988	0.9285834	5.0496563
H	-0.2258734	0.3978028	6.4708756
C	-1.3910490	-1.8310250	5.3969094
H	-1.9123318	-1.4552033	6.2912387
H	-2.1270596	-2.3287852	4.7510633
H	-0.6538886	-2.5764329	5.7281506
O	-1.7318806	0.2550134	4.1868321
H	-2.2209242	0.5604050	4.9707442

Fig. S23c

C	-1.0792824	0.4407949	1.0298150
C	-1.0039614	1.8362121	1.1157851
C	-0.4430619	-0.3541061	1.9952247

C	-0.2941190	2.4242238	2.1557856
H	-1.4627725	2.4820918	0.3668553
C	0.2485711	0.2553538	3.0352319
H	-0.4666453	-1.4441998	1.9543172
C	0.3377553	1.6452880	3.1280838
C	-1.1153404	-1.9385706	-1.1219605
C	-1.6229311	-3.2297762	-0.9108851
C	0.1237888	-1.7762560	-1.7599127
C	-0.8934161	-4.3369961	-1.3306540
H	-2.5806684	-3.4025091	-0.4185999
C	0.8373985	-2.8967311	-2.1640443
H	0.5715589	-0.7941313	-1.9171743
C	0.3474773	-4.1895842	-1.9558945
C	-3.7958131	-1.0718333	0.3134575
C	-4.7562773	-1.6091164	-0.5586143
C	-4.0527891	-1.0374331	1.6901766
C	-5.9521655	-2.1026769	-0.0510858
H	-4.6001812	-1.6504010	-1.6376567
C	-5.2555752	-1.5372807	2.1801989
H	-3.3350043	-0.6233221	2.3995468
C	-6.2188478	-2.0748579	1.3218171
C	-2.5659342	0.9944938	-1.7354992
C	-3.8296895	1.5977712	-1.6380388
C	-1.6477452	1.4608015	-2.6870495

C	-4.1613763	2.6529641	-2.4802754
H	-4.5739475	1.2670997	-0.9128467
C	-1.9995300	2.5171699	-3.5180010
H	-0.6439455	1.0501536	-2.7768403
C	-3.2537624	3.1297113	-3.4290616
C	2.2902761	2.0236150	-1.2636028
C	2.1220705	0.9728893	-0.2351333
H	1.8085276	1.3134464	0.7547533
O	1.1022878	1.1693088	-1.2577464
C	2.0039053	3.4594231	-0.8880240
H	2.9366338	3.9807995	-0.6277264
H	1.3256184	3.5129945	-0.0278214
C	2.8463375	-0.3503844	-0.2301504
H	2.1761482	-1.1086772	0.2043704
H	3.0266025	-0.6735810	-1.2643321
C	4.1858173	-0.3074570	0.5280504
F	-0.1828360	3.7669316	2.2376412
F	1.0400353	2.2167267	4.1203454
F	0.8654168	-0.4846593	3.9835587
F	-5.5203152	-1.5115582	3.5021562
F	-7.3758985	-2.5518056	1.8028566
F	-6.8846460	-2.6196946	-0.8756044
F	-5.3708623	3.2442387	-2.3907889
F	-3.5754890	4.1558035	-4.2314596

F	-1.1226564	2.9896001	-4.4292340
F	2.0429217	-2.7581787	-2.7593501
F	1.0548387	-5.2631249	-2.3403644
F	-1.3693891	-5.5840577	-1.1330411
C	3.2010775	1.8421362	-2.4552968
H	2.7842217	2.3652296	-3.3284415
H	4.1916301	2.2728105	-2.2464590
H	3.3270171	0.7863694	-2.7190587
C	4.1195680	-0.3883582	2.0641172
H	1.5415707	3.9896958	-1.7338400
H	4.7222192	0.6164683	0.2572326
H	4.8133030	-1.1450212	0.1875099
C	5.5326470	-0.2514854	2.6466475
H	5.4990450	-0.2791247	3.7463349
H	6.1865370	-1.0692064	2.3098890
H	5.9773353	0.7050163	2.3398286
C	3.4714522	-1.6938282	2.5407176
H	4.0330519	-2.5672768	2.1799092
H	3.4526724	-1.7322917	3.6396378
H	2.4355622	-1.7723860	2.1888361
O	3.3197208	0.7444402	2.4889067
H	3.2086703	0.6906758	3.4547865
Ge	-2.1044140	-0.3908099	-0.4228119

Fig. S23d

Sn	-1.9395562	-0.3763591	-0.4648083
C	-0.9456212	0.3133242	1.3110184
C	-0.8229828	1.6877981	1.5516239
C	-0.3799796	-0.6042614	2.2060811
C	-0.1107625	2.1325746	2.6606095
H	-1.2394639	2.4327232	0.8719320
C	0.3078726	-0.1391073	3.3219759
H	-0.4478870	-1.6833677	2.0584785
C	0.4684800	1.2270627	3.5528247
C	-1.0319191	-2.1468244	-1.2915251
C	-1.6885150	-3.3681705	-1.0733024
C	0.1649752	-2.1217116	-2.0219558
C	-1.1445377	-4.5442170	-1.5803549
H	-2.6253658	-3.4348835	-0.5179313
C	0.6857627	-3.3083490	-2.5231409
H	0.7141182	-1.1953234	-2.1882520
C	0.0457242	-4.5330657	-2.3118494
C	-3.8823527	-1.0460621	0.2125688
C	-4.8269448	-1.5374412	-0.7023409
C	-4.1982661	-1.0106457	1.5781974
C	-6.0621425	-1.9880141	-0.2483331
H	-4.6317105	-1.5844880	-1.7750808
C	-5.4399196	-1.4640996	2.0139375

H	-3.4960743	-0.6350021	2.3247072
C	-6.3855989	-1.9592906	1.1119186
C	-2.3611886	1.2611981	-1.7931591
C	-3.6125646	1.8818067	-1.6599536
C	-1.4446248	1.7436638	-2.7367768
C	-3.9293869	2.9780313	-2.4559328
H	-4.3623570	1.5316358	-0.9485541
C	-1.7805895	2.8409811	-3.5197741
H	-0.4615010	1.2964529	-2.8697466
C	-3.0201992	3.4754161	-3.3928368
C	1.9987133	1.7060897	-1.3073586
C	2.0310186	0.7709413	-0.1606087
H	1.7365871	1.1903613	0.8043077
O	0.9338096	0.7136561	-1.1160400
C	1.5560857	3.1312340	-1.0702221
H	2.4312265	3.7846285	-0.9423757
H	0.9355000	3.2025008	-0.1691497
C	2.9152056	-0.4451616	-0.0558409
H	2.3689214	-1.2199173	0.5042014
H	3.0849195	-0.8641492	-1.0579715
C	4.2743431	-0.1454660	0.6038095
F	0.0583054	3.4524108	2.8844012
F	1.1912678	1.6600018	4.6031025
F	0.8568843	-1.0048700	4.2011462
F	-5.7596580	-1.4379160	3.3246205
F	-7.5780855	-2.3987653	1.5419592
F	-6.9780926	-2.4679264	-1.1143493
F	-5.1265124	3.5882697	-2.3348422
F	-3.3289470	4.5382831	-4.1512810
F	-0.9005197	3.3293200	-4.4219581
F	1.8381690	-3.3026285	-3.2300503
F	0.5614277	-5.6726732	-2.7985403
F	-1.7590739	-5.7280614	-1.3753864
C	2.8403656	1.4938206	-2.5433514
H	2.3027116	1.8585478	-3.4313907
H	3.7788106	2.0618960	-2.4658829
H	3.0832289	0.4369429	-2.6998123
C	4.2947772	0.0232578	2.1354770
H	0.9762626	3.5019013	-1.9283337
H	4.6936783	0.7698279	0.1554733
H	4.9719575	-0.9610128	0.3607448
C	5.7141780	0.3895757	2.5904632
H	5.7396343	0.5391683	3.6806365
H	6.4327389	-0.4055064	2.3441724
H	6.0392086	1.3210515	2.1072182
C	3.8128498	-1.2401438	2.8572202
H	4.4593644	-2.0985088	2.6253046
H	3.8289984	-1.0882669	3.9465652

H	2.7832550	-1.4896636	2.5752615
O	3.4031869	1.1291191	2.4314117
H	3.3883768	1.2558470	3.3969841

Fig. S24a

Sb	1.1591730	-1.1613542	1.4228130
O	0.1002553	-2.3850518	0.1267544
O	2.4547490	-1.2267722	-0.2004301
C	2.3394376	-2.5338513	2.5667130
C	1.9140533	-2.9145446	3.8456457
H	0.9654110	-2.5845847	4.2695845
C	2.7230012	-3.7512638	4.6072192
C	3.9449020	-4.2168355	4.1142981
C	4.3463117	-3.8277207	2.8310471
C	3.5600519	-2.9870972	2.0517929
H	3.9093000	-2.6959837	1.0612728
C	2.0036566	0.7417193	1.9487918
C	2.2750679	0.9930408	3.2979543
H	2.1191154	0.2499192	4.0817346
C	2.7752977	2.2407700	3.6592146
C	2.9948424	3.2328881	2.6993757
C	2.7221986	2.9425004	1.3581449
C	2.2361637	1.7030280	0.9647892
H	2.0905925	1.4969402	-0.0977375
C	-0.6123658	-1.1673329	2.6606232
C	-1.3979802	-2.3256847	2.7144752
H	-1.1589895	-3.2168294	2.1357387
C	-2.5445186	-2.3251444	3.5007855
C	-2.9179539	-1.1946295	4.2370058
C	-2.1154018	-0.0523745	4.1684516
C	-0.9671779	-0.0262958	3.3842289
H	-0.3933162	0.8986627	3.3335333
C	-0.0605311	-2.8929179	-2.2195671
C	0.6272283	-2.3927248	-1.1086505
C	1.9063943	-1.8083152	-1.2744786
C	2.5365585	-1.8486388	-2.5227564
C	1.8508767	-2.3687214	-3.6383213
C	0.5442434	-2.8715768	-3.4926384
Cl	-0.3234724	-3.4721759	-4.8666544
Cl	2.6249414	-2.3838317	-5.1893088
Cl	4.1719260	-1.2918759	-2.6286678
Cl	-1.6631895	-3.5145334	-1.9770041
F	2.3409394	-4.1310133	5.8429178
F	4.7178972	-5.0203368	4.8575812
F	5.5242515	-4.2860334	2.3676493
F	-2.4870244	1.0359400	4.8716460
F	-4.0273194	-1.2056033	4.9893834
F	-3.3300327	-3.4172805	3.5649192
F	3.0520690	2.5195708	4.9488471

F	3.4623237	4.4375049	3.0597891
F	2.9409202	3.9119985	0.4413020
C	1.4784544	2.3104319	-2.6218246
C	2.8075344	2.0537179	-3.2293482
C	-1.1141097	1.1639205	-1.0807018
C	-1.7965813	0.0636978	-0.3820170
H	-1.7610300	-0.9031095	-0.8968956
H	1.4360885	3.1377161	-1.8983061
O	-0.4715349	0.4803581	0.0862149
O	2.2985095	1.2117592	-2.1542541
C	0.1531570	1.9279248	-3.2439007
H	-0.4687066	2.8321904	-3.3326127
H	0.3074980	1.5603297	-4.2681291
C	-0.5905473	0.8300205	-2.4694059
H	-1.4748334	0.5273565	-3.0519923
H	0.0548849	-0.0562469	-2.3905063
C	-2.9759839	0.1842362	0.5488426
H	-2.8029043	-0.5002578	1.3892698
H	-3.0456581	1.1905270	0.9829131
C	2.9314141	1.3700814	-4.5709517
H	2.8550663	2.1065509	-5.3850085
H	3.9045283	0.8681390	-4.6492803
H	2.1602556	0.6038460	-4.7094288
C	3.9834091	2.9139716	-2.8291905
H	4.8919770	2.2964645	-2.7732751
H	4.1551775	3.7044243	-3.5744590
H	3.8180271	3.3794249	-1.8496749
C	-1.4589112	2.5986524	-0.7866275
H	-0.5740304	3.2417683	-0.8804310
H	-2.2210415	2.9403801	-1.5002964
H	-1.8499312	2.7143626	0.2299420
C	-4.2935972	-0.2427167	-0.1203133
H	-4.1381917	-1.2138170	-0.6185605
H	-5.0451819	-0.4116474	0.6655316
C	-4.9142002	0.7200635	-1.1474062
C	-5.2501397	2.0841782	-0.5317167
H	-5.7114821	2.7396124	-1.2859628
H	-5.9590456	1.9794683	0.3023554
H	-4.3457749	2.5815553	-0.1609154
C	-6.1716862	0.0802111	-1.7493177
H	-6.6201630	0.7445530	-2.5049602
H	-5.9182560	-0.8731720	-2.2324604
H	-6.9327414	-0.1066756	-0.9783782
O	-3.9197771	0.8906917	-2.1917768
H	-4.3448180	1.3695039	-2.9245911

Fig. S24b

Sb	0.5002639	-1.1970355	1.2264710
O	-0.7165656	-1.9162876	-0.2627770

O	1.8803220	-1.4598748	-0.3308831
C	1.4625077	-2.7722662	2.3157144
C	0.9275574	-3.1954285	3.5389269
H	0.0053400	-2.7832154	3.9483516
C	1.5927975	-4.1822656	4.2593262
C	2.7800460	-4.7491346	3.7842635
C	3.2957153	-4.3062153	2.5612724
C	2.6499097	-3.3250063	1.8194459
H	3.0786993	-3.0065499	0.8703512
C	1.5384731	0.5806601	1.8079175
C	2.4596441	0.4536701	2.8534087
H	2.6501384	-0.4903051	3.3650081
C	3.1876112	1.5746676	3.2387894
C	3.0080544	2.8041747	2.6036640
C	2.0776486	2.8942667	1.5645300
C	1.3331021	1.7970270	1.1562935
H	0.6259647	1.9006962	0.3383857
C	-1.2962642	-1.0544942	2.4158851
C	-2.3598501	-1.9426681	2.2174803
H	-2.3715112	-2.6584214	1.3970308
C	-3.4467594	-1.8898477	3.0818539
C	-3.4908799	-0.9751946	4.1396026
C	-2.4149815	-0.1000849	4.3191564
C	-1.3162105	-0.1296610	3.4644962

H	-0.5068654	0.5838458	3.6279643
C	-0.7459666	-2.5655295	-2.5759669
C	-0.0726227	-2.1603443	-1.4226012
C	1.3209914	-1.9295474	-1.4487519
C	2.0383118	-2.1640017	-2.6247025
C	1.3652654	-2.5697673	-3.7944408
C	-0.0283878	-2.7612952	-3.7749311
Cl	-0.8712675	-3.2373138	-5.2112546
Cl	2.2608672	-2.8075960	-5.2573651
Cl	3.7550408	-1.9168415	-2.5925942
Cl	-2.4636666	-2.7831323	-2.4835318
F	1.1038292	-4.6147470	5.4373040
F	3.4097643	-5.7000020	4.4858324
F	4.4414795	-4.8533490	2.1149480
F	-2.4715185	0.7852265	5.3325972
F	-4.5533815	-0.9296577	4.9555274
F	-4.4955650	-2.7189506	2.9120598
F	4.1037386	1.4808333	4.2227488
F	3.7325040	3.8696379	2.9635203
F	1.9273272	4.0819895	0.9423430
C	0.3746986	2.0303560	-2.5079768
C	1.7482479	1.6827150	-2.9467783
C	-2.3300769	1.5447944	-1.0833704
C	-2.8537365	0.6544370	-0.0352425

H	-2.8199668	-0.4162462	-0.2708111
H	0.2925139	2.9061982	-1.8470833
O	-1.5227696	1.2317343	0.1131301
O	1.0813547	0.9345683	-1.8819235
C	-0.8982733	1.6845492	-3.2449316
H	-1.3585825	2.6200824	-3.6011599
H	-0.6769319	1.0860649	-4.1390883
C	-1.8785069	0.8873682	-2.3722449
H	-2.7851616	0.6517249	-2.9513681
H	-1.4130762	-0.0712334	-2.1138518
C	-3.8847280	1.0192172	1.0048286
H	-3.4495423	0.7874035	1.9876342
H	-4.0767245	2.1005618	1.0158045
C	-5.1919211	0.2283875	0.8599551
C	1.9778704	0.9044368	-4.2213159
H	2.1116348	1.5916481	-5.0698255
H	2.8788022	0.2829862	-4.1430244
H	1.1422701	0.2303441	-4.4374744
C	2.8959835	2.5696325	-2.4862815
H	3.0711307	3.3157980	-3.2786544
H	2.5820921	3.1269329	-1.5895792
C	-2.7648110	2.9858824	-1.1812613
H	-1.9375186	3.6238085	-1.5227063
H	-3.5843869	3.0764629	-1.9092921
H	-3.1030265	3.3749729	-0.2149152
C	-6.0619131	0.5434388	-0.3697919
C	4.2136886	1.8321856	-2.1847435
H	4.4725002	1.1554459	-3.0141951
H	5.0257398	2.5686343	-2.1099584
C	4.1560461	1.0369756	-0.9016397
C	5.3389779	0.5148014	-0.1893791
H	3.2211614	0.4898902	-0.7450277
O	4.6422474	1.6970562	0.3032768
C	5.1739026	-0.7086650	0.6844393
H	5.4429353	-1.6191134	0.1288357
H	5.8227878	-0.6408849	1.5706915
H	4.1333699	-0.8015622	1.0138350
C	6.7443847	0.7514185	-0.6936676
H	7.4421450	0.8186395	0.1545151
H	7.0663808	-0.0875702	-1.3287229
H	6.8219272	1.6778873	-1.2729905
H	-4.9587362	-0.8482042	0.8332873
H	-5.8074564	0.3920975	1.7575857
C	-6.4751235	2.0193561	-0.4252699
H	-5.5950588	2.6674434	-0.5155653
H	-7.1217997	2.2016630	-1.2970195
H	-7.0356374	2.3104827	0.4750931
C	-7.3009682	-0.3612722	-0.3647209

H	-6.9999581	-1.4175804	-0.3557277
H	-7.9329943	-0.1676616	0.5136678
H	-7.9125836	-0.1823620	-1.2625579
O	-5.2399237	0.2246069	-1.5221970
H	-5.7935508	0.3266011	-2.3159150

Fig. S25a

Sb	-0.3683121	-0.3131795	-1.6115584
C	0.4248060	-2.0936725	-0.6265830
C	1.7818347	-2.2926057	-0.3481375
C	-0.5147128	-3.0438652	-0.1997978
C	2.1831018	-3.4232089	0.3576981
H	2.5476472	-1.5829563	-0.6617412
C	-0.0937659	-4.1718710	0.4961655
H	-1.5833785	-2.9343228	-0.3945011
C	1.2561853	-4.3738110	0.7947801
C	1.5482733	0.6083862	-2.0995448
C	1.8521972	0.8780255	-3.4410602
C	2.4257281	1.0416003	-1.0926601
C	3.0104715	1.5771719	-3.7614614
H	1.2063689	0.5574298	-4.2590706
C	3.5839281	1.7304312	-1.4378487
H	2.2233338	0.8640597	-0.0364187
C	3.8922657	2.0159318	-2.7700503
C	-0.5170376	-1.2985364	-3.5626525
C	-1.4233274	-0.7712582	-4.4932239
C	0.2625692	-2.4108327	-3.9070828
C	-1.5412861	-1.3550101	-5.7512914
H	-2.0513623	0.0929950	-4.2689932
C	0.1266887	-2.9829094	-5.1663779
H	0.9805881	-2.8501549	-3.2132573
C	-0.7733204	-2.4665054	-6.1047038
C	-0.2868443	3.3322119	-0.1808282
C	-0.3684098	4.2285533	-1.3558037
C	-0.3008598	1.2463925	2.1821513
C	-0.3878676	-0.2255750	2.0952554
H	-1.3077439	-0.6104683	1.6423948
H	0.6811088	2.8357187	-0.0292226
O	0.4457694	0.5060678	1.1520364
O	-0.9545196	2.8913293	-1.3932062
C	-1.1340207	3.4304876	1.0643212
H	-0.6017523	4.0499626	1.8050096
H	-2.0731124	3.9527986	0.8354216
C	-1.4774336	2.0524299	1.6586881
H	-2.1832325	2.1785021	2.4953947
H	-1.9948928	1.4559240	0.8923219
C	0.3194161	-1.2016801	3.0003443
H	0.4272279	-2.1525034	2.4557661
H	1.3431119	-0.8434629	3.1796798

C	-0.3761608	-1.4315545	4.3509994
F	-0.9876109	-5.0924263	0.9199057
F	1.6493298	-5.4539649	1.4903814
F	3.4871583	-3.6270374	0.6418527
F	0.8641371	-4.0586429	-5.5155841
F	-0.9016178	-3.0312806	-7.3162046
F	-2.4106444	-0.8603638	-6.6585795
F	4.4355001	2.1568423	-0.4782696
F	5.0045141	2.6976568	-3.0899870
F	3.3083261	1.8530343	-5.0493135
C	-1.3616523	5.3646387	-1.4350870
H	-0.9066598	6.2978206	-1.0713397
H	-1.6624690	5.5220308	-2.4812698
H	-2.2661477	5.1627569	-0.8502819
C	0.8554495	4.3898761	-2.2295223
H	0.5604863	4.5047872	-3.2831885
H	1.4253020	5.2846719	-1.9379692
H	1.5095155	3.5141744	-2.1481189
C	0.5712377	1.9234373	3.2124380
H	1.0862137	2.7898218	2.7741300
H	-0.0466206	2.2829953	4.0482335
H	1.3359547	1.2460233	3.6080323
H	0.2234394	-2.1514922	4.9289169
C	-1.8252104	-1.9491545	4.3132653
C	-2.2858516	-2.2959412	5.7352130
H	-2.1670374	-1.4272051	6.3968456
H	-3.3478904	-2.5853456	5.7327357
H	-1.7104741	-3.1369666	6.1485034
C	-1.9902808	-3.1552455	3.3828416
H	-1.7903883	-2.8755784	2.3414204
H	-1.3067476	-3.9716253	3.6551882
H	-3.0174633	-3.5470064	3.4375453
O	-2.6178983	-0.8355805	3.8174396
H	-3.5391874	-1.1414728	3.7452629
H	-0.3839805	-0.4907030	4.9232127

Fig. S25b

Sb	-0.2079551	-1.2709735	0.0068369
C	-0.9195052	-3.2316754	-0.6545748
C	-0.0732388	-4.2889660	-1.0141293
C	-2.3116906	-3.3951714	-0.7251041
C	-0.6230184	-5.4927472	-1.4442049
H	1.0131501	-4.1992893	-0.9789100
C	-2.8373568	-4.6041653	-1.1653040
H	-3.0021827	-2.5850536	-0.4792787
C	-2.0070423	-5.6673233	-1.5309019
C	1.9083797	-1.8162004	-0.0772204
C	2.7584715	-1.3393566	-1.0823320
C	2.4350313	-2.6053057	0.9564848

C	4.1135550	-1.6511424	-1.0458588
H	2.3933872	-0.7132193	-1.8967963
C	3.7900376	-2.9207172	0.9664574
H	1.8161375	-2.9958034	1.7667081
C	4.6493312	-2.4441525	-0.0270833
C	-0.2428022	-0.4530000	-2.0144817
C	-0.0211321	0.9188033	-2.1934918
C	-0.5544292	-1.2537169	-3.1203444
C	-0.1372613	1.4740849	-3.4610821
H	0.2430738	1.5656253	-1.3599773
C	-0.6737376	-0.6755008	-4.3799928
H	-0.7230956	-2.3270360	-3.0285706
C	-0.4770794	0.6945086	-4.5700759
C	-1.4894587	2.7683924	0.6211999
C	-2.8502326	2.4955739	0.0917194
C	1.3352288	2.2747292	1.7526792
C	2.2115230	1.0857912	1.7165216
H	1.8378623	0.2162131	2.2672455
H	-0.7750771	3.1877619	-0.1033720
O	1.3941216	1.4241881	0.5599588
O	-1.8889851	1.4204690	0.2916499
C	-1.1232445	3.0421973	2.0601830
H	-0.8709100	4.1110274	2.1560256
H	-1.9843374	2.8620541	2.7182906
C	0.0431018	2.1620045	2.5457767
H	0.2679016	2.4063738	3.5973287
H	-0.2794559	1.1106900	2.5242123
C	3.7073361	1.1075158	1.5142965
H	4.0187638	0.1076640	1.1764964
H	3.9403463	1.7901876	0.6844995
C	4.5148674	1.5222642	2.7530041
H	5.5853628	1.5045636	2.4921399
H	4.2797227	2.5715902	3.0055220
F	-4.1762905	-4.7795857	-1.2555002
F	-2.5259604	-6.8317082	-1.9555771
F	0.1732098	-6.5256127	-1.7968123
F	-1.0007021	-1.4303510	-5.4513701
F	-0.6138948	1.2481693	-5.7866747
F	0.0527725	2.8023604	-3.6451523
F	4.3107274	-3.6846627	1.9513903
F	5.9609951	-2.7358212	0.0021042
F	4.9514957	-1.1831451	-1.9960355
C	-4.6868969	-0.6024346	-2.3654010
C	-3.7459686	0.5285638	-2.2200435
H	-2.7568045	0.3967524	-2.6774288
O	-3.9699774	-0.3812786	-1.1093777
C	-3.1130578	2.8508902	-1.3640351
H	-3.4468003	3.9015214	-1.4003327

H	-2.1599992	2.8026944	-1.9117816
C	-4.1560657	1.9739049	-2.0764687
H	-5.1269524	2.0468349	-1.5685275
H	-4.3051081	2.3723921	-3.0944446
C	4.3133070	0.6786631	4.0302307
O	2.9528292	0.8268391	4.5181931
H	2.8241090	1.7644270	4.7471126
C	-6.1849199	-0.4137439	-2.2844089
H	-6.4590637	0.4285503	-1.6391829
H	-6.6050882	-0.2418983	-3.2866158
H	-6.6514788	-1.3217531	-1.8751921
C	-4.2474859	-1.8207195	-3.1458382
H	-4.5704492	-1.7372304	-4.1939386
H	-3.1563910	-1.9233831	-3.1262127
H	-4.6913386	-2.7338526	-2.7245731
C	-4.0670390	2.4062615	0.9809689
H	-3.7949539	2.2659036	2.0331395
H	-4.6838936	3.3124788	0.8918534
H	-4.6683761	1.5386127	0.6752184
C	1.8863370	3.6684399	1.5562131
H	1.2033229	4.2728892	0.9425555
H	1.9972117	4.1738247	2.5273782
H	2.8600116	3.6528027	1.0546102
C	4.4714167	-0.8172387	3.7675973
H	5.4424157	-1.0345699	3.3022289
H	4.4000125	-1.3765315	4.7090808
H	3.6800840	-1.1788119	3.0998507
C	5.2918955	1.1467300	5.1151363
H	5.0897370	0.6151553	6.0546169
H	6.3343463	0.9568115	4.8216957
H	5.1852552	2.2281017	5.2976015

Fig. S26

Sb	-0.32010390	-0.84522550	0.57526420
C	-1.01730420	-2.80629480	-0.07628660
C	-2.40330000	-2.96560880	-0.21442040
C	-0.16080130	-3.88082910	-0.34713220
C	-2.91185740	-4.18968610	-0.62710470
H	-3.09906040	-2.14510170	-0.03009130
C	-0.69401820	-5.09388130	-0.76776320
H	0.92213560	-3.79662850	-0.24681890
C	-2.07191010	-5.26758320	-0.90837100
C	1.69341020	-1.54176960	1.06950730
C	2.80545380	-1.24428560	0.27214430
C	1.88084730	-2.17991720	2.30513550
C	4.08042450	-1.57589630	0.71670750
H	2.70706810	-0.73402260	-0.68637400
C	3.16301870	-2.51121170	2.72778820
H	1.04790590	-2.42272190	2.96760660

C	4.28076410	-2.21036860	1.94442260
C	0.23269970	-0.17344120	-1.42031550
C	0.27954770	1.20632580	-1.66305190
C	0.49052560	-1.08063500	-2.45784670
C	0.55944780	1.66268090	-2.94560320
H	0.09787650	1.93099470	-0.86947680
C	0.77693900	-0.59743610	-3.72971920
H	0.42346070	-2.16041630	-2.32812750
C	0.80514930	0.77438490	-3.99594900
C	-1.35031090	3.36301760	1.70348970
C	-2.67093870	2.89177850	1.20592430
C	1.59877200	2.73567720	2.40529280
C	2.55655550	1.66525670	2.08232250
H	2.30709850	0.67236160	2.48000310
H	-0.89012610	4.18294300	1.13479150
O	1.62137530	2.12653290	1.07173530
O	-1.45338860	2.15158190	0.92105500
C	-0.84755440	3.23747410	3.12737360
H	-0.64269760	4.25341670	3.50130710
H	-1.62871610	2.82241180	3.78021060
C	0.40518740	2.35201300	3.26251360
H	0.73892940	2.35375100	4.31259340
H	0.13112940	1.31667890	3.01168430
C	4.02861670	1.82383370	1.79200260
H	4.23241160	1.27232870	0.86017260
H	4.28312770	2.87159890	1.58197070
C	4.92370180	1.23976140	2.89421850
H	4.59483610	0.21017960	3.10905250
H	5.95352660	1.16261140	2.51476510
F	0.11331690	-6.13801270	-1.05797160
F	-2.57635730	-6.44469200	-1.32178860
F	-4.24807060	-4.35626490	-0.79228550
F	1.01363450	-1.44830990	-4.75462790
F	1.05696250	1.22875970	-5.23695260
F	0.58458130	2.98989240	-3.21157840
F	3.36096560	-3.11376330	3.92082130
F	5.52104420	-2.50495780	2.37306610
F	5.16760010	-1.26247440	-0.02372100
C	-3.85595940	0.35244150	-2.19245150
C	-3.28427420	1.51276900	-1.48093170
H	-2.25247720	1.77537060	-1.74715170
O	-3.37214290	0.22314350	-0.81355630
C	-3.26766030	3.58841860	-0.00609480
H	-3.90739880	4.41618640	0.34228670
H	-2.44437260	4.04018070	-0.58137400
C	-4.08672800	2.67004150	-0.93002870
H	-4.97491760	2.28947710	-0.40718520
H	-4.45465570	3.26822270	-1.78072560

C	4.97541070	1.99611470	4.23273170
O	3.61161060	2.02079160	4.73245670
H	3.63812600	2.40884520	5.62445960
C	-5.34711350	0.17967990	-2.36085550
H	-5.91180330	0.74139810	-1.60876040
H	-5.65792840	0.52042320	-3.35993020
H	-5.62008330	-0.88073920	-2.26771900
C	-2.97486100	-0.31891960	-3.23116440
H	-3.01710820	0.27613090	-4.15943120
H	-1.93972150	-0.26310940	-2.86963740
C	-3.64644880	2.15572270	2.09363530
H	-3.14827980	1.72388940	2.96876240
H	-4.44845590	2.82724840	2.43456740
H	-4.09754500	1.32780500	1.52955590
C	2.02498920	4.18167040	2.45988410
H	1.21440400	4.84601050	2.12810100
H	2.29051680	4.44994420	3.49280740
H	2.88882050	4.36912630	1.81249050
C	5.85915900	1.22188380	5.21948510
H	6.89462610	1.15029760	4.85698540
H	5.88140210	1.72758690	6.19773300
H	5.46755010	0.20559180	5.36319800
C	5.48618010	3.43293860	4.06748550
H	5.54166210	3.93318600	5.04645070
H	6.49235320	3.44827860	3.62406630
H	4.81205730	4.01538160	3.42785120
C	-2.13654660	-3.92128010	4.57234630
C	-2.19052680	-2.47481760	-4.27166980
H	-1.61296740	-1.82189740	-4.94225260
O	-1.34968600	-3.35626720	-3.48425990
C	-3.32362850	-1.78178830	-3.54440910
H	-3.51974450	-2.32698400	-2.61085390
H	-4.24169090	-1.83873150	-4.15173650
C	-3.25523190	-4.84985160	-4.15986570
H	-3.82878750	-4.46021240	-3.31170870
H	-3.94478050	-5.00631480	-5.00277890
H	-2.84781100	-5.82913470	-3.87169520
C	-1.30192090	-4.39733790	-5.74041680
H	-1.92066550	-4.50480780	-6.64385990
H	-0.48802650	-3.69130220	-5.94803690
H	-0.86044930	-5.37951180	-5.51367060

Fig. S27a

Sn	-1.8904545	-0.9335322	-0.1204370
C	-0.8756224	-0.3927109	1.6939598
C	-0.6921816	0.9351596	2.1017816
C	-0.3681984	-1.4449846	2.4708330
C	0.0140377	1.1925737	3.2732627
H	-1.0355988	1.7951850	1.5230634

C	0.3222506	-1.1636185	3.6455042	C	-1.7609677	1.9282979	-3.4920573
H	-0.4817205	-2.4921807	2.1840080	H	-0.3384270	0.7487635	-2.4237741
C	0.5328247	0.1539901	4.0558485	C	-3.0839702	2.3582622	-3.6259470
C	-0.8775161	-2.5742108	-1.0831246	C	0.2018871	3.5193479	-0.4748586
C	-1.3621875	-3.8789312	-0.9068662	C	-0.7551193	4.6442683	-0.4036907
C	0.2492884	-2.3560479	-1.8881572	C	2.3964041	1.1311328	-0.4309153
C	-0.7151255	-4.9448630	-1.5259896	C	2.1367368	-0.0639470	0.3883532
H	-2.2448522	-4.0953022	-0.3023160	H	1.8830755	0.1413100	1.4350647
C	0.8848969	-3.4341477	-2.4900252	H	-0.1790216	2.5919077	-0.9158175
H	0.6780484	-1.3633284	-2.0218570	O	1.1033418	0.4253668	-0.5209951
C	0.4160019	-4.7402032	-2.3219472	O	-0.4549426	3.8470379	0.7819108
C	-3.7877791	-1.7285359	0.5335849	C	1.7054039	3.6331655	-0.4933489
C	-4.6987900	-2.2656288	-0.3896096	H	2.0503382	3.6615133	-1.5401094
C	-4.1127457	-1.7134483	1.8975650	H	2.0114629	4.5830582	-0.0348012
C	-5.9150263	-2.7740371	0.0552603	C	2.3676523	2.4758551	0.2758075
H	-4.4923445	-2.3017935	-1.4606091	H	3.4166441	2.7237750	0.5008251
C	-5.3330992	-2.2285042	2.3235200	H	1.8543562	2.3667618	1.2433310
H	-3.4338590	-1.3073362	2.6494750	C	2.6693173	-1.4524165	0.1528950
C	-6.2490777	-2.7631300	1.4132148	H	1.8524410	-2.1593883	0.3751402
C	-2.3505614	0.5697548	-1.5871649	H	2.9096942	-1.5914399	-0.9118027
C	-3.6833123	0.9908305	-1.7112614	C	3.9013729	-1.8542730	1.0009699
C	-1.3860888	1.0409715	-2.4898584	F	0.2336785	2.4571738	3.6817121
C	-4.0363992	1.8780583	-2.7237771	F	1.2255808	0.4205946	5.1755988
H	-4.4687197	0.6411164	-1.0395454	F	0.8197926	-2.1633374	4.4066539

F	-5.6618679	-2.2229326	3.6317136
F	-7.4239453	-3.2553335	1.8339923
F	-6.8029525	-3.2911641	-0.8182117
F	-5.3104521	2.3024733	-2.8494716
F	-3.4275953	3.2268826	-4.5898871
F	-0.8420520	2.4126098	-4.3544248
F	1.9916313	-3.2398235	-3.2426824
F	1.0390023	-5.7735812	-2.9098254
F	-1.1687321	-6.2062849	-1.3686638
C	3.2350688	1.0602804	-1.6829275
H	2.8804937	1.7823725	-2.4317543
H	4.2792330	1.3021320	-1.4385611
H	3.1993165	0.0637530	-2.1385502
C	-0.3015565	6.0841606	-0.3438995
H	-0.2662549	6.5151844	-1.3555113
H	-1.0145124	6.6752886	0.2495494
H	0.6871669	6.1843435	0.1167623
C	-2.1539037	4.4376548	-0.9388771
H	-2.8790916	5.0175437	-0.3489552
H	-2.2244566	4.7697388	-1.9849375
H	-2.4334328	3.3783260	-0.8887014
C	4.3107624	-3.2785438	0.5840414
H	5.1652581	-3.6372461	1.1768516
H	3.4786877	-3.9810822	0.7368085
H	4.5917664	-3.3184284	-0.4792933
C	3.5740693	-1.8457325	2.5028567
H	2.7531727	-2.5396337	2.7285118
H	4.4480759	-2.1612610	3.0935407
H	3.2616900	-0.8546251	2.8622934
C	5.1022071	-0.9276718	0.7128527
H	5.2784231	-0.8835046	-0.3727205
H	6.0082058	-1.3608379	1.1780836
O	4.9312177	0.4347152	1.1249064
H	4.9225371	0.4562730	2.0966117

Fig. S27b

Sn	-1.7226726	0.2084631	-0.7633749
C	-1.1201746	0.9497529	1.1649540
C	-2.0111481	1.8820387	1.7196230
C	0.1129289	0.7204415	1.7814936
C	-1.6518487	2.5876370	2.8623629
H	-2.9828563	2.1024924	1.2738365
C	0.4522383	1.4427617	2.9168420
H	0.8170395	-0.0031787	1.3777651
C	-0.4122959	2.3887697	3.4719078
C	-0.8357576	-1.2664635	-2.0522245
C	-1.4725401	-2.5053607	-2.2027466
C	0.2645176	-0.9501342	-2.8573926
C	-0.9974377	-3.4136234	-3.1433231

H	-2.3415493	-2.7902738	-1.6077128
C	0.7220498	-1.8715705	-3.7887466
H	0.8048958	-0.0118765	-2.7506666
C	0.1025712	-3.1129999	-3.9502244
C	-3.7462215	-0.5004387	-0.4719600
C	-4.5388255	-0.8189927	-1.5860825
C	-4.2680553	-0.6790553	0.8175607
C	-5.8269739	-1.3107360	-1.4033846
H	-4.1783329	-0.7031258	-2.6096974
C	-5.5607934	-1.1674476	0.9808858
H	-3.6899094	-0.4457481	1.7134509
C	-6.3553834	-1.4929636	-0.1216818
C	-1.9014347	2.0094435	-1.9535887
C	-3.1555786	2.6137271	-2.1196959
C	-0.7633085	2.6264483	-2.4932580
C	-3.2585428	3.8188871	-2.8078626
H	-4.0705712	2.1713700	-1.7210597
C	-0.8851604	3.8339703	-3.1680404
H	0.2345938	2.2059039	-2.3747181
C	-2.1281053	4.4497793	-3.3341936
C	0.7260320	4.1432833	0.0284001
C	-0.1470533	5.3166167	0.2400957
C	2.8564421	1.7763452	-1.0224373
C	2.8664955	0.4678533	-0.3338674
H	2.9390175	0.5193734	0.7619555
H	0.2281331	3.1735701	-0.0863298
O	1.6163206	1.0215176	-0.8208311
O	0.5585968	4.6983828	1.3586980
C	2.1041462	4.2161441	-0.5861442
H	1.9997922	4.2725314	-1.6817999
H	2.5828314	5.1524744	-0.2709210
C	3.0018319	3.0320240	-0.1785478
H	4.0601510	3.3345030	-0.2390349
H	2.8064923	2.7846098	0.8760324
F	-2.4911331	3.5047665	3.3869256
F	-0.0600034	3.0936150	4.5556626
F	1.6582521	1.2514958	3.5049177
F	-6.0778556	-1.3431999	2.2152071
F	-7.5975443	-1.9732048	0.0453375
F	-6.5972852	-1.6288231	-2.4643747
F	-4.4580287	4.4166941	-2.9691820
F	-2.2329091	5.6277681	-3.9721667
F	0.2116631	4.4532856	-3.6662459
F	1.8075466	-1.5892492	-4.5475199
F	0.5674824	-4.0032851	-4.8419869
F	-1.5869229	-4.6206127	-3.2892450
C	3.2906380	1.8826383	-2.4672068
H	2.7236456	2.6655058	-2.9906853

H	4.3562184	2.1483811	-2.5259811
H	3.1395139	0.9389679	-3.0043649
C	0.3012752	6.7172142	-0.1165179
H	0.0376560	6.9461625	-1.1598036
H	-0.2080190	7.4478535	0.5290620
H	1.3813304	6.8484482	0.0104664
C	-1.6467668	5.1302945	0.2595368
H	-2.0871002	5.6869583	1.0996514
H	-2.0917234	5.5086861	-0.6715772
H	-1.9117068	4.0729865	0.3611789
C	3.4219202	-0.8198572	-0.9146511
H	4.4711810	-0.6314282	-1.1949248
H	2.8926296	-1.0726889	-1.8444020
C	3.3779634	-2.0011259	0.0766965
H	4.1503252	-2.7374769	-0.1908552
H	3.6353057	-1.6385187	1.0842015
C	2.0388205	-2.7208050	0.1130615
C	1.2826598	-2.7820703	1.3809362
H	1.7631498	-2.3094427	2.2441386
O	0.8973431	-1.8372998	0.3443859
C	1.8528096	-3.7630510	-0.9642266
H	2.5090170	-4.6233419	-0.7683682
H	0.8162596	-4.1106709	-1.0167568
H	2.1194838	-3.3543532	-1.9481850
C	0.2738851	-3.8530175	1.7218798
H	-0.4360450	-3.4432995	2.4582974
H	-0.3205583	-4.0729163	0.8235399
C	0.8898114	-5.1626227	2.2410562
C	1.7346423	-5.0845769	3.5248402
H	1.5302271	-5.5972254	1.4574025
H	0.0750273	-5.8813393	2.4192766
C	1.0070954	-4.3561558	4.6612523
H	0.8201871	-3.3067255	4.3995742
H	0.0443840	-4.8361210	4.8907891
H	1.6159083	-4.3731627	5.5781544
C	2.1344175	-6.5002073	3.9619289
H	1.2546440	-7.0964586	4.2444140
H	2.6624786	-7.0148603	3.1476364
H	2.8039155	-6.4576640	4.8347929
O	2.9308947	-4.3473852	3.1566516
H	3.4874073	-4.2746050	3.9520598

Fig. S27a

Sn	-1.2995010	-0.7545797	0.2422663
C	-1.3225389	0.0159956	2.2916163
C	-1.6022694	1.3515682	2.6157519
C	-1.1188725	-0.9109430	3.3256698
C	-1.6611911	1.7401220	3.9512618
H	-1.7594624	2.1251599	1.8611933

C	-1.1725882	-0.5021228	4.6525059	C	-1.3546346	1.5167808	-3.6007061
H	-0.9133858	-1.9649764	3.1242601	H	0.1116805	0.6325411	-2.2986442
C	-1.4434169	0.8264534	4.9853265	C	-2.7015937	1.8028016	-3.8348368
C	-0.4318233	-2.7458041	0.0023082	C	0.0522478	3.1835573	-0.0072178
C	-1.0221592	-3.8552176	0.6260683	C	-0.5771593	4.4129886	-0.5279737
C	0.7229920	-2.9234156	-0.7750954	C	1.8186156	0.4172539	0.5085093
C	-0.4558266	-5.1167725	0.4823971	H	-0.4101995	2.2383966	-0.2997911
H	-1.9363189	-3.7635803	1.2153003	O	0.6179901	0.1631301	-0.1724735
C	1.2600971	-4.1955726	-0.9187088	O	-0.7943824	4.0079087	0.8537864
H	1.2386117	-2.0754208	-1.2286822	C	1.5031518	3.0392518	0.3852285
C	0.6886220	-5.3069888	-0.2936848	H	2.1142076	3.0226123	-0.5322206
C	-3.4147522	-1.6090381	0.5320236	H	1.8052349	3.9365480	0.9461321
C	-3.9336307	-2.5153188	-0.4092122	C	1.7799228	1.7906124	1.2459557
C	-4.2245842	-1.2534517	1.6211527	H	2.7350869	1.9318610	1.7789117
C	-5.2069154	-3.0487466	-0.2557654	H	1.0032816	1.7182666	2.0230672
H	-3.3512537	-2.8321922	-1.2785012	F	-1.9351367	3.0237571	4.2880271
C	-5.4996441	-1.7917559	1.7640611	F	-1.4961084	1.2174338	6.2777904
H	-3.8791057	-0.5546625	2.3862168	F	-0.9666345	-1.3883968	5.6611096
C	-6.0090910	-2.6974039	0.8331371	F	-6.2853846	-1.4529753	2.8220578
C	-1.9041089	0.3628129	-1.5498254	F	-7.2448716	-3.2259946	0.9817943
C	-3.2601459	0.6375276	-1.7812816	F	-5.7104957	-3.9342248	-1.1575738
C	-0.9489997	0.8079992	-2.4797399	F	-4.9527347	1.6398169	-3.1394326
C	-3.6445799	1.3526958	-2.9108753	F	-3.0778344	2.5139488	-4.9205449
H	-4.0374617	0.2994126	-1.0943960	F	-0.4437715	1.9785674	-4.4990550

F	2.3757168	-4.4028008	-1.6732166
F	1.2297732	-6.5370199	-0.4386212
F	-1.0078384	-6.2001389	1.0902334
C	0.2076357	5.6939251	-0.7080079
H	0.5889302	5.7643795	-1.7382578
H	-0.4429229	6.5636093	-0.5280496
H	1.0556766	5.7501262	-0.0160448
C	-1.8002198	4.2844648	-1.4090086
H	-2.4982231	5.1148298	-1.2208925
H	-1.5177719	4.3075793	-2.4719298
H	-2.3194835	3.3376323	-1.2137320
C	2.1414303	-0.6719687	1.5504147
H	2.1798771	-1.6537961	1.0666261
H	3.1003775	-0.4924125	2.0616861
H	1.3563587	-0.6870882	2.3174314
C	2.9221133	0.4585571	-0.5872047
C	4.3379211	0.8789953	-0.1673028
H	2.5487267	1.1395059	-1.3746318
C	5.2175530	0.1402357	-1.1805437
H	4.5569899	0.5428457	0.8568747
H	4.4667016	1.9691432	-0.1923302
C	4.4654439	-1.1908566	-1.3837308
H	5.2488158	0.6934107	-2.1323605
H	6.2519182	-0.0125887	-0.8409979
O	3.0711962	-0.8449766	-1.1981201
C	4.8561215	-2.2414631	-0.3341097
H	4.7759608	-1.8297793	0.6806923
H	4.1771764	-3.1012300	-0.4070755
H	5.8874898	-2.5927094	-0.4927062
C	4.6067527	-1.7601485	-2.7942641
H	5.6544087	-2.0204470	-3.0093359
H	3.9931668	-2.6654002	-2.8963794
H	4.2650541	-1.0226424	-3.5337533

Fig. S28b

Sn	-0.12478950	-0.75024970	-0.01998100
C	0.04432170	-0.44531330	2.18288060
C	-0.64796850	0.58796290	2.83161300
C	0.80404100	-1.33501370	2.95622680
C	-0.54957930	0.73785960	4.20867240
H	-1.25715740	1.30097210	2.27599710
C	0.88332780	-1.17837610	4.33598720
H	1.35695450	-2.16080210	2.50476930
C	0.21511410	-0.13848460	4.98290890
C	0.19431910	-2.91972790	-0.14833270
C	-0.20758780	-3.81214960	0.85856980
C	0.79540630	-3.43615310	-1.30670980
C	-0.01574400	-5.18095070	0.70096250
H	-0.67055360	-3.46638320	1.78478410

C	0.98370370	-4.80542060	-1.44731250
H	1.12206300	-2.78068910	-2.11668070
C	0.58083890	-5.69776850	-0.45028290
C	-2.39123130	-0.62787830	0.22425460
C	-3.13758200	-1.77215700	0.54849480
C	-3.07212530	0.59717330	0.13001180
C	-4.50755040	-1.68508050	0.77619430
H	-2.67821060	-2.76046970	0.62021780
C	-4.44002710	0.67070950	0.36000960
H	-2.54773840	1.51478450	-0.13468890
C	-5.18107560	-0.46577690	0.68856560
C	-0.29145470	-0.29477400	-2.20425640
C	-1.54799310	-0.41346250	-2.81694720
C	0.80931340	0.06020440	-3.00180070
C	-1.69868650	-0.15940250	-4.17465720
H	-2.43543780	-0.69367360	-2.24716080
C	0.64058940	0.31678880	-4.35654280
H	1.80016340	0.13378550	-2.55429480
C	-0.61207950	0.21860970	-4.96533390
C	1.00201664	2.24096864	-0.53571832
C	-0.27626096	2.98218464	-0.64714872
H	1.27705454	1.65097974	-1.41520952
O	1.99448418	-0.70743102	-0.26965473
O	-0.20183826	1.72625754	0.10077858
C	2.15946934	2.55757394	0.37428478
H	2.22093676	3.65497122	0.46773572
H	1.97127926	2.16496453	1.38462059
C	3.49917726	1.98735196	-0.12954643
H	3.48989520	2.02340970	-1.23249198
H	4.30894077	2.65276284	0.20086578
F	-1.19787870	1.74962250	4.84713920
F	0.30003700	0.01328320	6.32457030
F	1.62206550	-2.03457150	5.09247370
F	-5.09865340	1.86034980	0.26708950
F	-6.51372410	-0.38907270	0.91051620
F	-5.23397350	-2.79286260	1.08719850
F	-2.91645060	-0.26832700	-4.77164270
F	-0.76755460	0.47363360	-6.28519210
F	1.70127230	0.67179290	-5.13290890
F	1.56262920	-5.31888590	-2.56467530
F	0.76391340	-7.02910630	-0.59708890
F	-0.40832500	-6.05324110	1.66639510
C	-0.59249696	4.18607684	0.20759478
H	-0.33033156	5.11412974	-0.32322652
H	-1.67055326	4.21425394	0.42643298
H	-0.05205536	4.15676744	1.16023438
C	-1.04843786	2.88942724	-1.94590462
H	-2.13176416	2.93202444	-1.75756532

H	-0.78625976	3.73106584	-2.60465242
H	-0.82448946	1.94893994	-2.46527582
C	3.14234728	-0.39371719	0.52337360
C	3.90749368	-1.66397627	0.83443919
C	4.01199940	0.60439921	-0.21419810
H	2.77675530	0.06514918	1.47938275
C	5.17850668	-1.36818407	1.60423079
H	4.16544761	-2.18744827	-0.12357997
H	3.25906980	-2.35590541	1.43227922
C	6.04777112	-0.36896418	0.86862294
H	5.75109555	-2.31862151	1.76327819
H	4.91534424	-0.95997890	2.61541461
C	6.66281375	-1.04698642	-0.36976650
H	6.01010184	-0.91846956	-1.20783010
H	7.61188202	-0.60237161	-0.58537514
H	6.79379201	-2.09121131	-0.17653330
C	7.28190908	-0.03000616	1.72513387
H	7.82940761	-0.92574387	1.93201382
H	7.90777959	0.65574783	1.19322449
H	6.96602512	0.41548728	2.64527131
O	5.28264211	0.90079634	0.55600985
C	4.30297134	0.07701073	-1.63151835
H	5.25466105	0.44035778	-1.95887995
H	4.31489982	-0.99284373	-1.61851241
H	3.54152185	0.41708605	-2.30192379

7. Supporting references

S1 J. López-Andarias, J. Saarbach, D. Moreau, Y. Cheng, E. Derivery, Q. Laurent, M. González-Gaitán, N. Winssinger, N. Sakai and S. Matile, *J. Am. Chem. Soc.*, 2020, **142**, 4784–4792.

S2 D. Bulfield and S. M. Huber, *J. Org. Chem.*, 2017, **82**, 13188–13203.

S3 T. A. Gazis, A. Ayan Dasgupta, M. S. Hill, J. M. Rawson, T. Thomas Wirth, L. Rebecca and R. L. Melen, *Dalton Trans.*, 2019, **48**, 12391–12395.

S4 D. Tofana and F. P. Gabbaï, *Chem. Sci.*, 2016, **7**, 6768–6778.

S5 (*a*) S. Benz, A. I. Poblador-Bahamonde, N. Low-Ders and S. Matile, *Angew. Chem. Int. Ed.*, 2018, **57**, 5408-5412. (*b*) M. Yang, D. Tofan, C.-H. Chen, K. M. Jack and F. P. Gabbaï, *Angew. Chem. Int. Ed.*, 2018, **57**, 13868–13872.

S6 J. Chen, K. Sakamoto, A. Orita and J. Otera, *J. Organomet. Chem*., 1999, **574**, 58-65

S7 X. Zhang, X. Hao, L. Liu, A.-T. Pham, J. López-Andarias, A. Frontera, N. Sakai and S. Matile, *J. Am. Chem. Soc.*, 2018, **140**, 17867–17871.

S8 M. Paraja, X. Hao and S. Matile, *Angew. Chem. Int. Ed.*, 2020, in press, 10.1002/anie.202000681.

S9 D. A. L. Otte, D. E. Borchmann, C. Lin, M. Weck and K. A. Woerpel, *Org. Lett.*, 2014, **16**, 1566–1569.

S10 T. B. Towne and F. E. McDonald, *J. Am. Chem. Soc.,* 1997, **119**, 6022–6028.

S11 R. Ahlrichs, M. Bär, M. Hacer, H. Horn and C. Kömel, *Chem. Phys. Lett.*, 1989, **162**, 165–169.

S12 S. Grimme, J. Antony, S. Ehrlich, and H. A. Krieg, *J. Chem. Phys.* 2010, **132**, 154104–154119.

S13 Y. Shao, L. F. Molnar, Y. Jung, J. Kussmann, C. Ochsenfeld, S. T. Brown, A. T. B. Gilbert, L. V. Slipchenko, S. V. Levchenko, D. P. O'Neill, R. A. DiStasio Jr., R. C. Lochan, T. Wang, G. J. O. Beran, N. A. Besley, J. M. Herbert, C. Y. Lin, T. Van Voorhis, S. H. Chien, A. Sodt, R. P. Steele, V. A. Rassolov, P. E. Maslen, P. P. Korambath, R. D. Adamson, B. Austin, J. Baker, E. F. C. Byrd, H. Dachsel, R. J. Doerksen, A. Dreuw, B. D. Dunietz, A. D. Dutoi, T. R: Furlani, S. R. Gwaltney, A. Heyden, S. Hirata, C.-P. Hsu, G. Kedziora, R. Z. Khalliulin, P. Klunzinger, A. M. Lee, M. S. Lee, W. Z. Liang, I. Lotan, N. Nair, B. Peters, E. I. Proynov, P. A. Pieniazek, Y. M. Rhee, J. Ritchie, E. Rosta, C. D. Sherrill, A. C. Simmonett, J. E. Subotnik, H. L. Woodcock III, W. Zhang, A. T. Bell, A. K. Chakraborty, D. M. Chipman, F. J. Keil, A. Warshel, W. J. Hehre, H. F. Schaefer, J. Kong, A. I. Krylov, P. M. W. Gill and M. Head-Gordon, *Phys. Chem. Chem. Phys.*, 2006, **8**, 3172–3191.

S14 A. Klampt and G. Schüürmann, *J. Chem. Soc.*, *Perkin Trans. 2*, 1993, 799–805.

S15 A. Klampt, *Comput. Mol. Sci.*, 2011, **1**, 699–709.

S16 M. Michalczyk, W. Zierkiewicz, R. Wysokiński and S. Scheiner, *ChemPhysChem*, 2019, **20**, 959–966.

S17 (*a*) L. R. Cook, H. Oinuma, M. A. Semones and Y. J. Kishi, *J. Am. Chem. Soc.*, 1997, **119**, 7928-7937. (*b*) I. Vilotijevic and T. F. Jamison, *Science*, 2007, **317**, 1189–1192. (*c*) K. C. Nicolaou, J. H. Seo, T. Nakamura and R. J. Aversa, *J. Am. Chem. Soc.*, 2011, **133**, 214–219. (*d*) A. Hoshino, H. Nakai, M. Morino, K. Nishikawa, T. Kodama, K. Nishikibe and Y. Morimoto, *Angew. Chem. Int. Ed.*, 2017, **56**, 3064–3068.

S18 H. Mahalakshmi, V. K. Jain and E. R. T. Tiekink, *Z. Kristallogr. – New Cryst.Struct.*, 2004, **218**, 71–72.

The original data can be found at:

https://dx.doi.org/10.26037/yareta:qvwfjnq5mnd4fnv4x2cq3lr2v4

8. NMR spectra

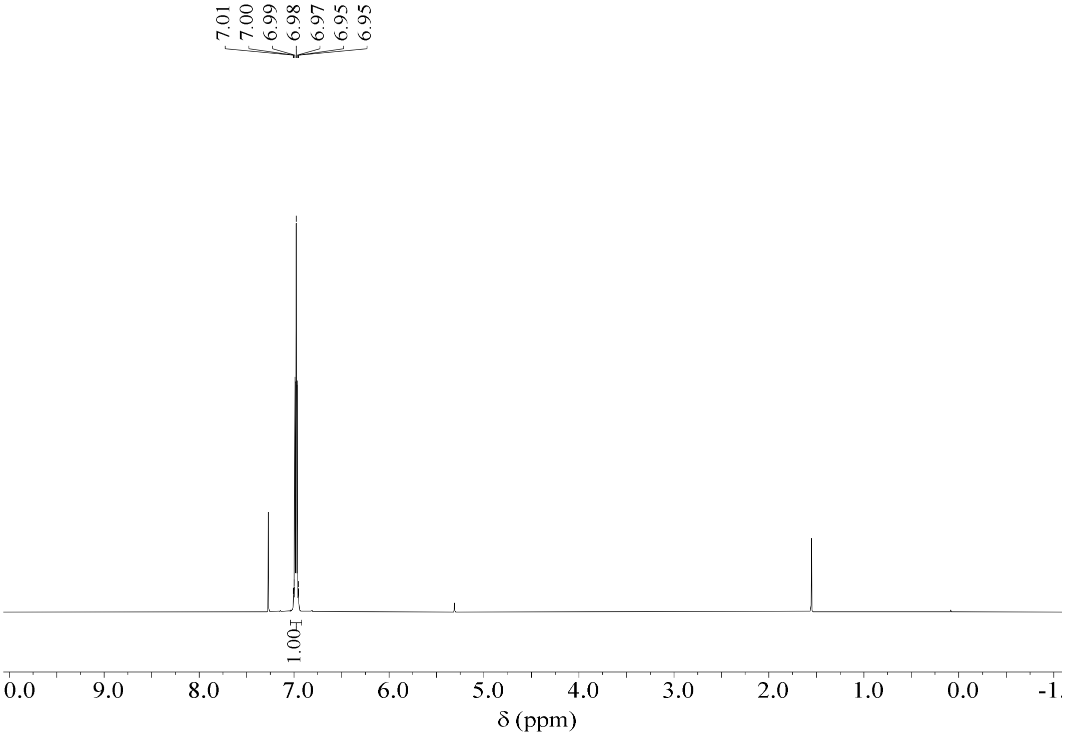

Fig. S29 ^{1}H NMR spectrum of **1** in $CDCl_3$.

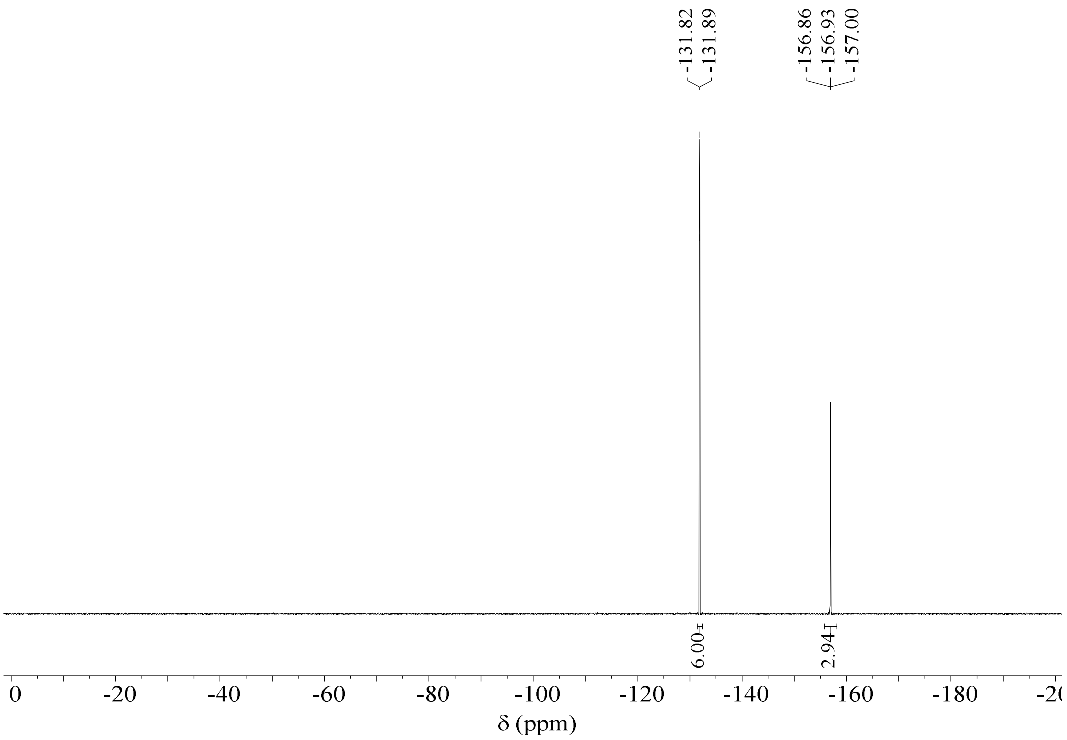

Fig. S30 ^{19}F NMR spectrum of **1** in $CDCl_3$.

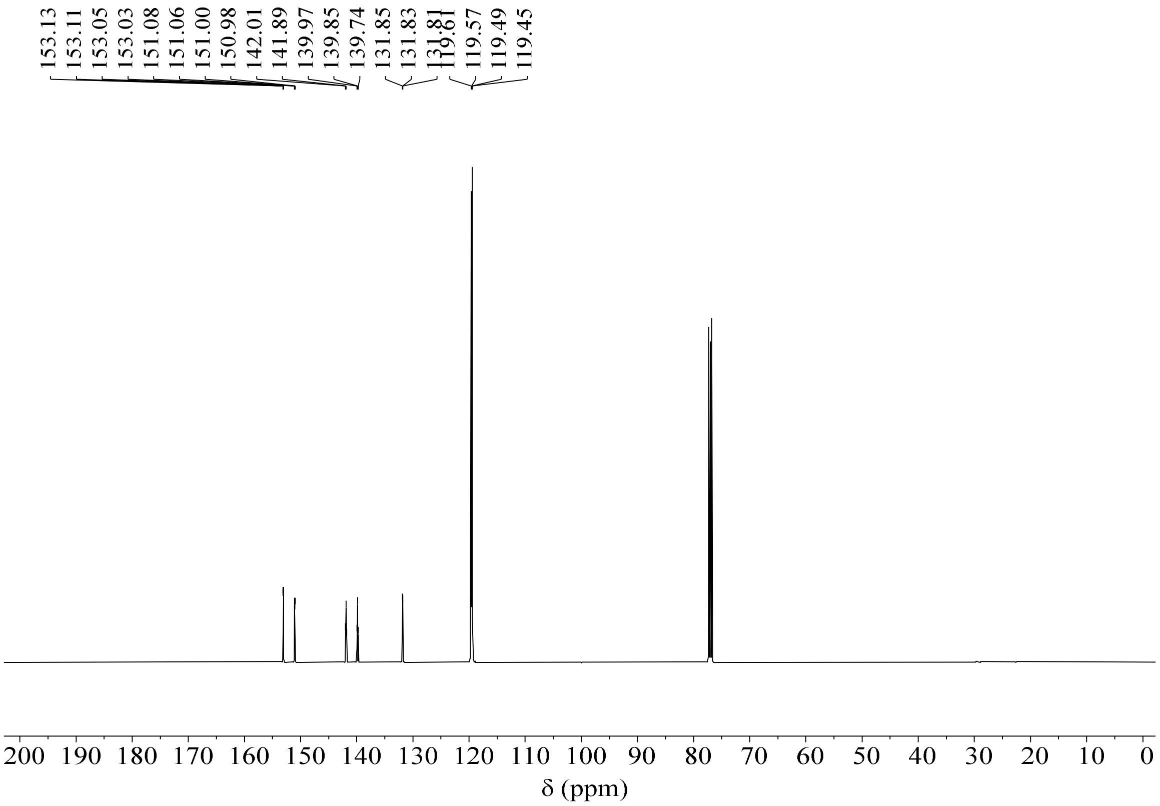

Fig. S31 ^{13}C NMR spectrum of **1** in $CDCl_3$.

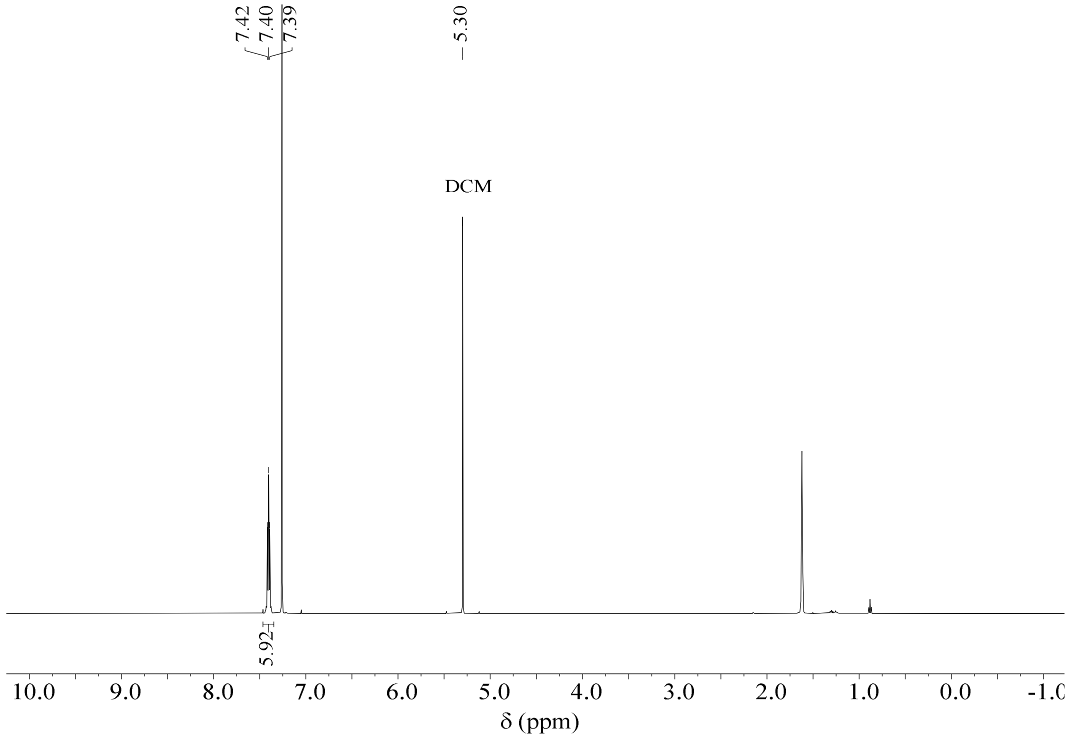

Fig. S32 ^{1}H NMR spectrum of **2** in $CDCl_3$.

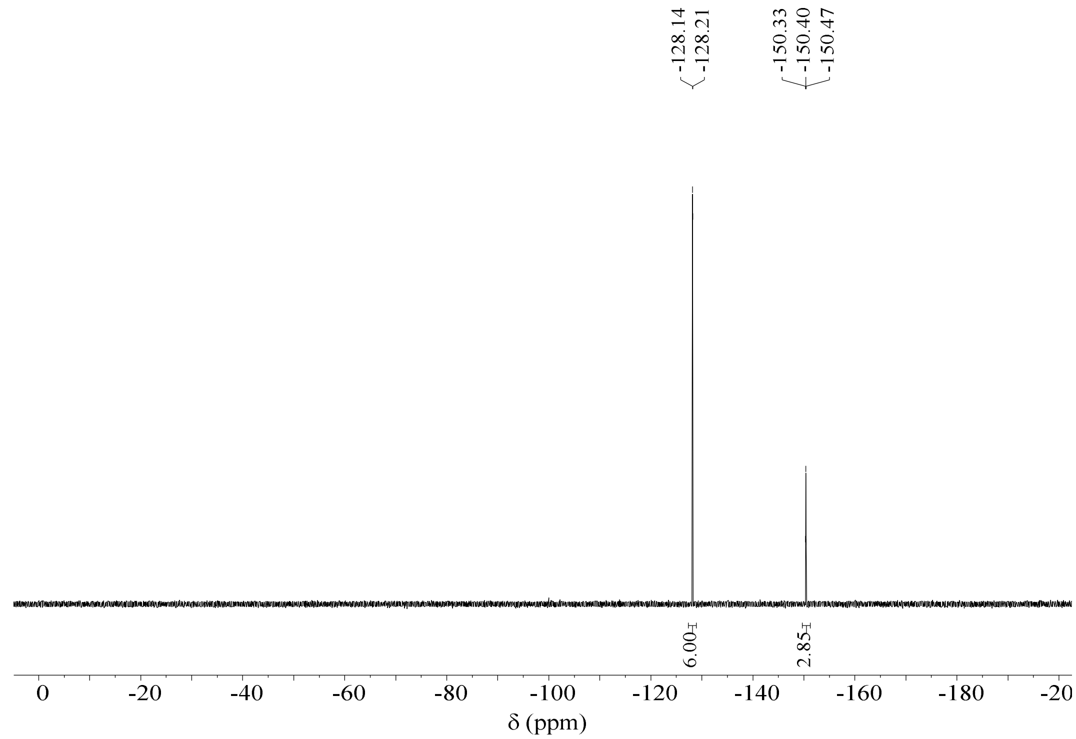

Fig. S33 ^{19}F NMR spectrum of **2** in $CDCl_3$.

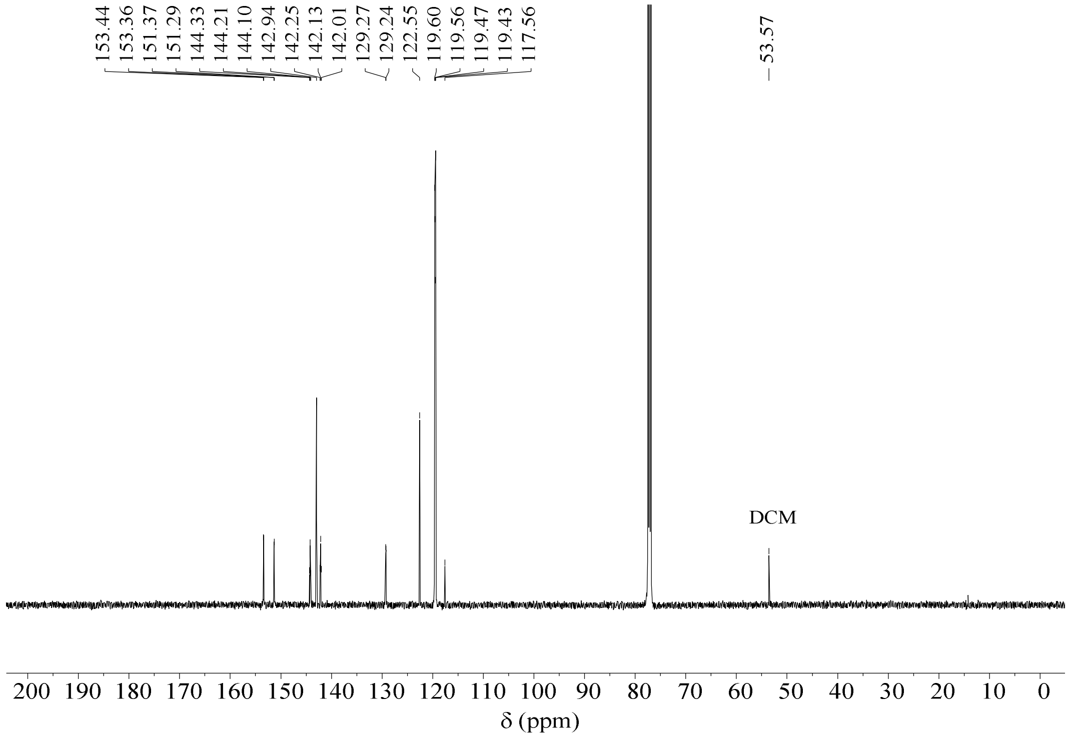

Fig. S34 ^{13}C NMR spectrum of **2** in $CDCl_3$.

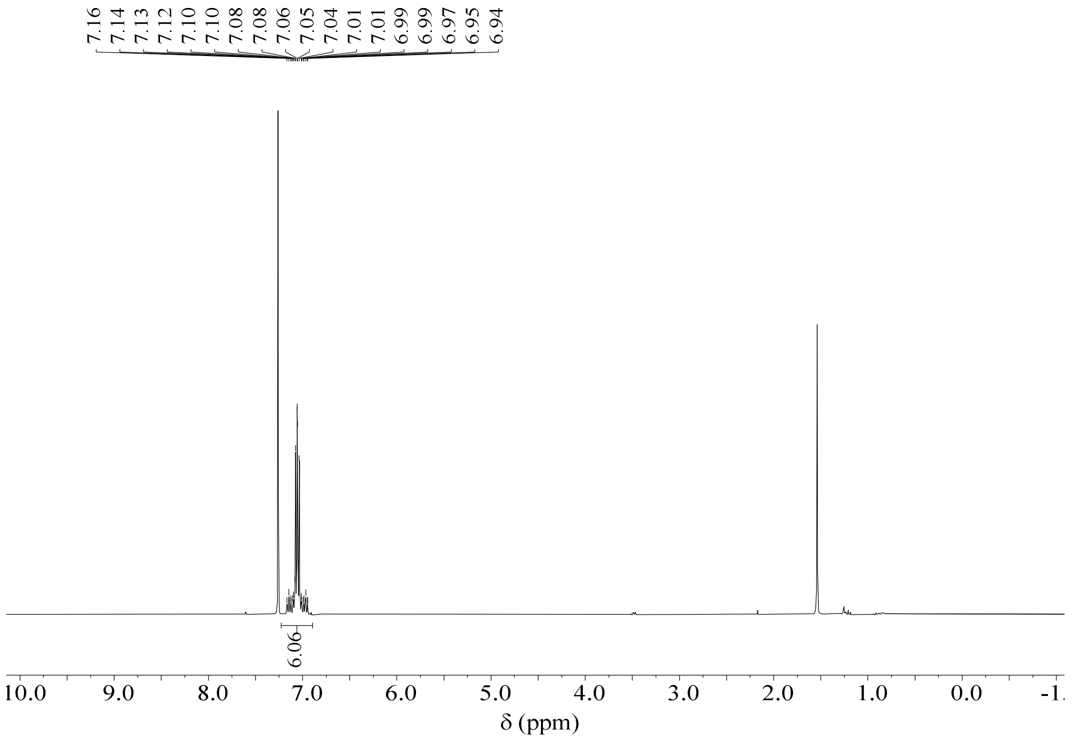

Fig. S35 ^{1}H NMR spectrum of **3** in $CDCl_3$.

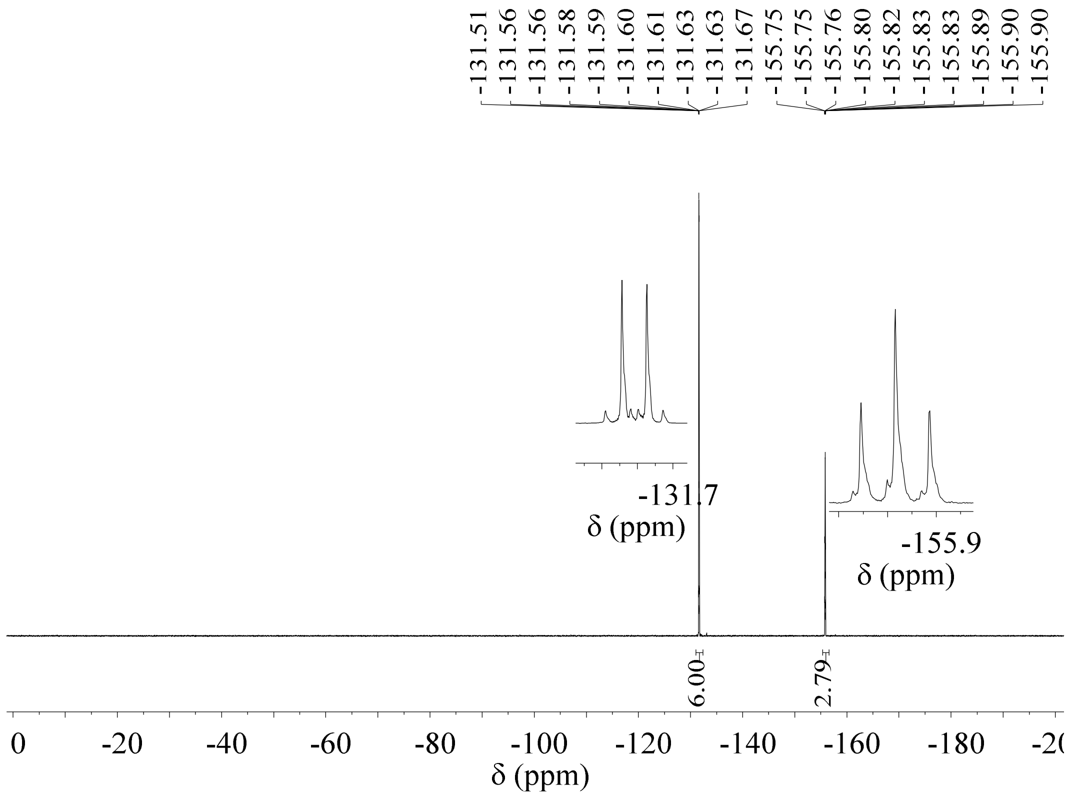

Fig. S36 ^{19}F NMR spectrum of **3** in $CDCl_3$.

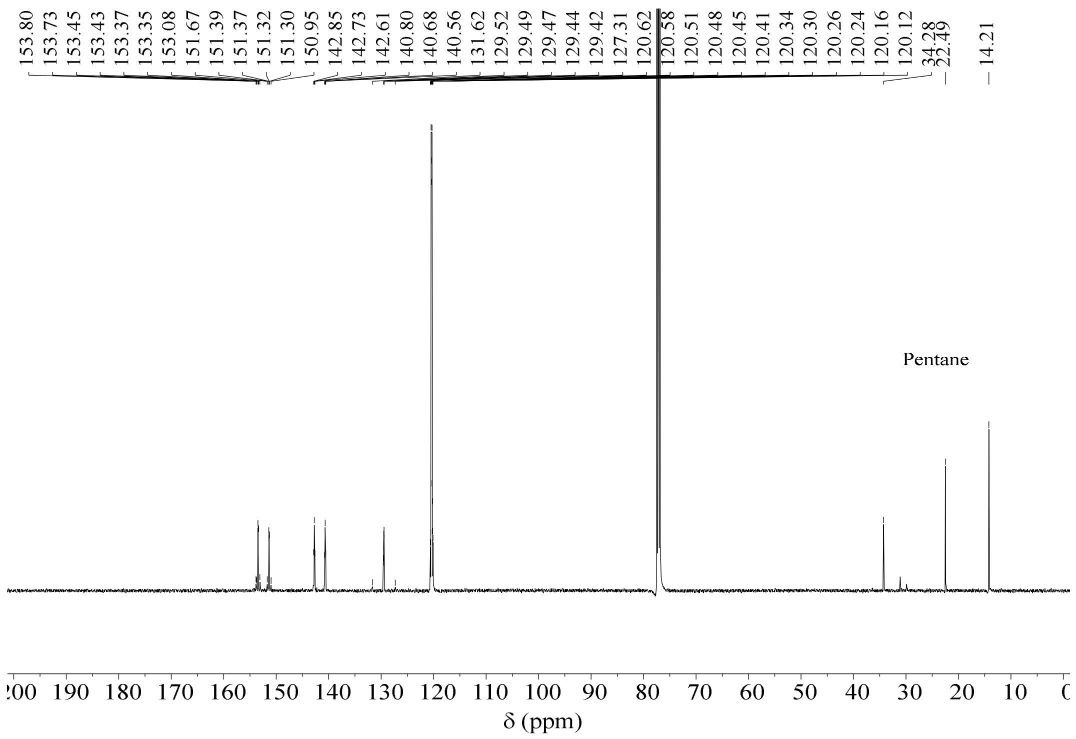

Fig. S37 ^{13}C NMR spectrum of **3** in $CDCl_3$.

Analysis. In all the signals (in ^{1}H, ^{13}C, and ^{19}F spectra) were observed two further couplings with NMR active ^{117}Sn and ^{119}Sn nucleus.

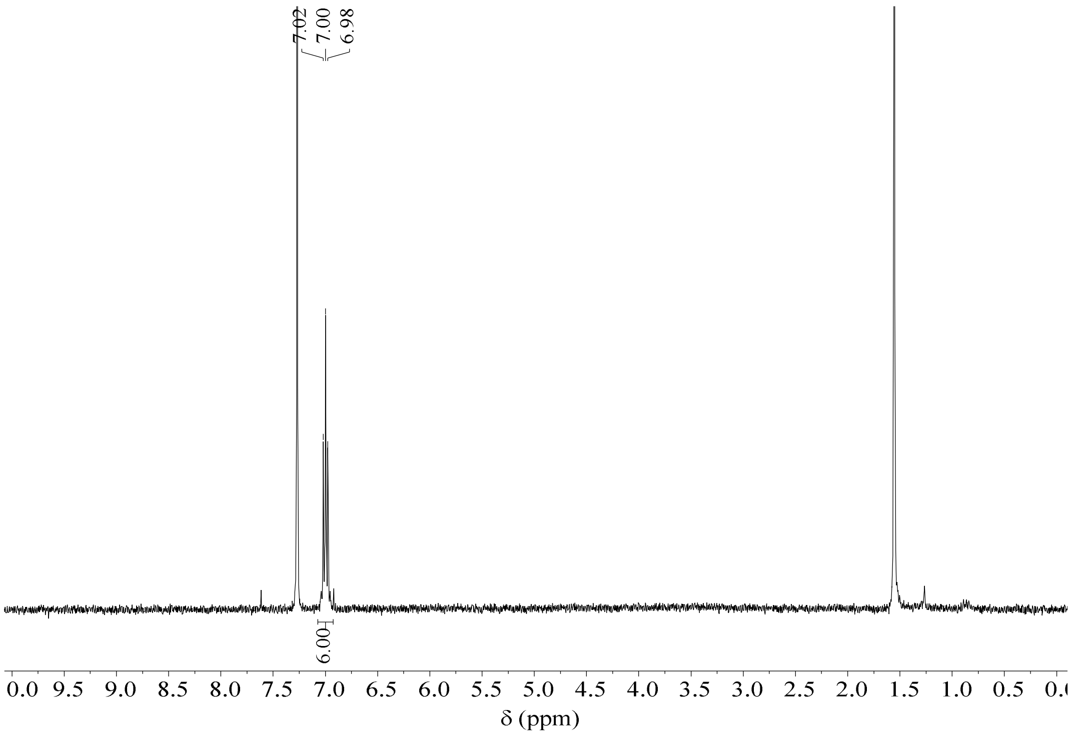

Fig. S38 ^{1}H NMR spectrum of **4** in $CDCl_3$.

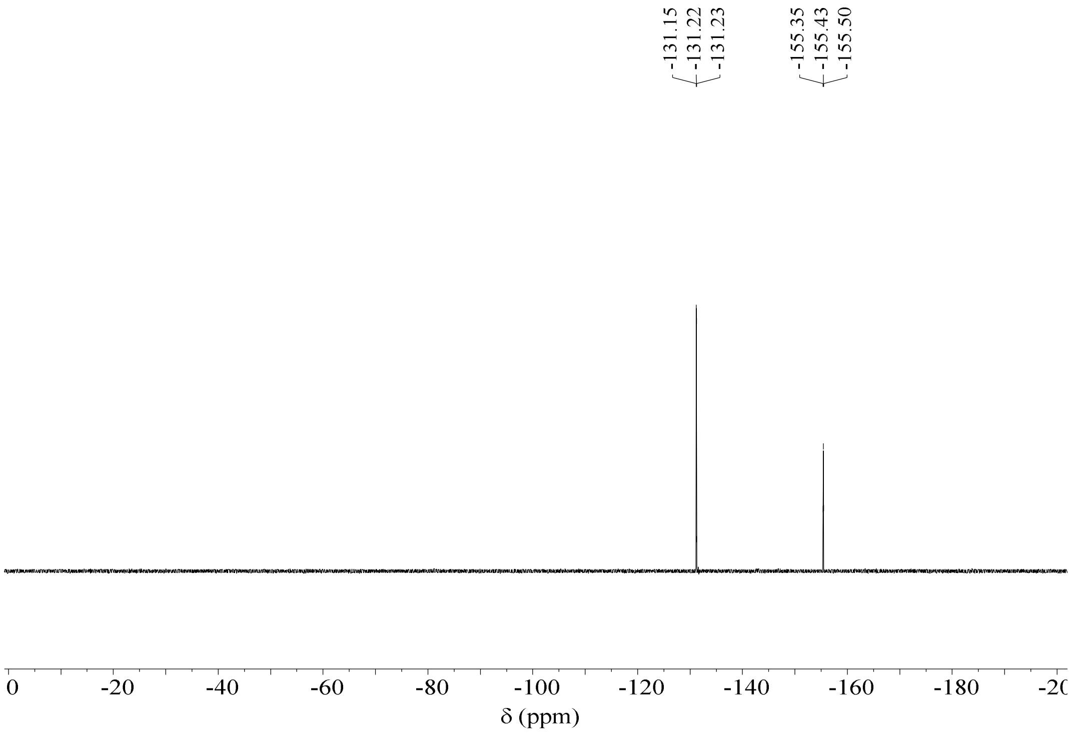

Fig. S39 ^{19}F NMR spectrum of **4** in $CDCl_3$.

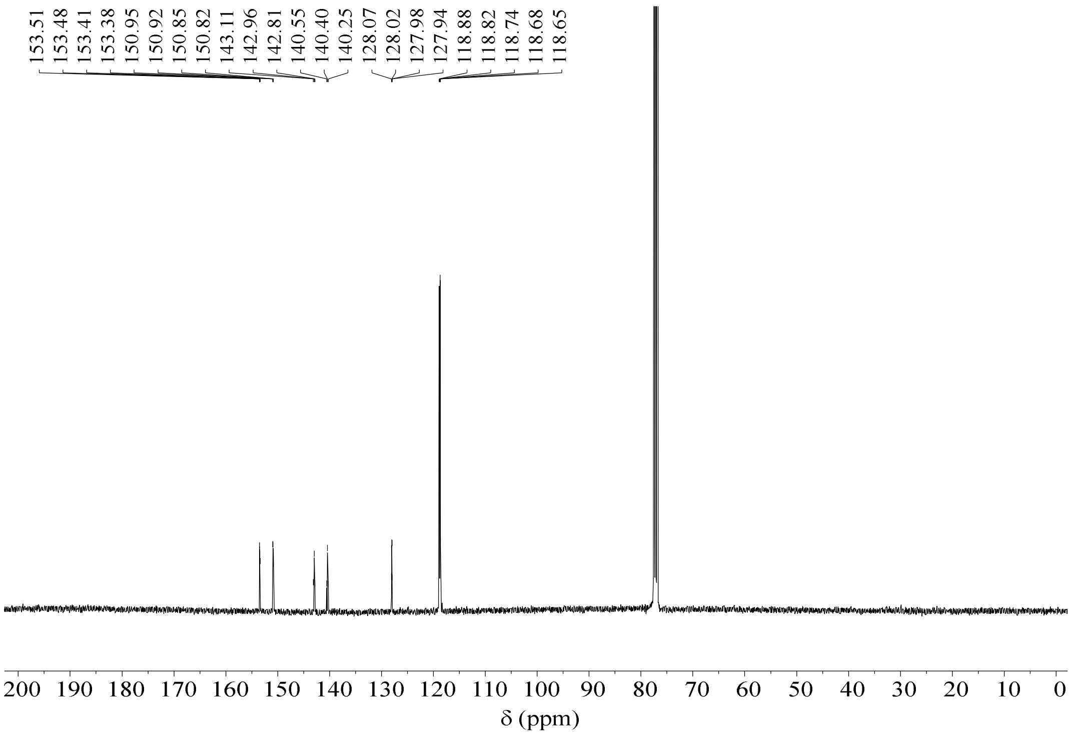

Fig. S40 ^{13}C NMR spectrum of **4** in $CDCl_3$.

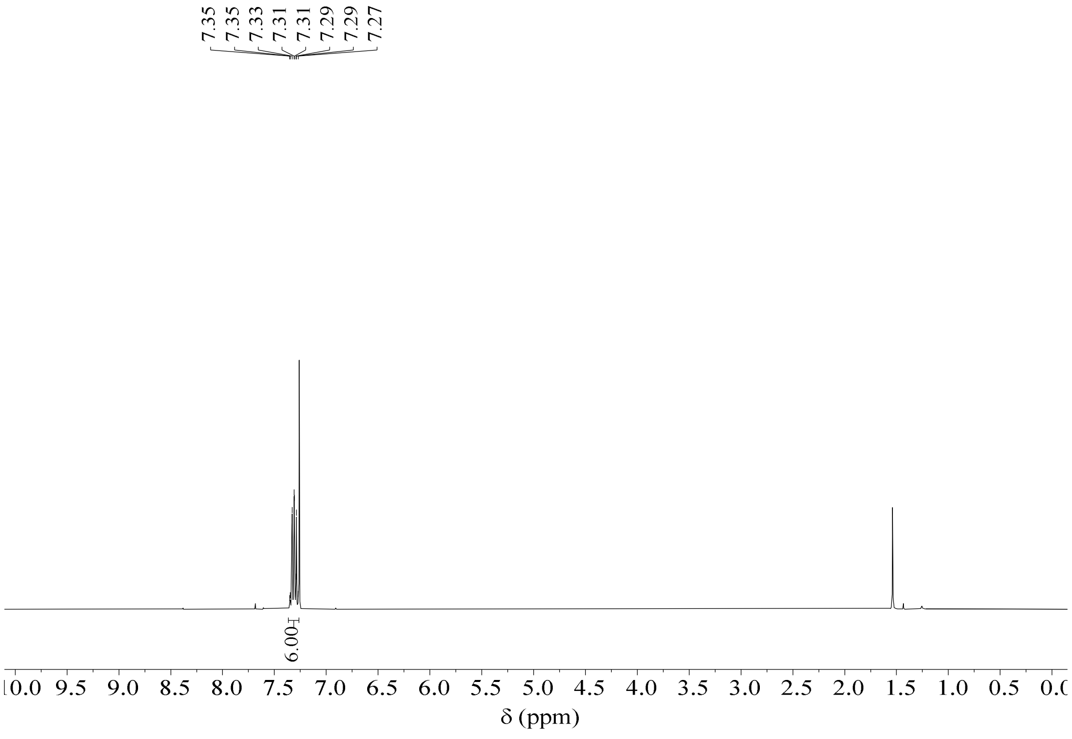

Fig. S41 ^{1}H NMR spectrum of **6** in $CDCl_3$.

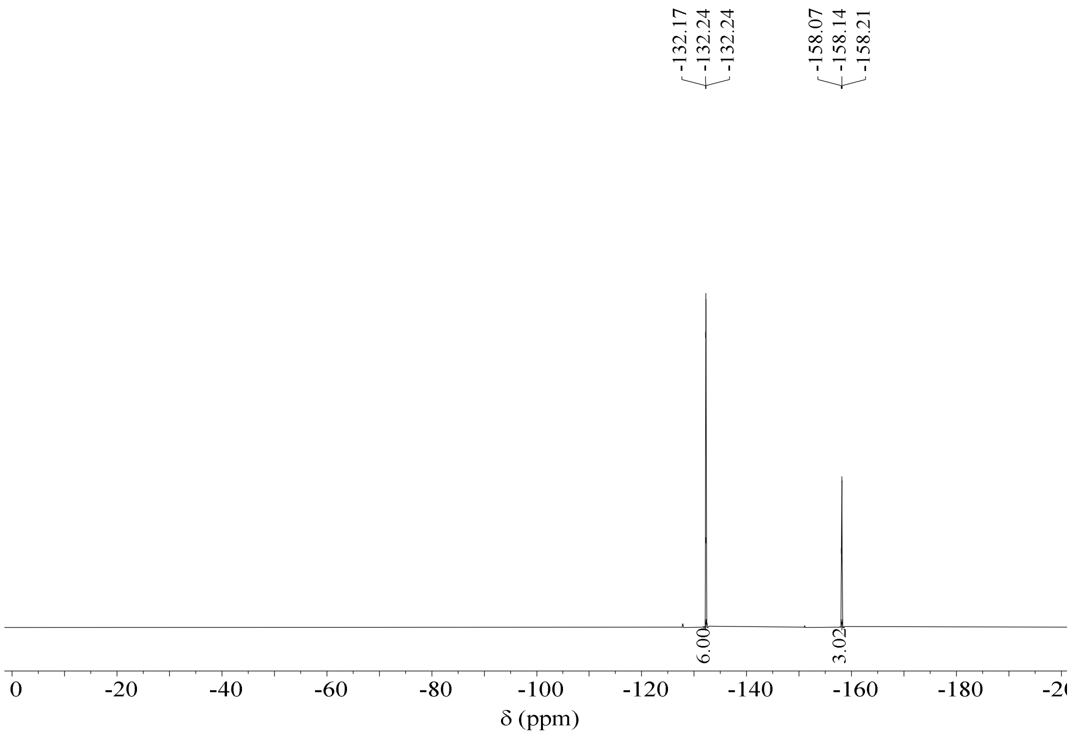

Fig. S42 ^{19}F NMR spectrum of **6** in $CDCl_3$.

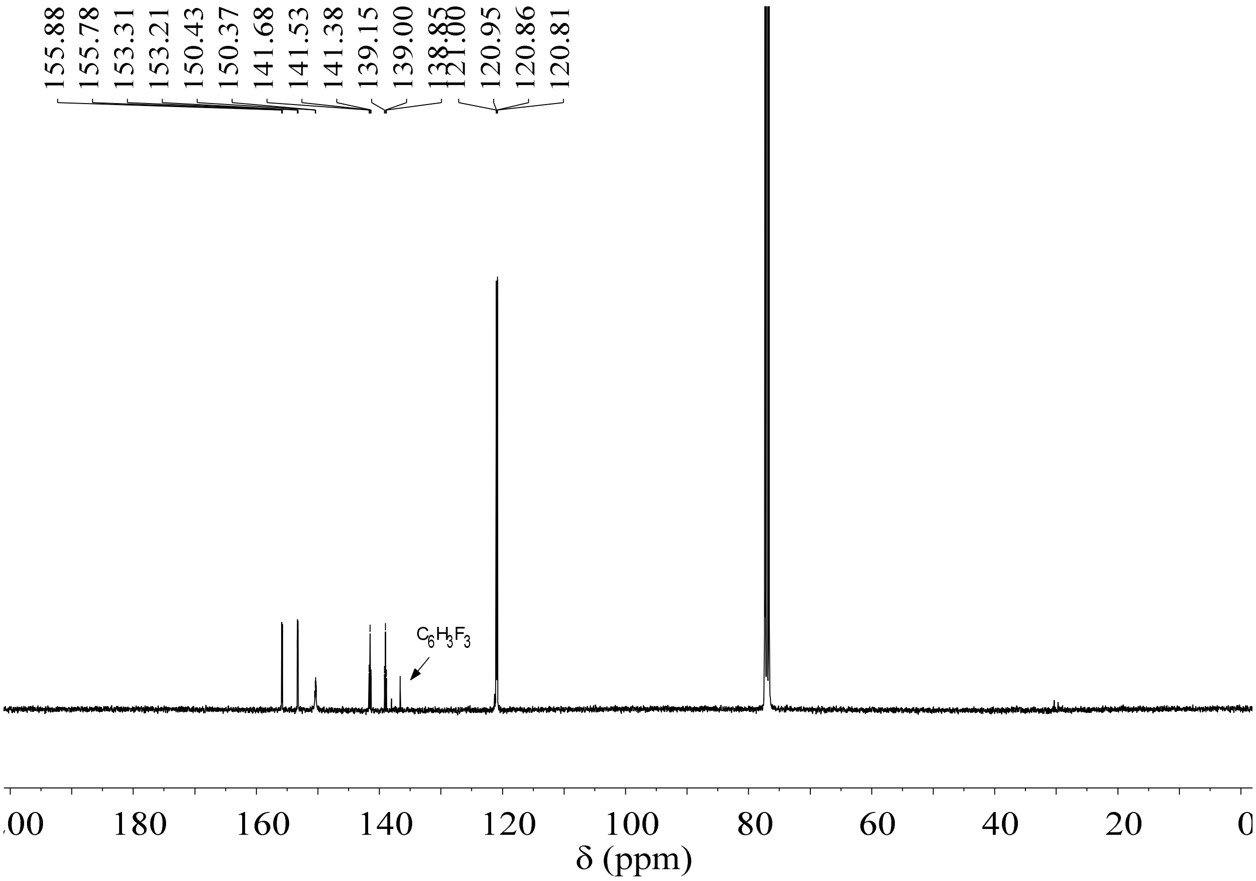

Fig. S43 ^{13}C NMR spectrum of **6** in $CDCl_3$.

Analysis. The catalyst slightly decomposed in $CDCl_3$ during the time needed to measure the ^{13}C NMR spectrum. However, it was stable in CD_2Cl_2 under the conditions of the catalytic reactions.

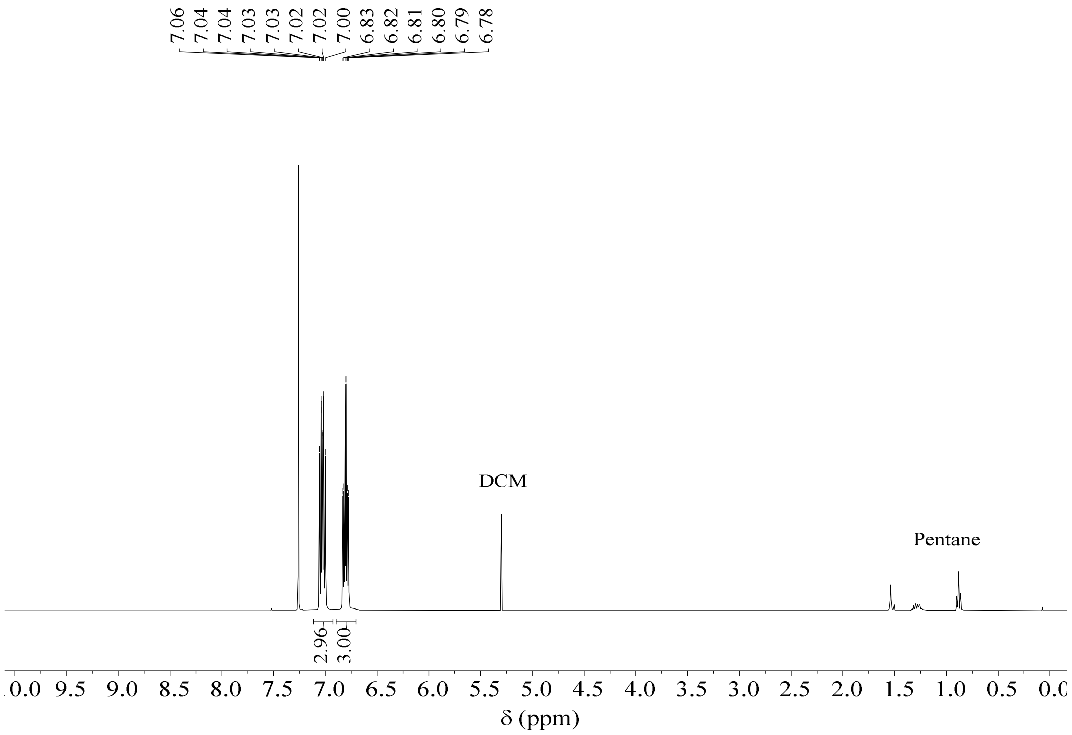

Fig. S44 ^{1}H NMR spectrum of **9** in $CDCl_3$.

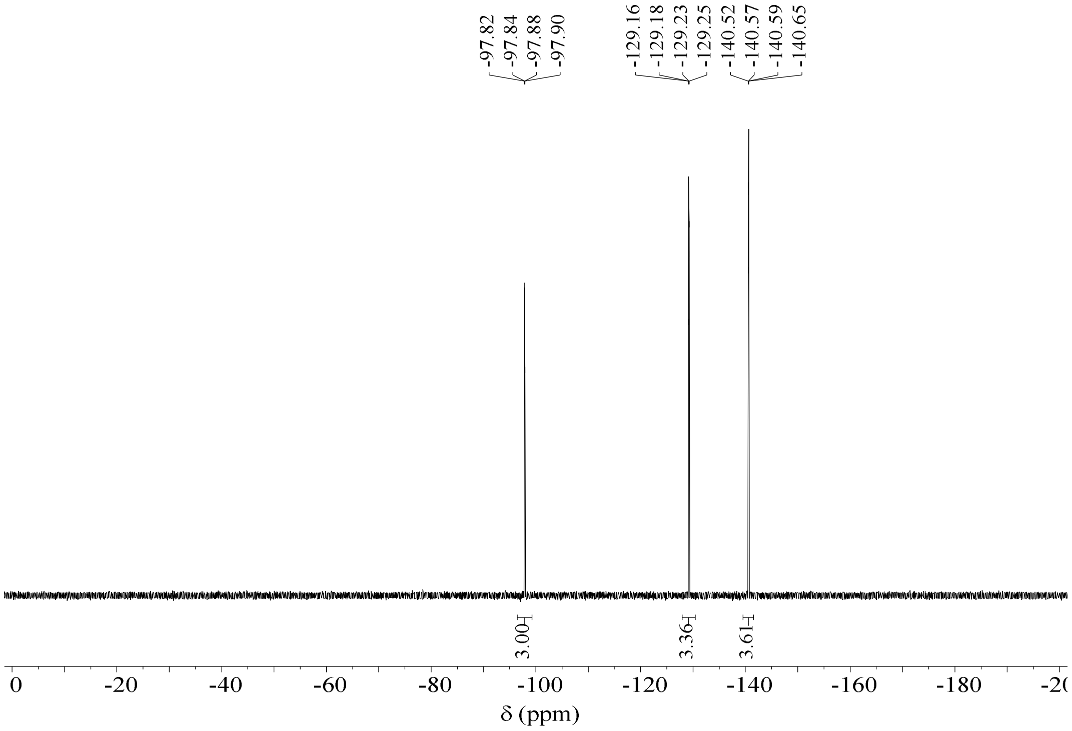

Fig. S45 ^{19}F NMR spectrum of **9** in $CDCl_3$.

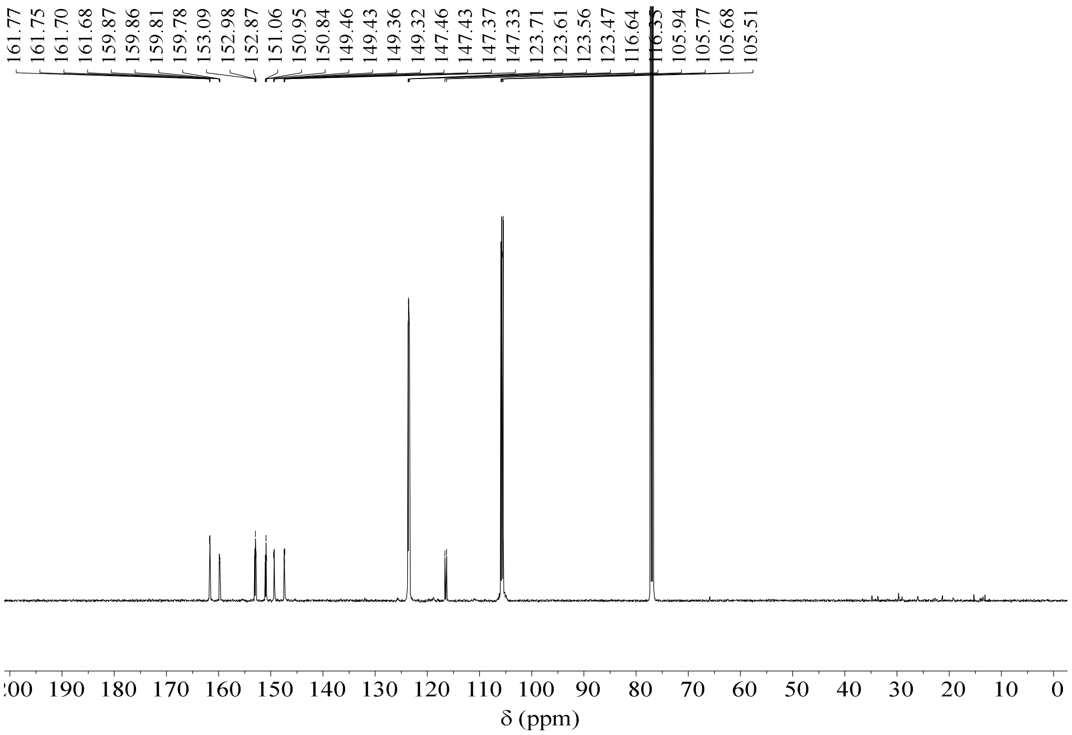

Fig. S46 ^{13}C NMR spectrum of **9** in $CDCl_3$.

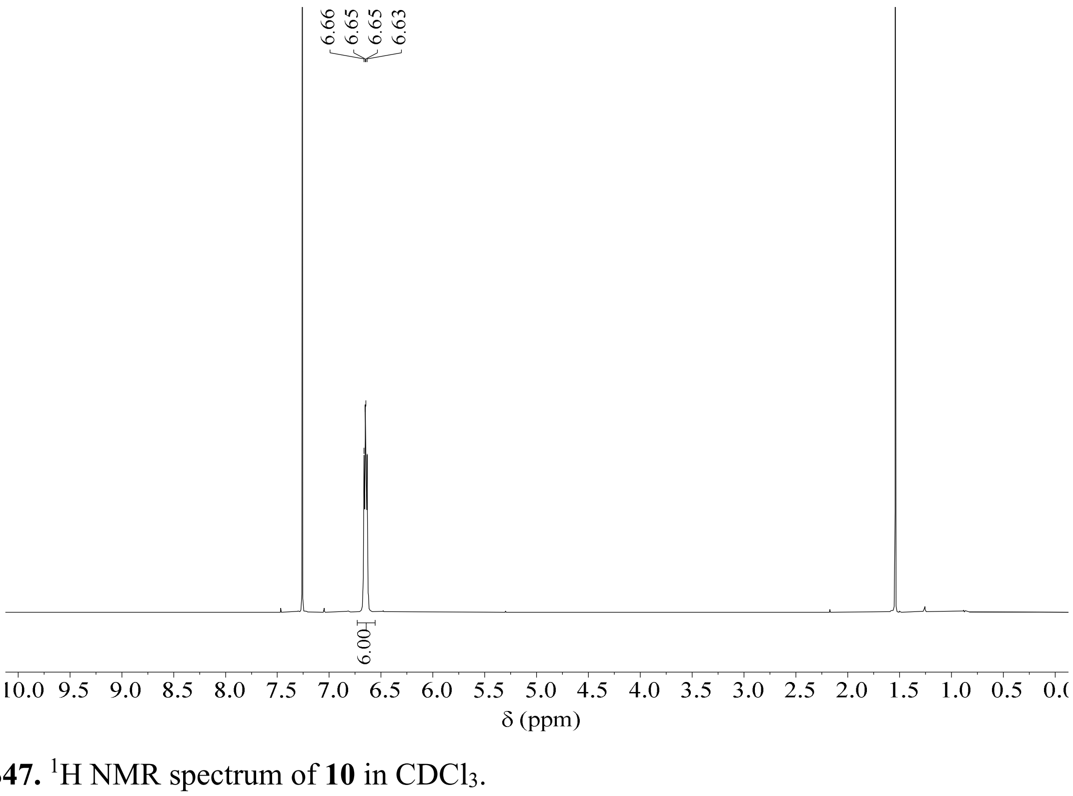

Fig. S47. ^{1}H NMR spectrum of **10** in $CDCl_3$.

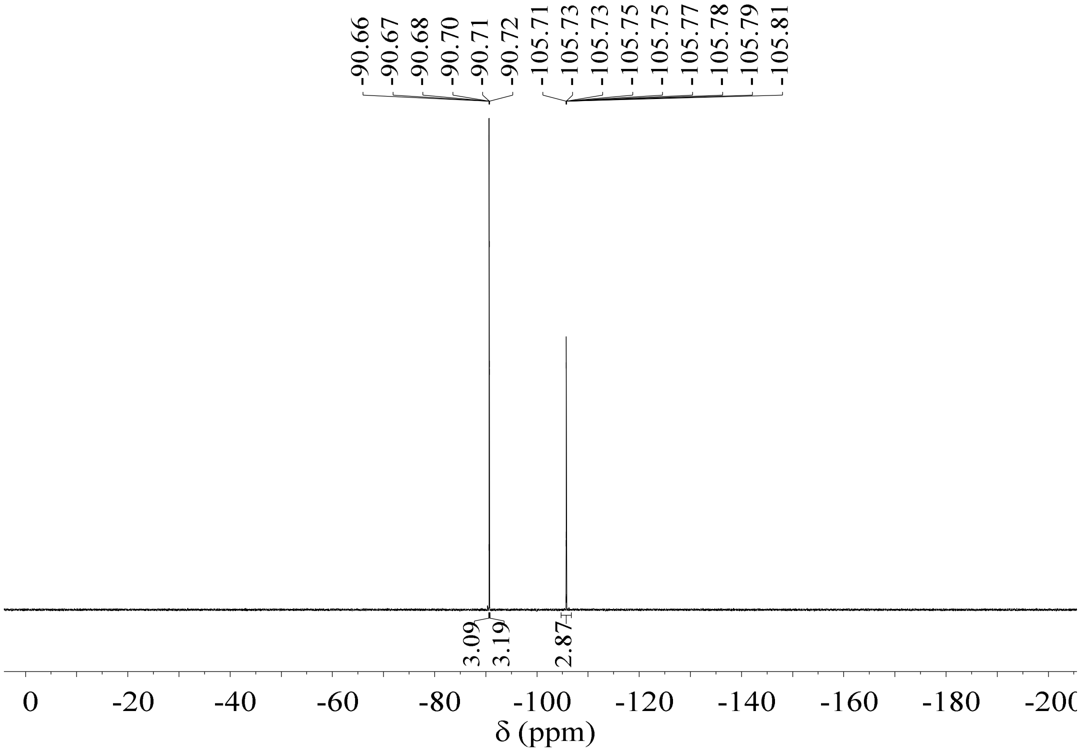

Fig. S48 ^{19}F NMR spectrum of **10** in $CDCl_3$.

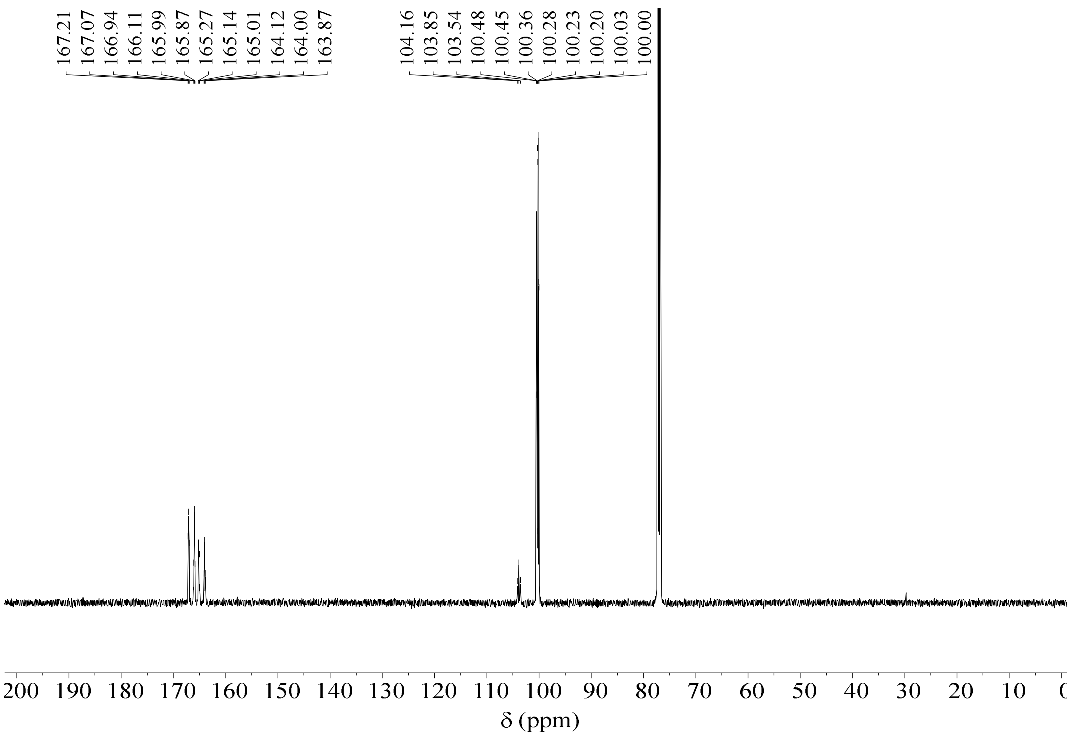

Fig. S49 ^{13}C NMR spectrum of **10** in $CDCl_3$.

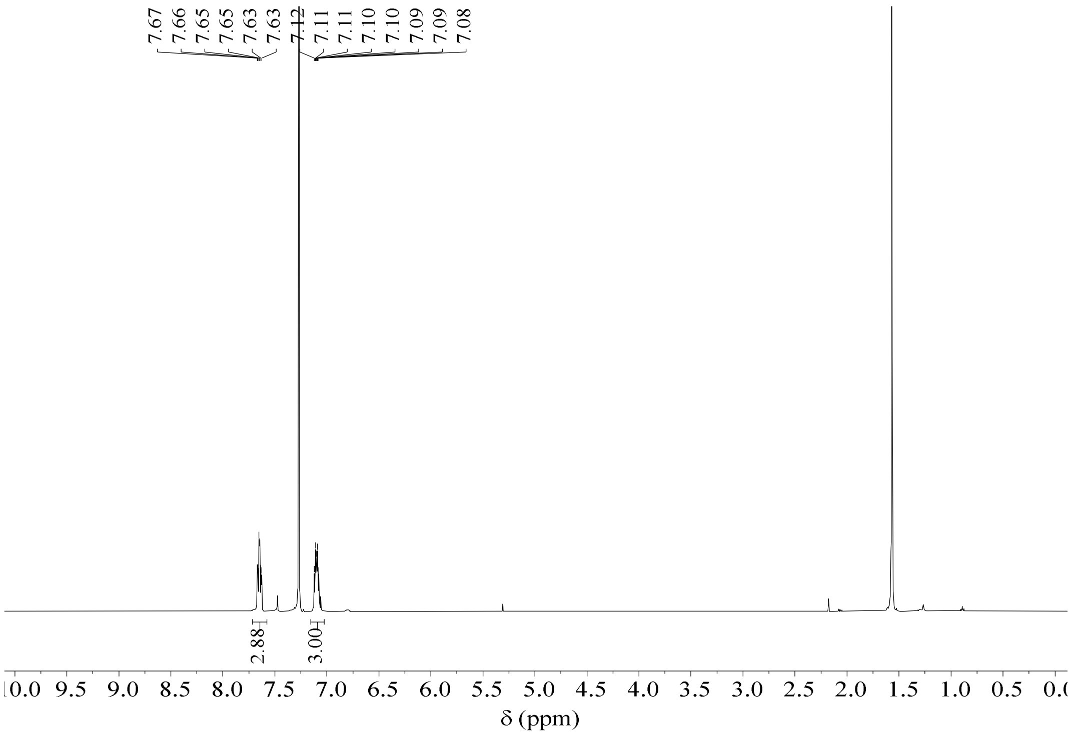

Fig. S50 ^{1}H NMR spectrum of **12** in $CDCl_3$.

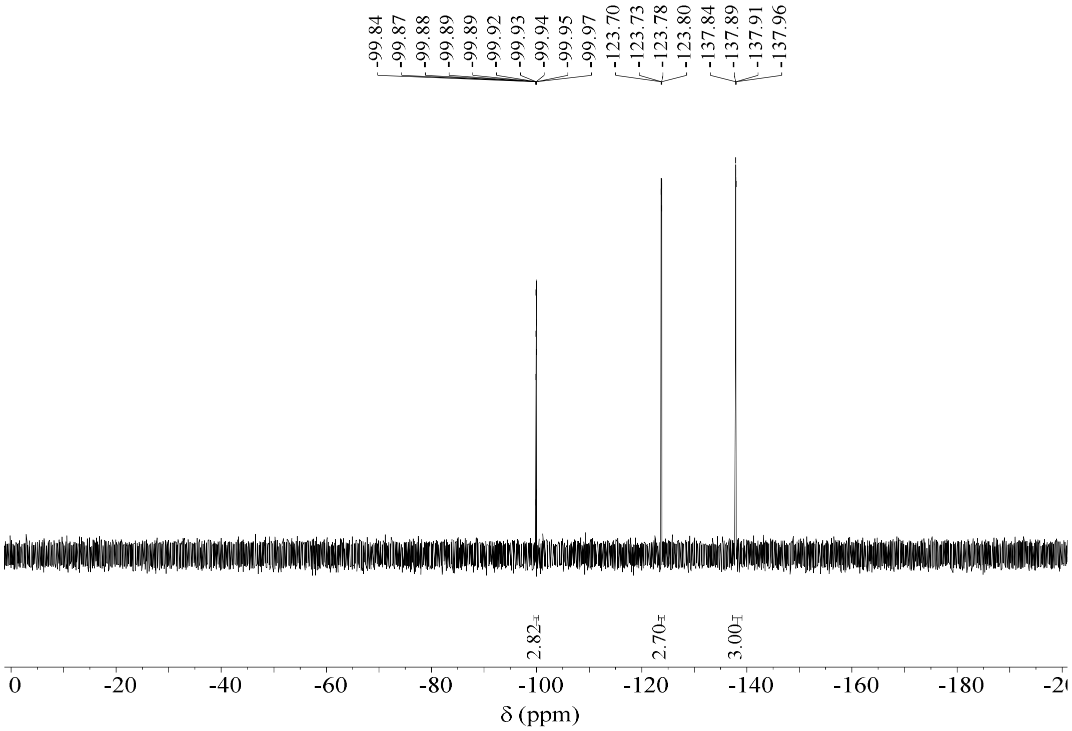

Fig. S51 ^{19}F NMR spectrum of **12** in $CDCl_3$.

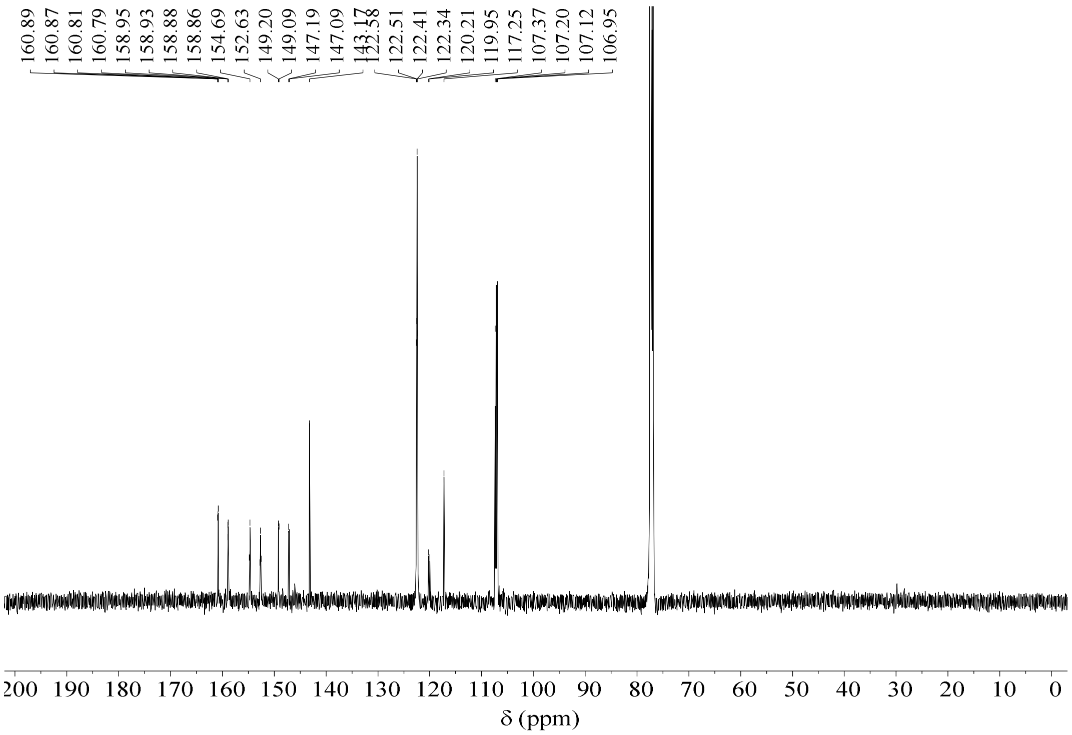

Fig. S52 ^{13}C NMR spectrum of **12** in $CDCl_3$.

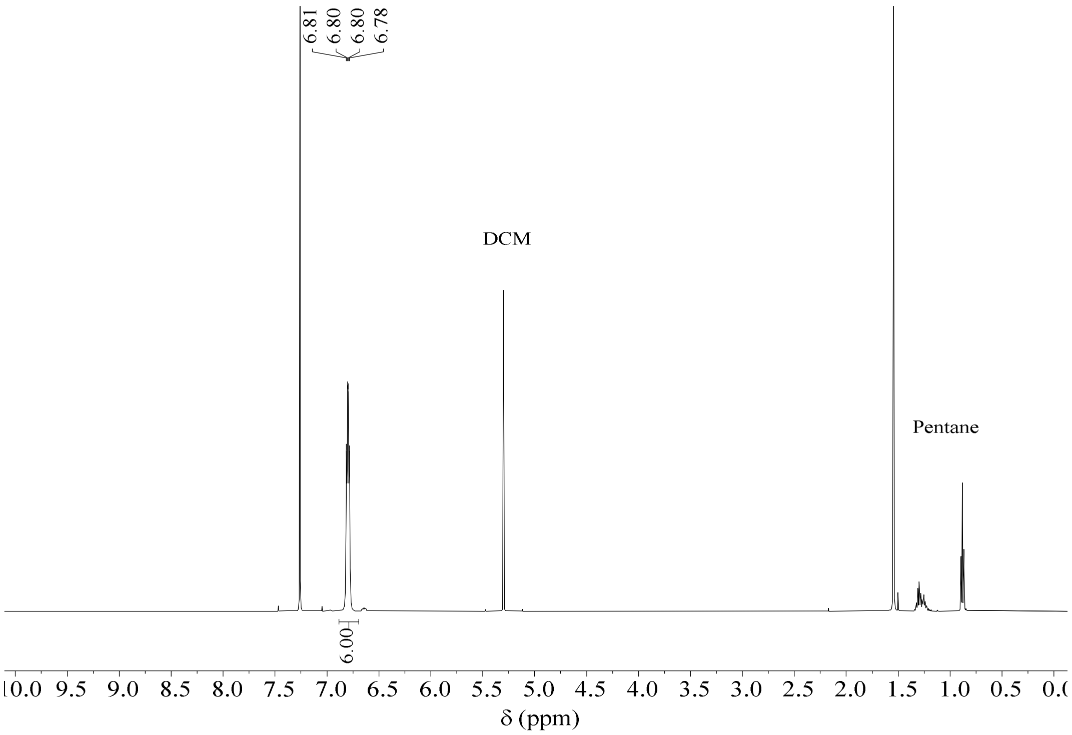

Fig. S53 ^{1}H NMR spectrum of **13** in $CDCl_3$.

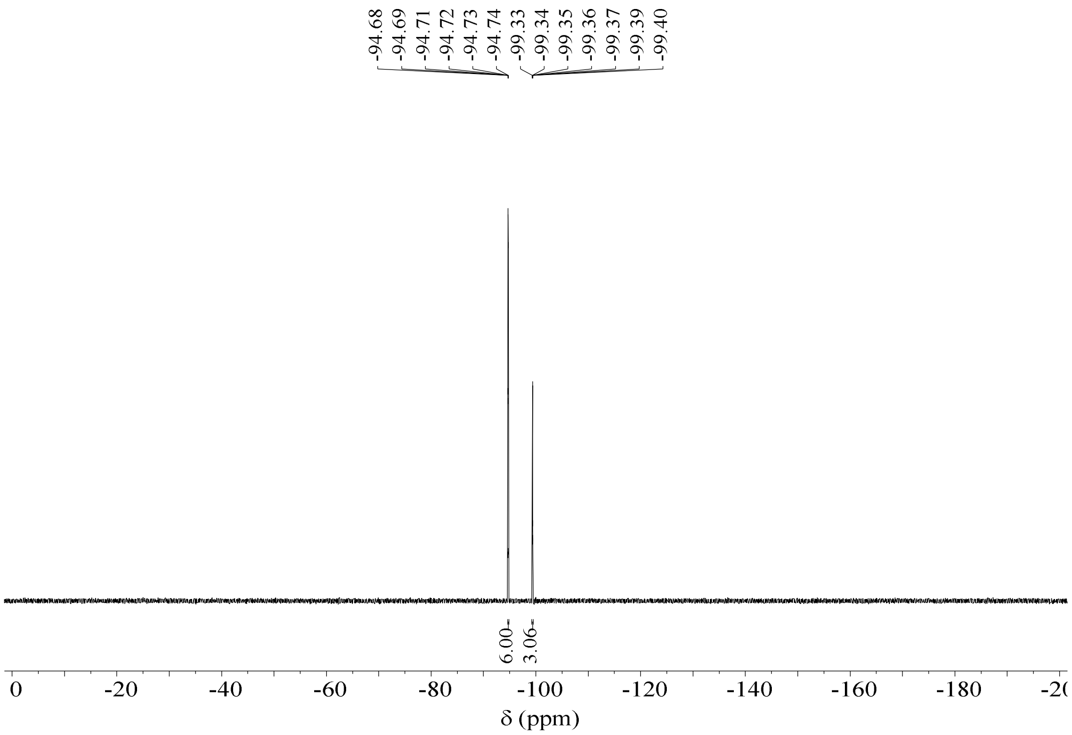

Fig. S54 ^{19}F NMR spectrum of **13** in $CDCl_3$.

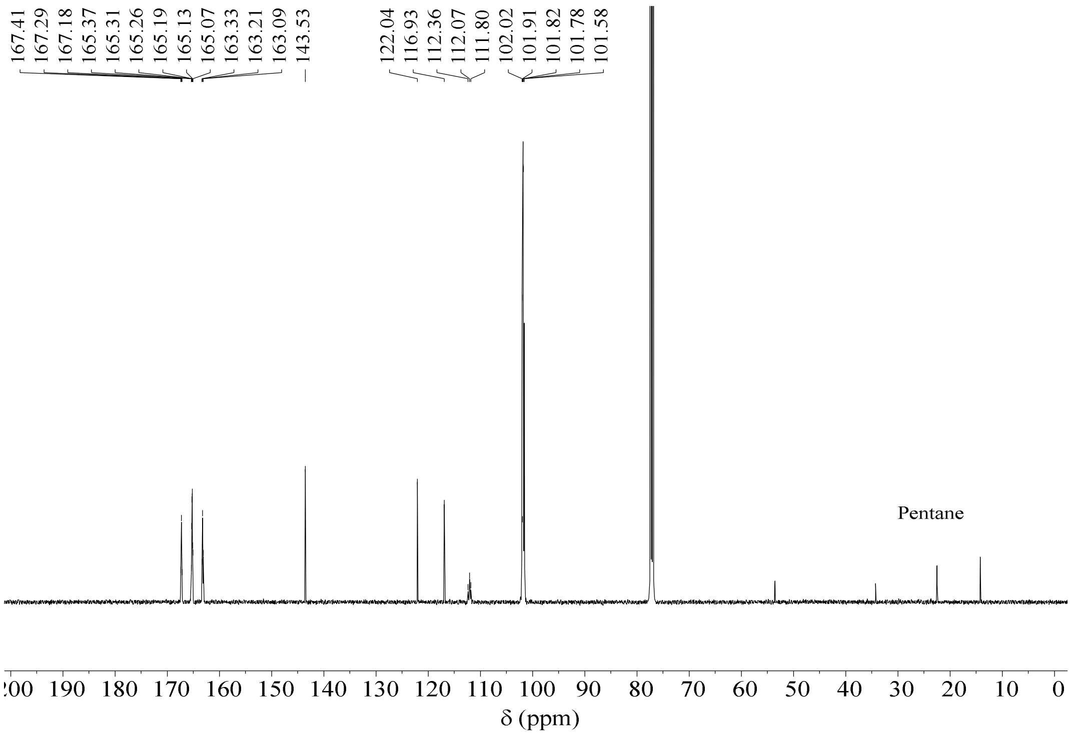

Fig. S55 ^{13}C NMR spectrum of **13** in $CDCl_3$.

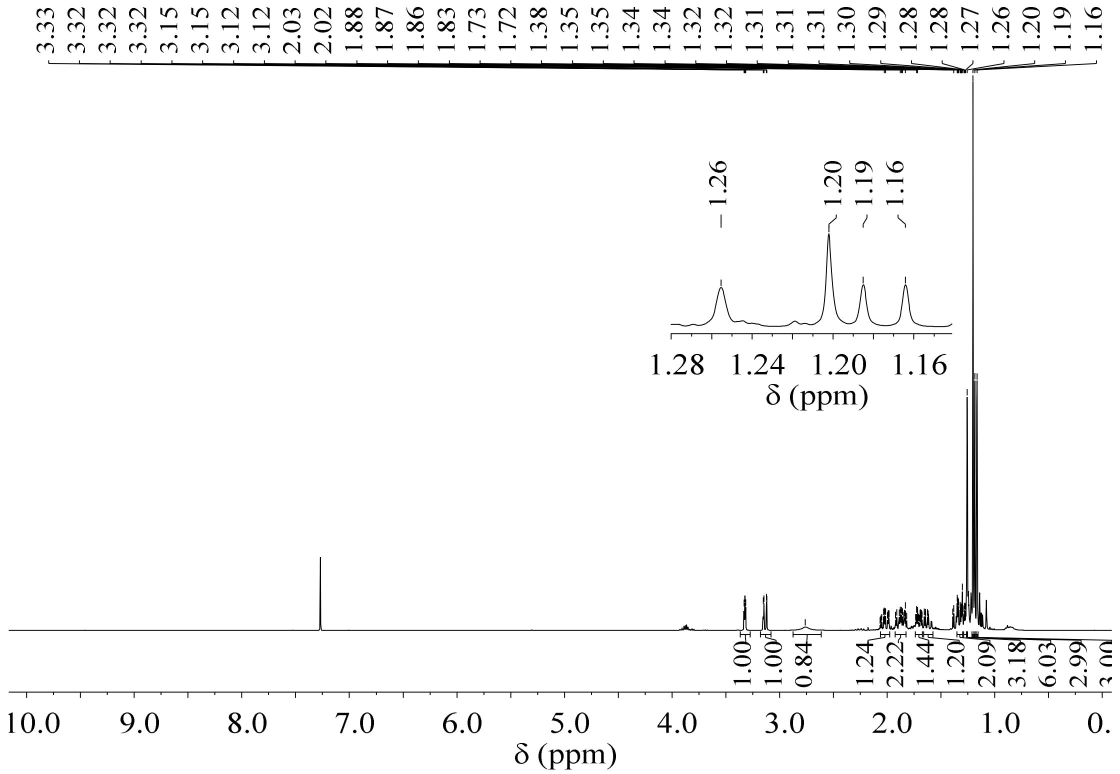

Fig. S56 ^{1}H NMR spectrum of (AB)-**32** (isomer-a) in $CDCl_3$.

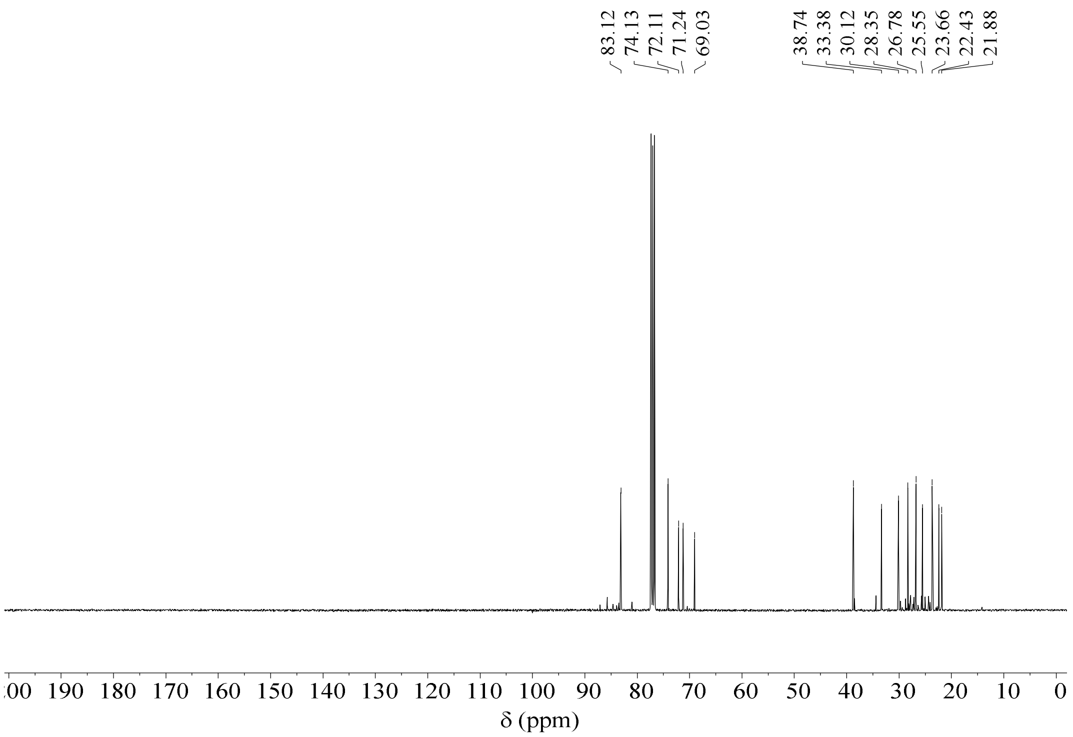

Fig. S57 ^{13}C NMR spectrum of (AB)-**32** (isomer-a) in $CDCl_3$.

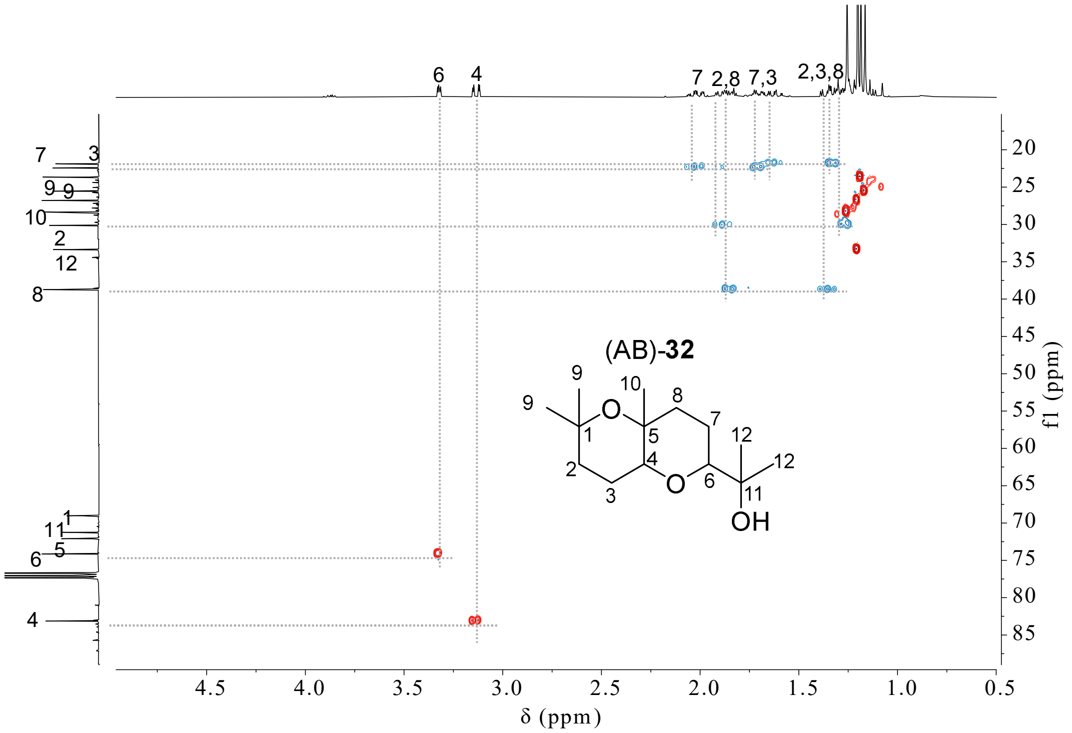

Fig. S58 HSQC spectrum of (AB)-**32** (isomer-a) in $CDCl_3$.

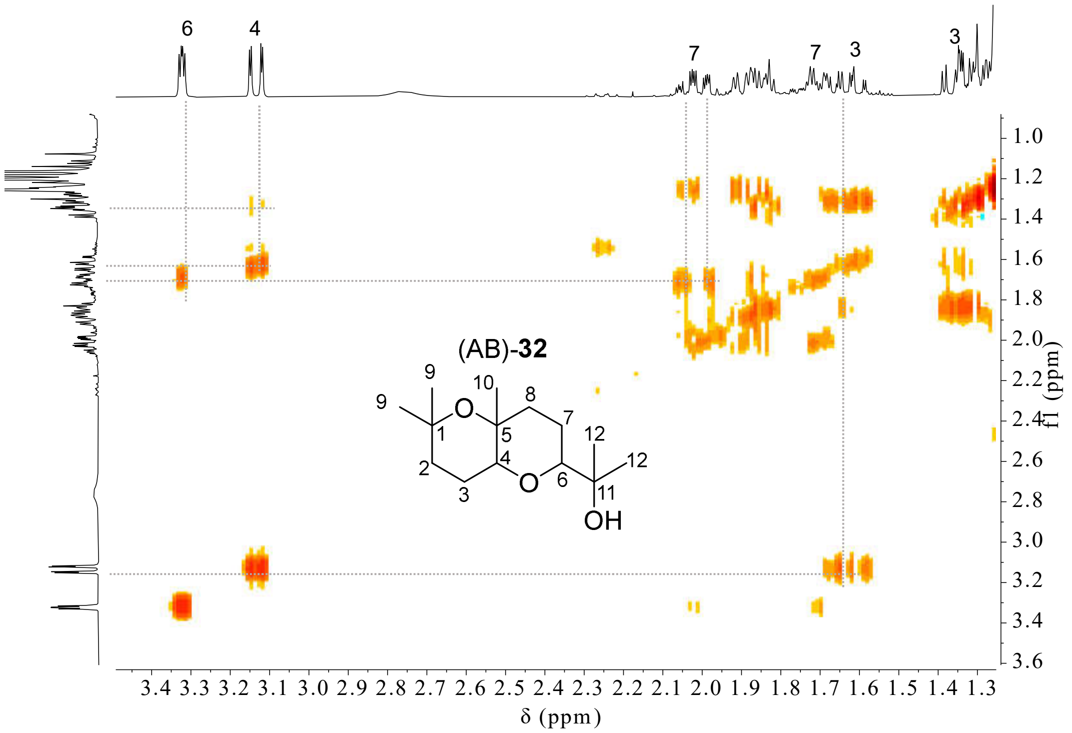

Fig. S59 COSY spectrum of (AB)-**32** (isomer-a) in $CDCl_3$.

Analysis. Four different methyl signals were observed in the ^{1}H NMR spectrum, typical for the X-B system (two methyl groups at position 12 are equivalents). Proton 6 had a typical *J*-coupling constant for eq-ax coupling in six-membered rings. The chemical shifts of protons and carbons 4 and 6 were both in the region of 6 membered ring ethers. No correlation with the reported spectra from (BB)-**30** products. No typical signal in the region for five-membered ring ethers (from 3.7 to 4.2 ppm).

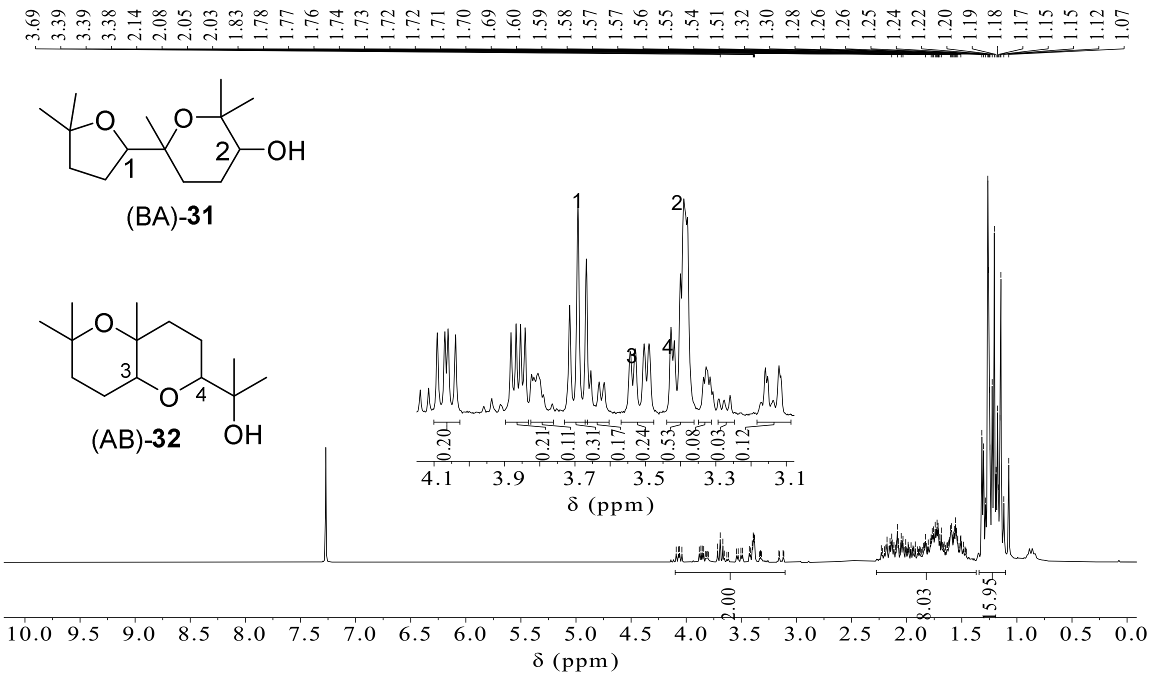

Fig. S60 ^{1}H NMR spectrum of fraction B containing **30-33** in $CDCl_3$. See Fig. S10 for assignments.

Analysis. (BA)-**31** isomer-a: Apparent large triplet peak for proton 1 is characteristic for non-fused polycyclic five-membered ring ethers. The chemical shift of proton 2 was in the region of six-membered ring ethers. No correlation with the reported spectra from (BB)-**30** products. (AB)-**32** isomer-c: Protons 3 and 4 had typical *J*-coupling constants for ax-eq coupling in six-membered rings. The chemical shifts of protons 3 and 4 were in the region of fused six-membered ring ethers, consistent with similar structures reported in reference S17.

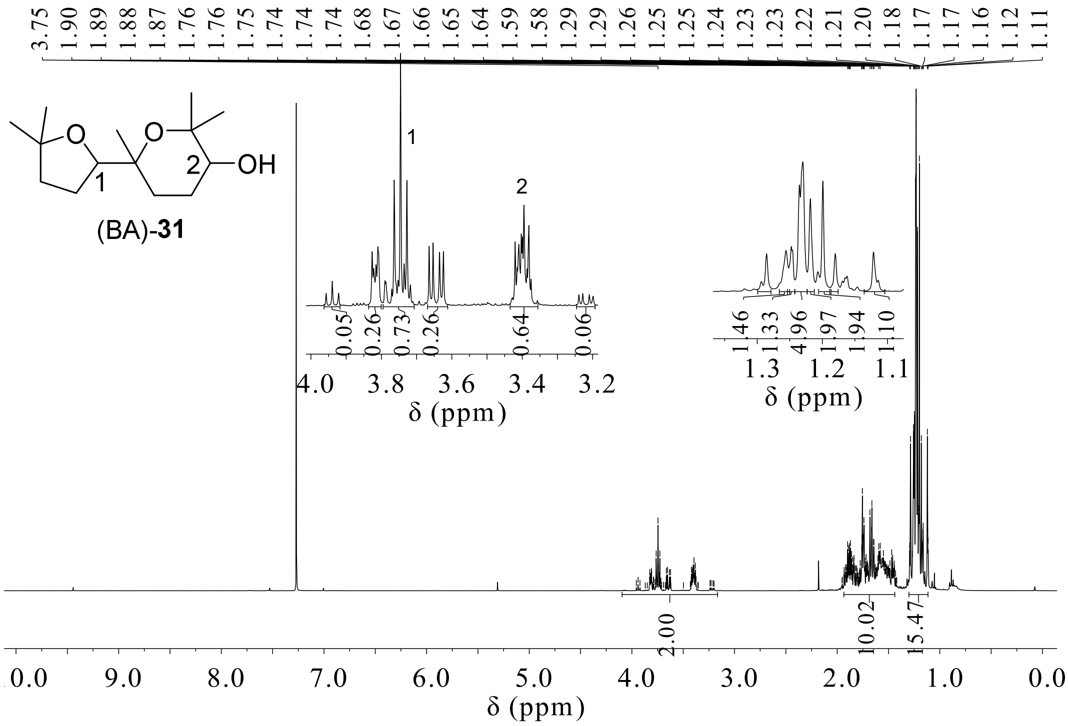

Fig. S61 ^{1}H NMR spectrum of fraction D containing **30-33** in $CDCl_3$. See Fig. S10 for assignments.

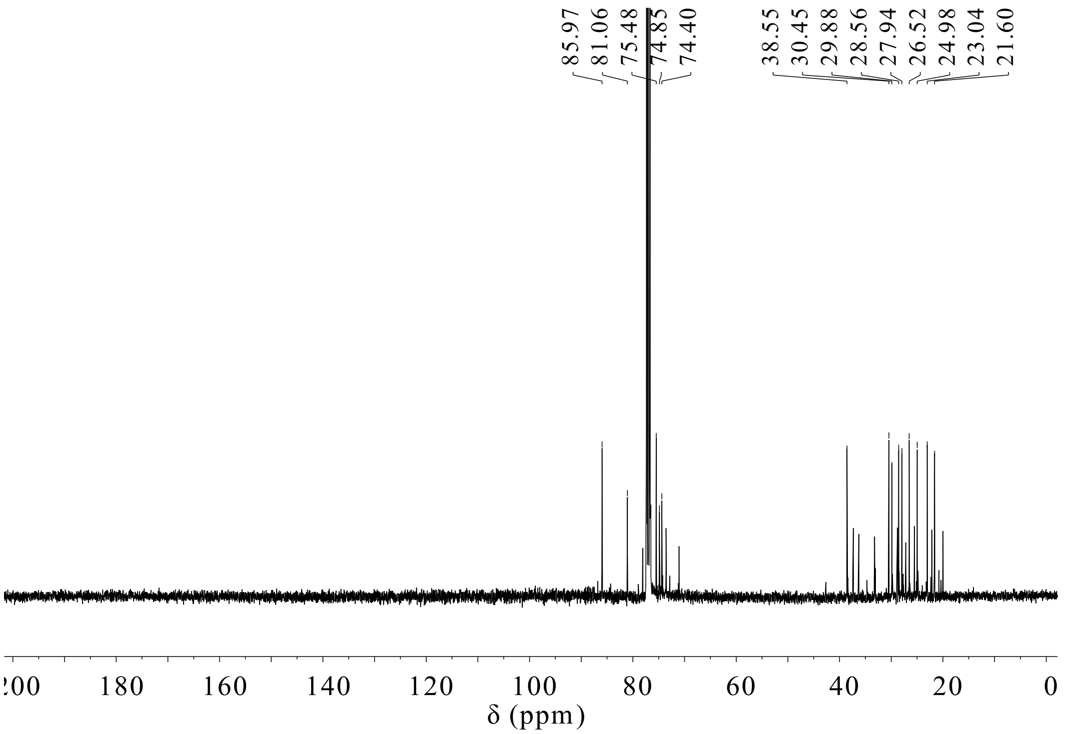

Fig. S62 ^{1}H NMR spectrum of fraction D in $CDCl_3$.

Analysis. (BA)-**31** isomer-b: Apparent large triplet peak for proton 1 was characteristic for non-fused polycyclic five-membered ring ether. The chemical shift of proton 2 is in the region of six-membered ring ethers. No correlation with the reported spectra from (BB)-**30** products. The spectra were consistent with the ones reported in reference S8.

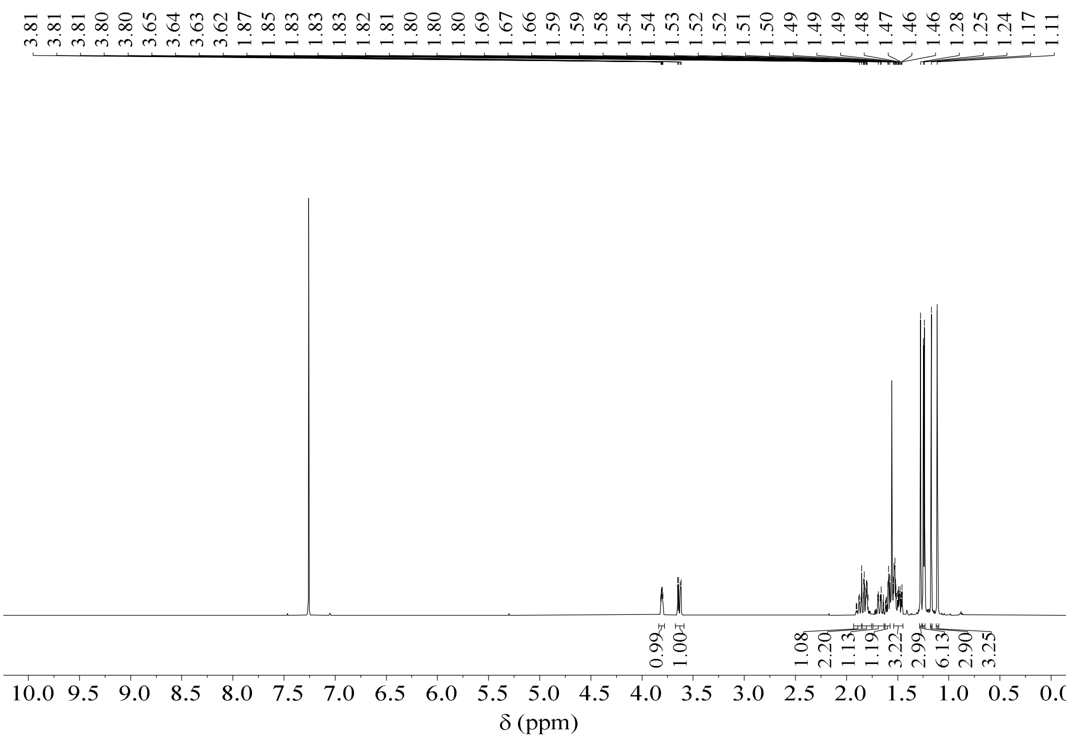

Fig. S63 ^{1}H NMR spectrum of *trans,trans* (AA)-**33** in $CDCl_3$.

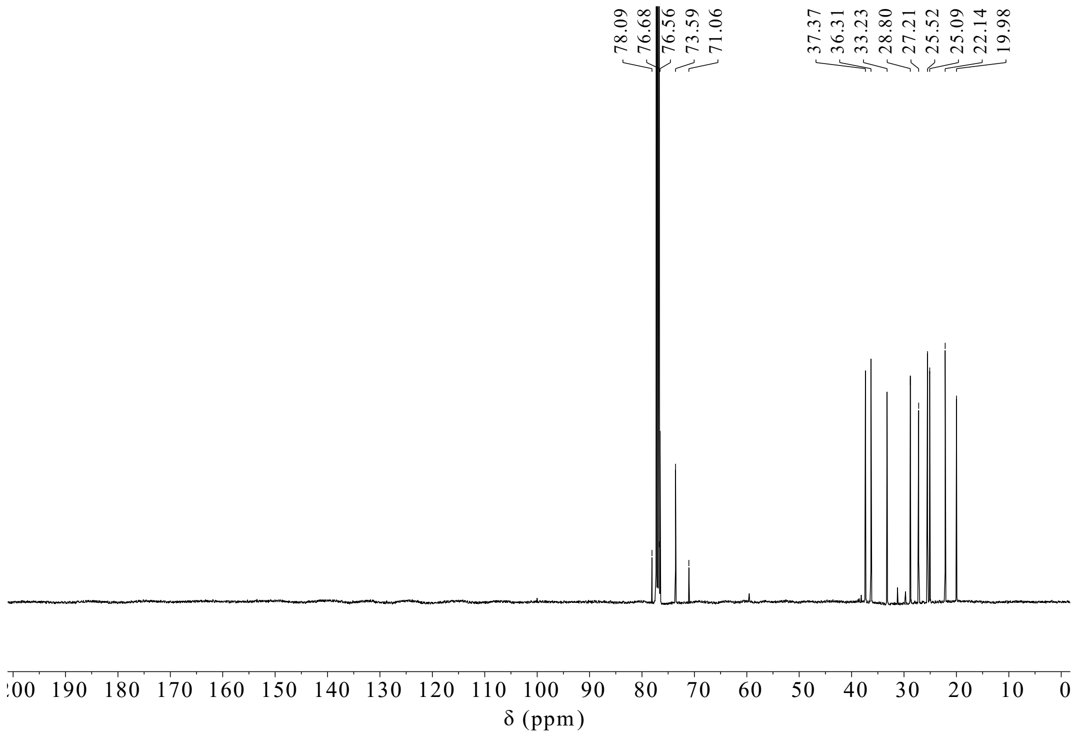

Fig. S64 ^{13}C NMR spectrum of *trans,trans* (AA)-**33** in $CDCl_3$.

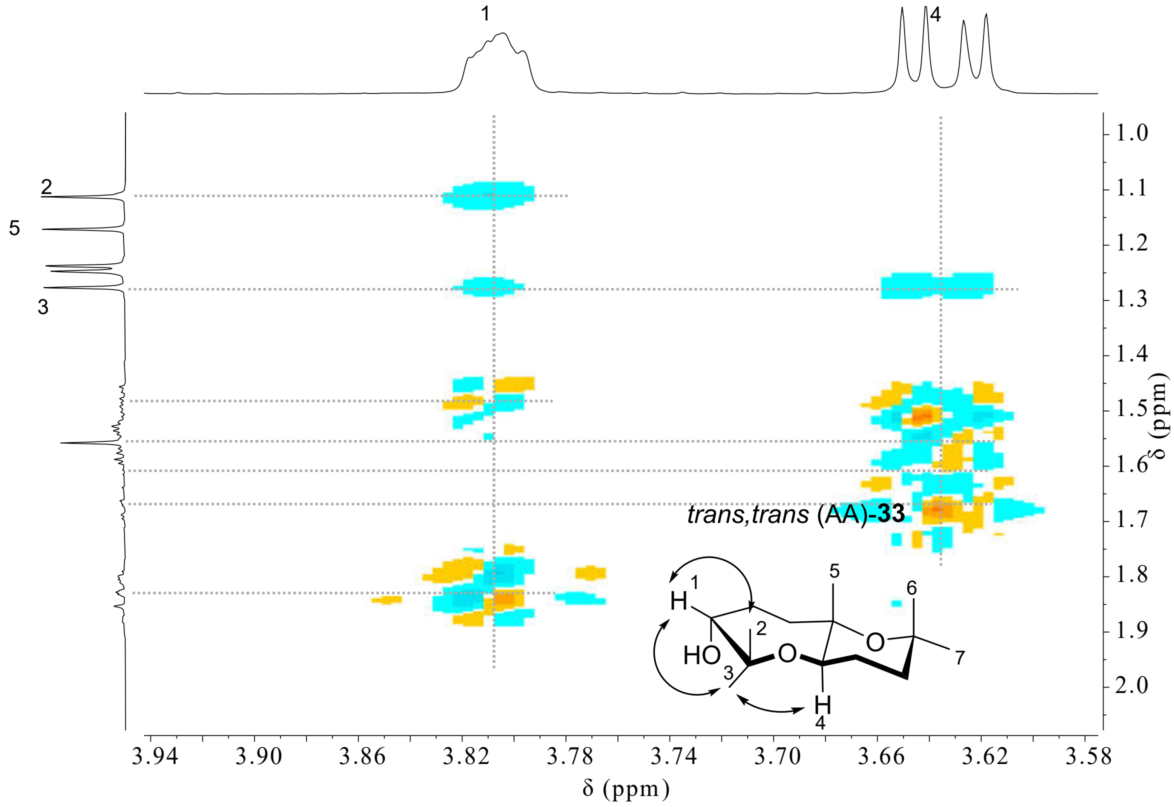

Fig. S65 NOESY NMR spectrum of *trans,trans* (AA)-**33** in $CDCl_3$.

Analysis. Five different methyl signals were observed in the ^{1}H NMR spectrum, typical for X-A systems (two methyl groups at position 2-3 are not equivalent). Proton 4 had typical *J*-coupling constants for ax-eq coupling in six-membered rings. Proton 1 did not appear as the typical apparent large triplet for five-membered ring ethers. The chemical shift of proton 4 was in the region of six-membered ring ethers. In the NOESY 2D spectrum, both protons 1 and 4 correlated to equatorial methyl 3 protons, but proton 4 did not with axial methyl 2 protons. Methyl 2 protons were on the opposite side to proton 4, thus protons 4 and 1 were on the opposite side (*trans* configuration). In addition, proton 4 did not correlate with methyl 5 protons (opposite side, *trans* configuration).

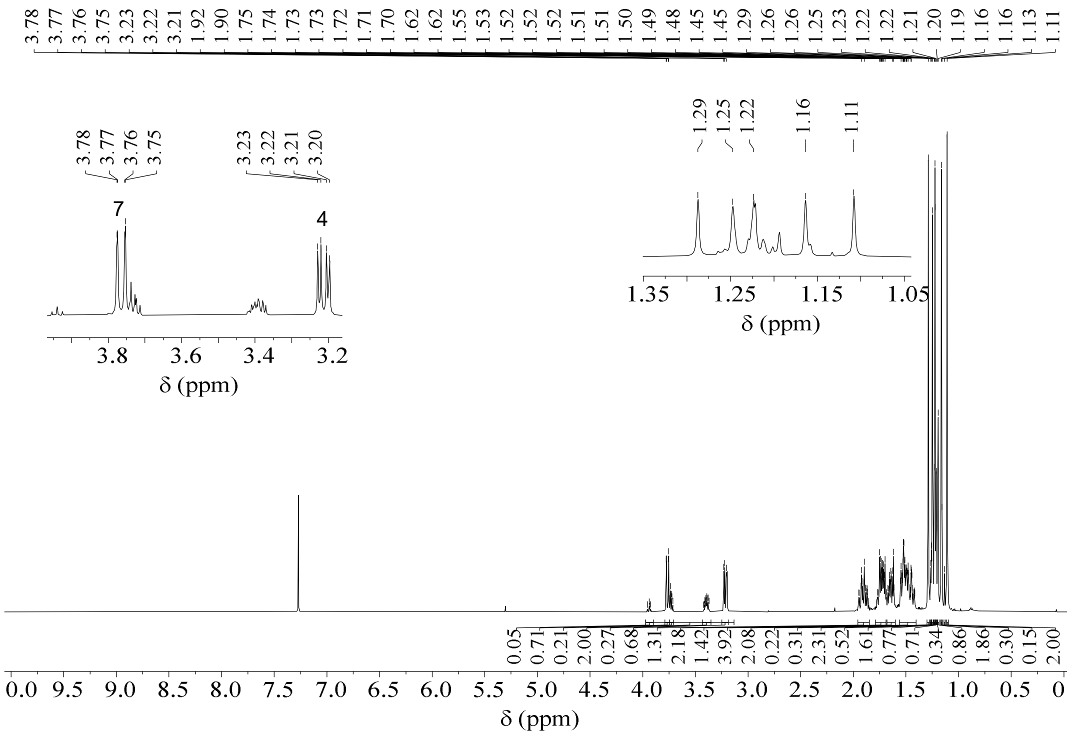

Fig. S66 ^{1}H NMR spectrum of fraction E (major product: *trans,cis* (AA)-**33** isomer) in $CDCl_3$.

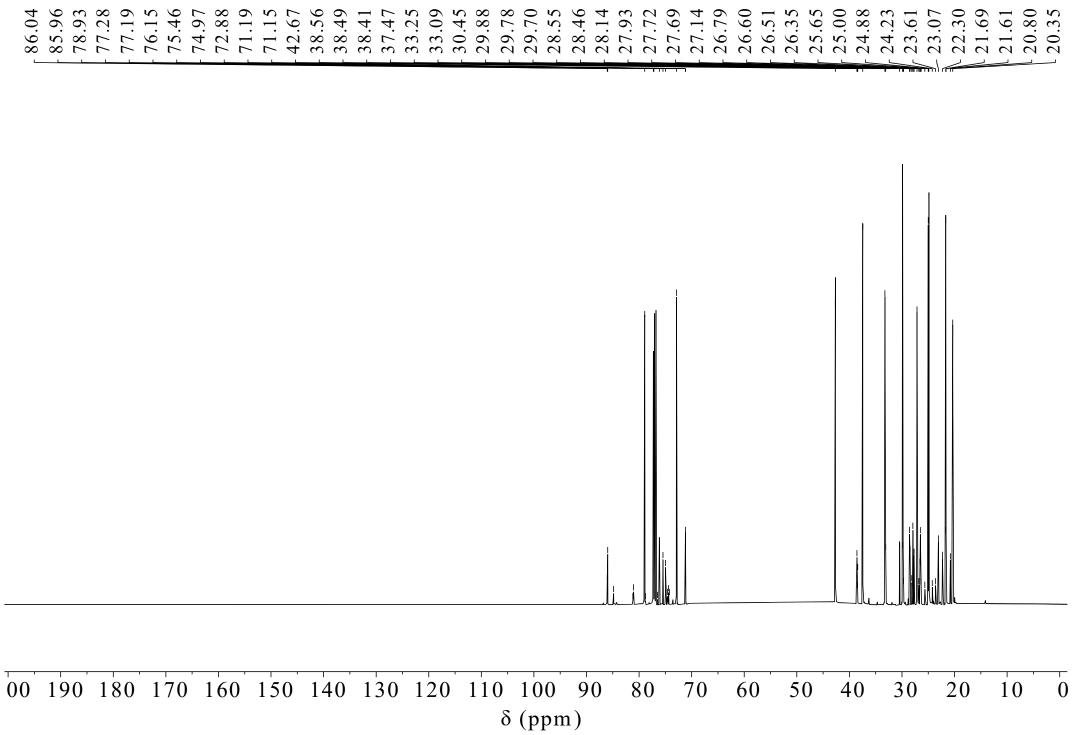

Fig. S67 ^{13}C NMR spectrum of fraction E (major product: *trans,cis* (AA)-**33** isomer) in $CDCl_3$.

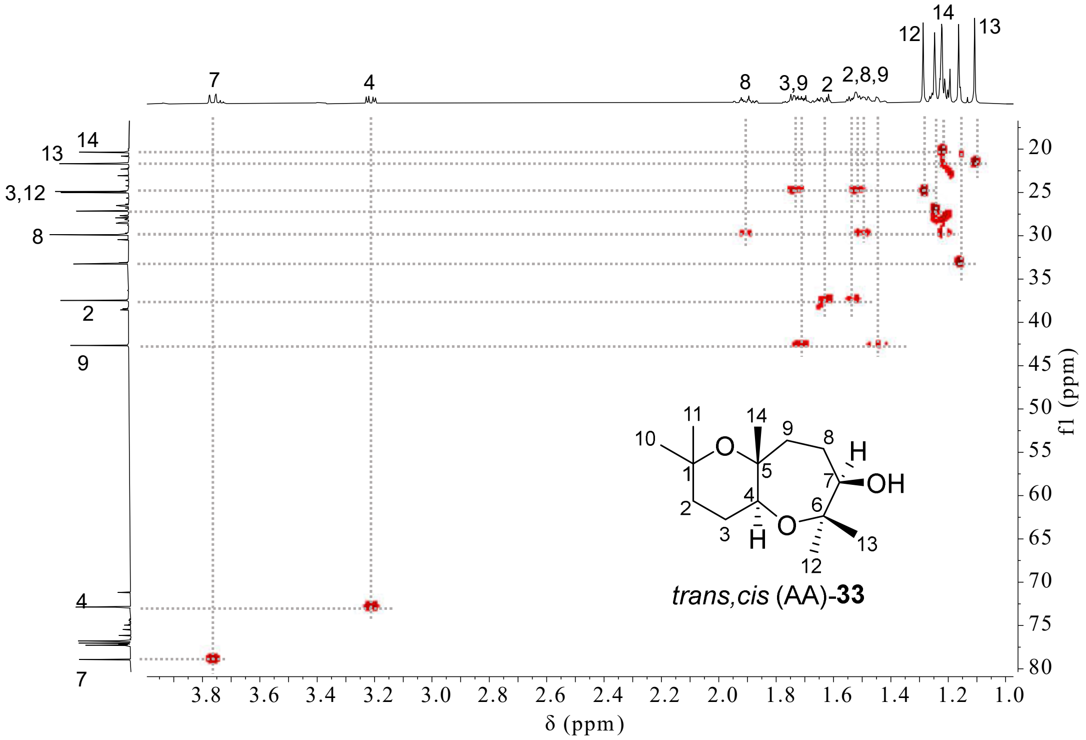

Fig. S68 HSQC NMR spectrum of fraction E (major product: *trans,cis* (AA)-**33** isomer) in $CDCl_3$.

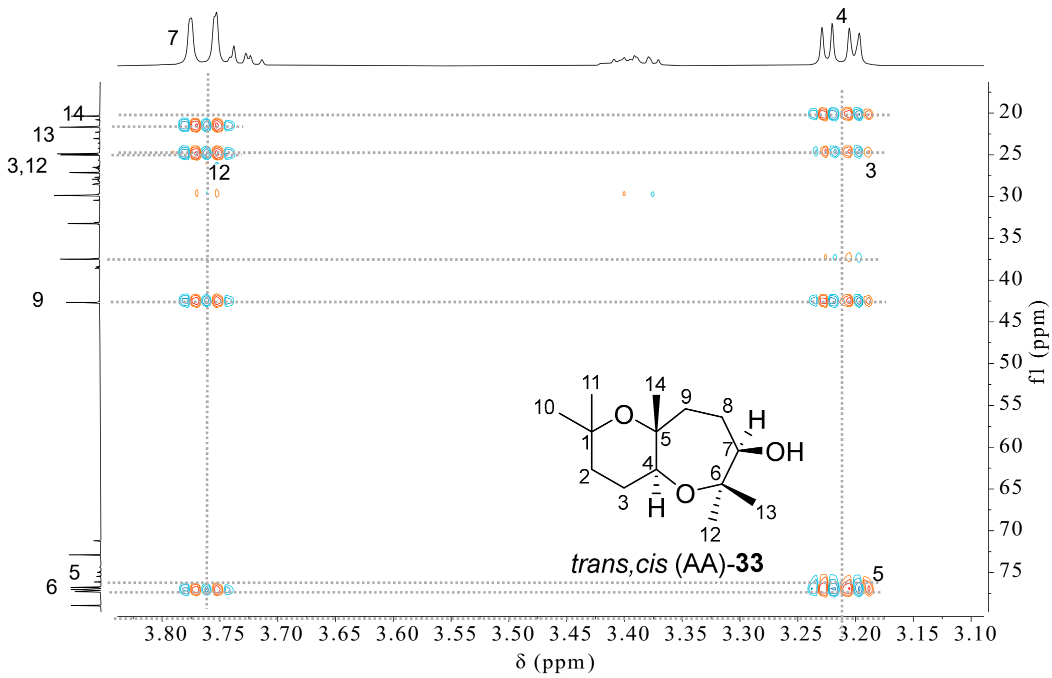

Fig. S69 HMBC NMR spectrum of fraction E (major product: *trans,cis* (AA)-**33** isomer) in $CDCl_3$.

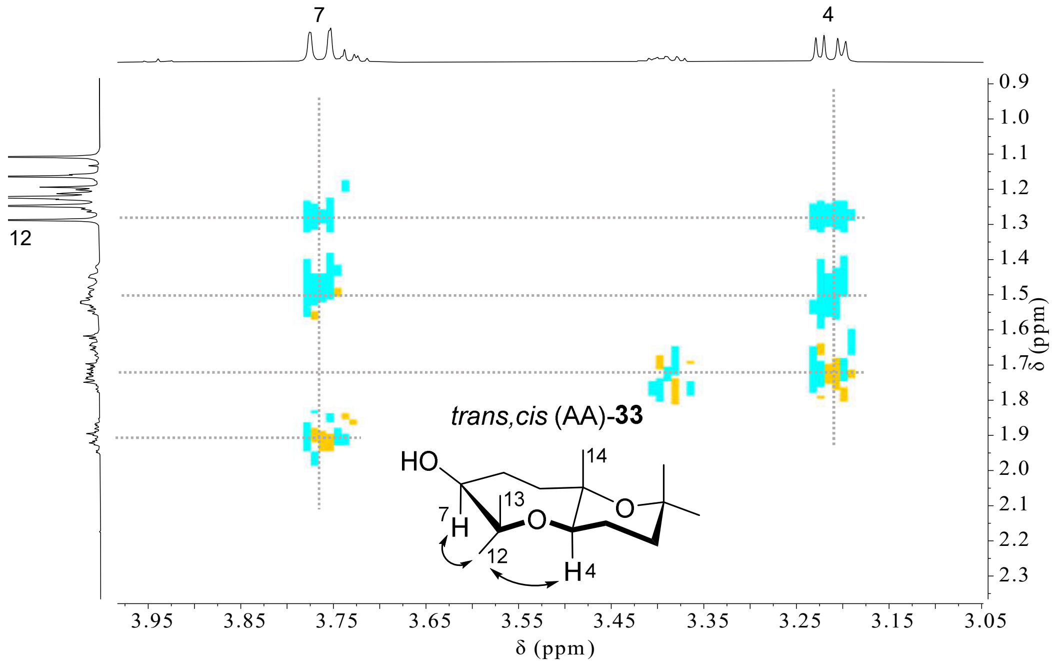

Fig. S70 NOESY NMR spectrum of fraction E (major product: *trans,cis* (AA)-**33** isomer) in $CDCl_3$.

Analysis. Five different methyl signals were observed in the ^{1}H NMR spectrum, typical for X-A systems (two methyl groups at positions 12 and 13 are not equivalent). Proton 1 did not appear as the typical apparent large triplet for five-membered ring ethers. The chemical shift of proton 4 was in the region of 6 membered ring ethers. In addition, its *J*-coupling constants were equal to the ones from the related proton in *trans,trans* (AA)-**33** (same conformation of the six-membered ring). From the HMBC spectrum, it was possible to find the correlations between the nearby methyls and protons 4 and 7 (4–14; 7–12, 13). In the NOESY 2D spectrum, both protons 7 and 4 correlated to equatorial methyl 12 protons, but not with axial methyl 13 protons. Thus, the 4 and 7 protons were on the same side (*cis* configuration). Moreover, proton 4 did not correlate with methyl 14 protons (opposite side, *trans* configuration).

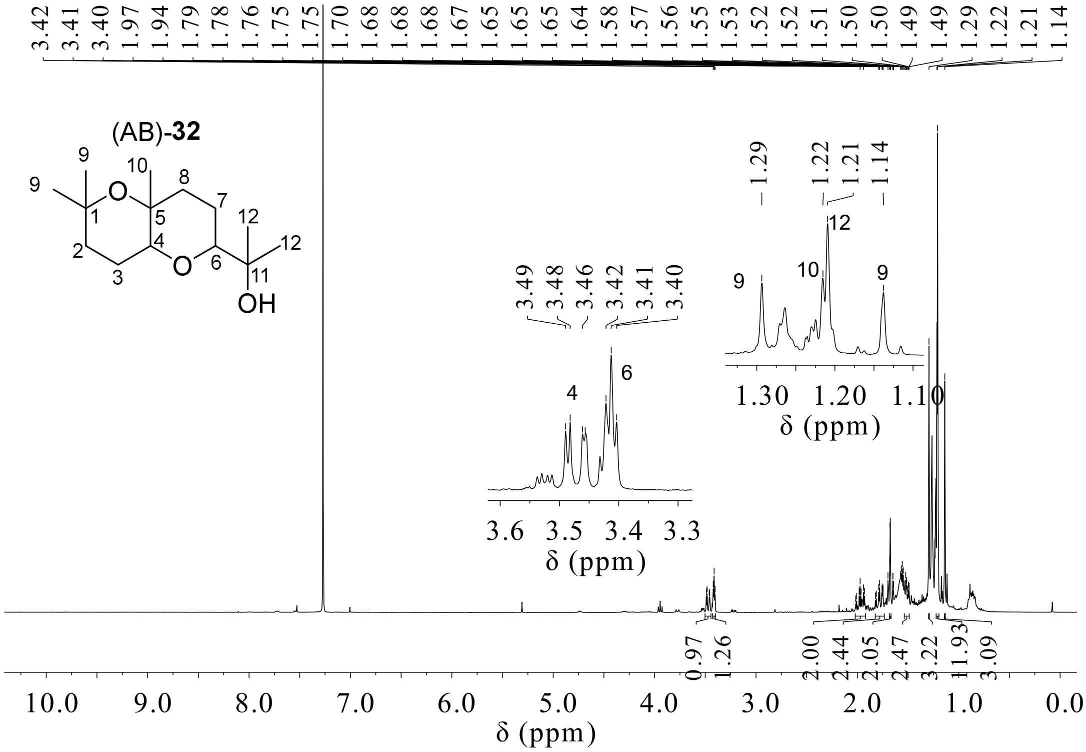

Fig. S71 ^{1}H NMR spectrum of fraction G (major product: (AB)-**32** isomer-b) in $CDCl_3$.

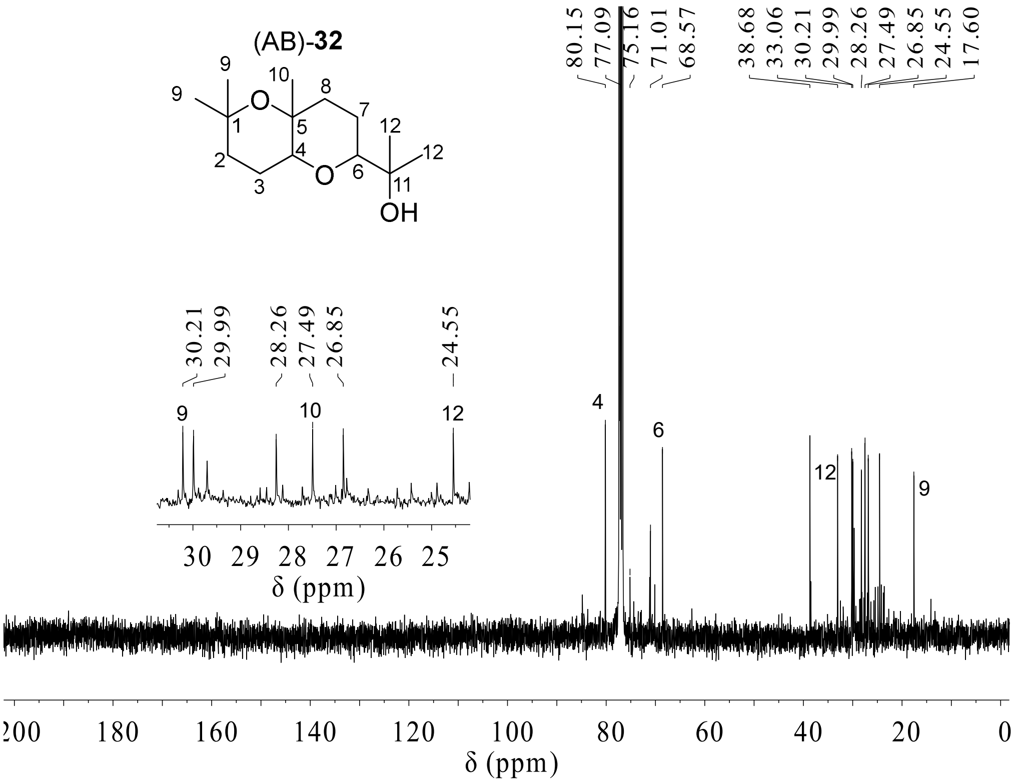

Fig. S72 ^{13}C NMR spectrum of fraction G (major product: (AB)-**32** isomer-b) in $CDCl_3$.

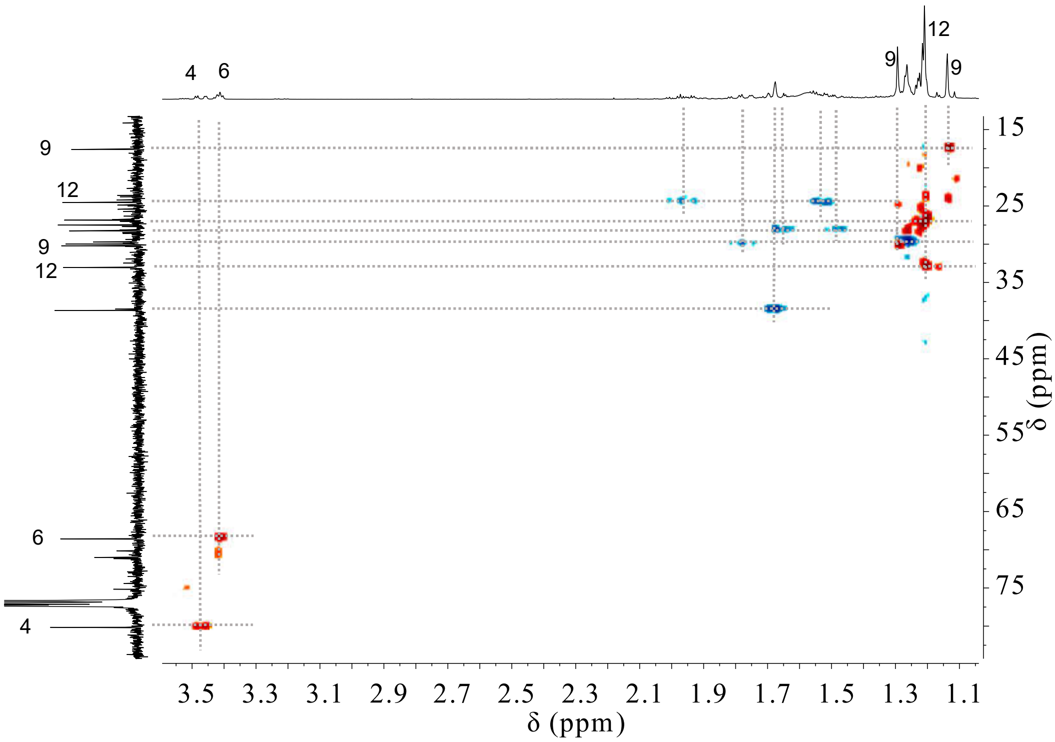

Fig. S73 HSQC NMR spectrum of fraction G (major product: (AB)-**32** isomer-b) in $CDCl_3$.

Analysis. One quaternary carbon overlapped with the solvent peak in the ^{13}C NMR spectrum. Four different methyl signals were observed in the ^{1}H NMR spectrum, typical for X-B systems (two methyl groups at position 12 are equivalent). Proton 4 had typical *J*-coupling constants for ax-eq coupling in six-membered rings. The chemical shifts of protons and carbons 4 and 6 were both in the region of six-membered ring ethers. No correlation with the reported spectra from (BB)-**30** products. No typical signals in the region for five-membered ring ethers (from 3.7 to 4.2 ppm).

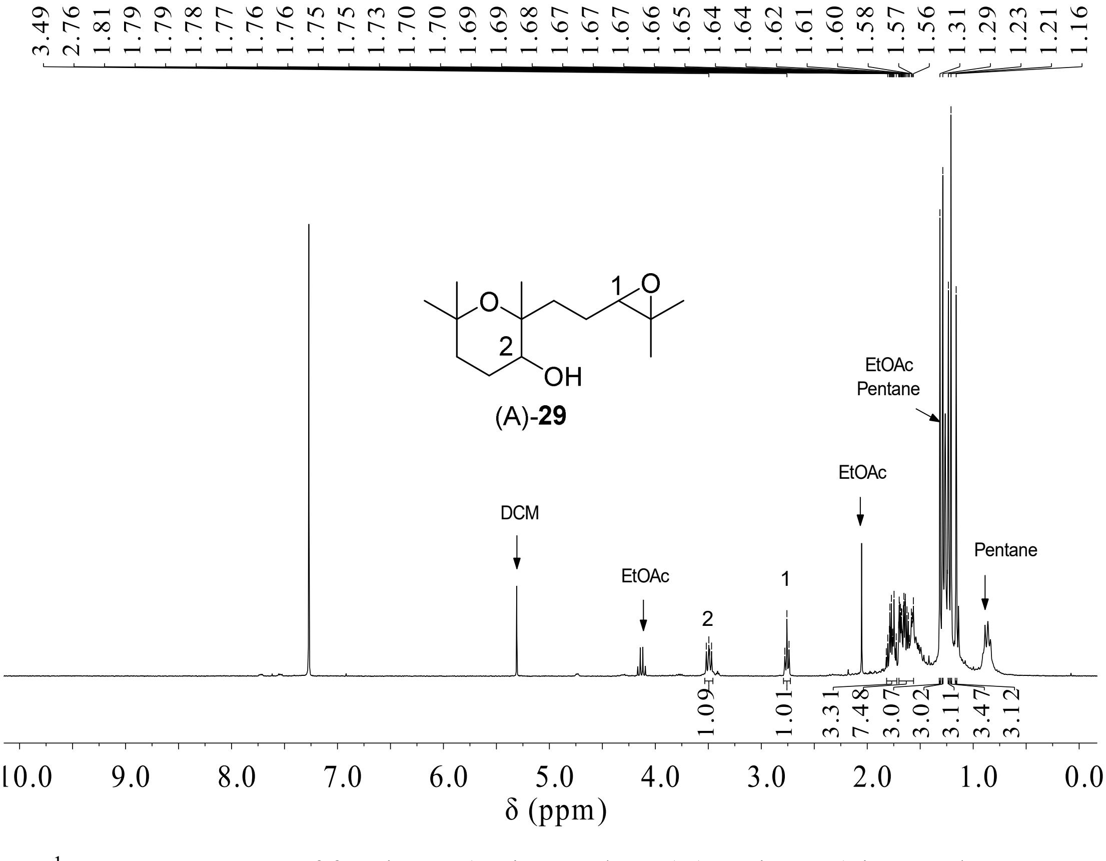

Fig. S74 ^{1}H NMR spectrum of fraction H (major product: (A)-**29** isomer) in $CDCl_3$.

Analysis. The chemical shift of proton 1 was in the epoxide region and proton 2 in the six-membered ring ethers. No typical signal in the region for five-membered ring ethers (from 3.7 to 4.2 ppm).

9. X-Ray Crystallography

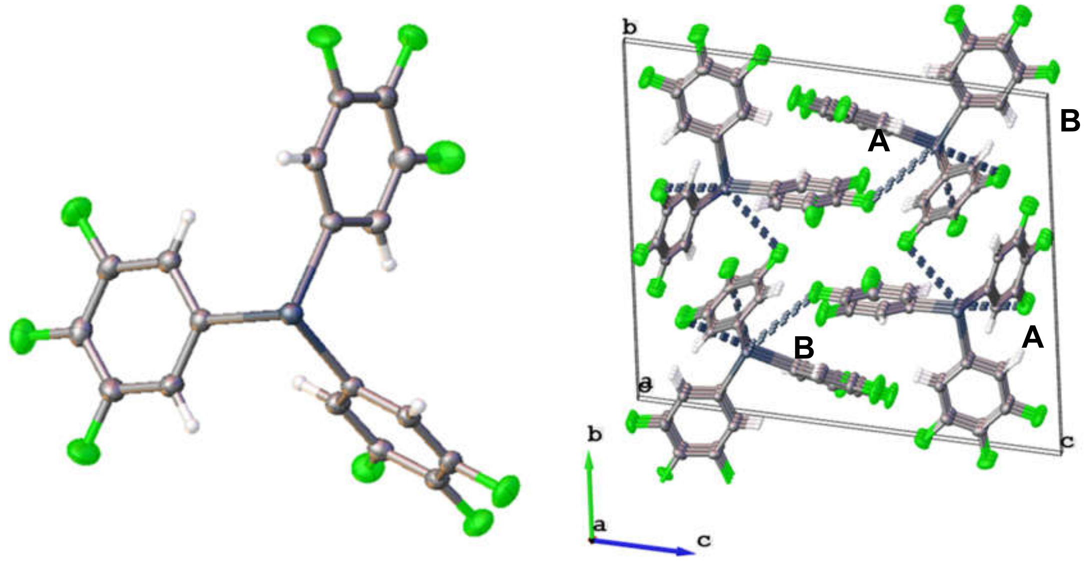

Fig. S75 Top view (left) and packed view (right) of the crystal structure for catalyst **1**. Two independent molecules (labelled A and B) are present in the asymmetric unit. Columns of molecule A and columns of molecule B are formed running along $\vec{a}$. Intermolecular short Sb-F contacts are found along with the columns and between near columns.

Table S12 Crystallographic data of **1** (CCDC 1999319)

Formula	$C_{18}H_6F_9Sb$	V (Å^3)	1689.55(4)
Space Group	P-1	Z	4
a (Å)	7.11145(10)	ρ_{calc} (g/cm^3)	2.288
b (Å)	20.3604(2)	F_{000}	1176.0
c (Å)	16.9641(2)	T (K)	140
α (°)	98.5536(12)	Reflection collected	38775
β (°)	93.9179(11)	R_1 ($I > 2.00\sigma(I)$)	0.0200
γ (°)	102.0249(12)	wR_2 (all data)	0.0495

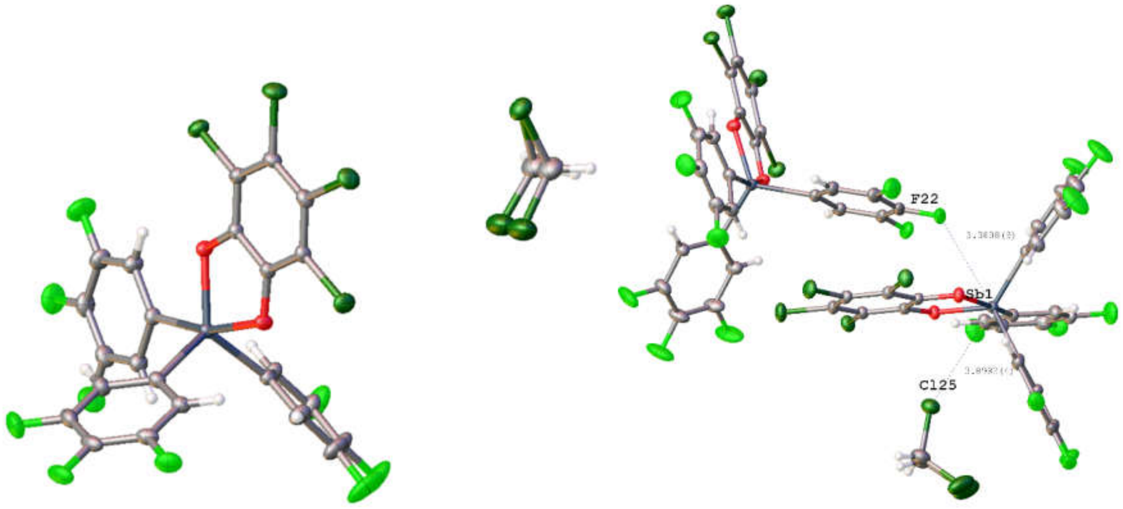

Fig. S76 Molecular view (left) and intermolecular contact (right) of the crystal structure for catalyst **2**. Selected intramolecular interaction: Sb1-F22 contact length, 3.38 Å.

Table S13 Crystallographic data of **2** (CCDC 1999325)

Formula	$C_{25}H_2Cl_6F_{15}O_2Sb$	V (Å^3)	2805.39(7)
Space Group	$P2_1/n$	Z	4
a (Å)	9.96666(13)	ρ_{calc} (g/cm^3)	2.002
b (Å)	16.7568(2)	F_{000}	1632.0
c (Å)	17.1842(2)	T (K)	150
α (°)	90	Reflection collected	61980
β (°)	102.1741(13)	R_1 ($I > 2.00\sigma(I)$)	0.0245
γ (°)	90	wR_2 (all data)	0.0581

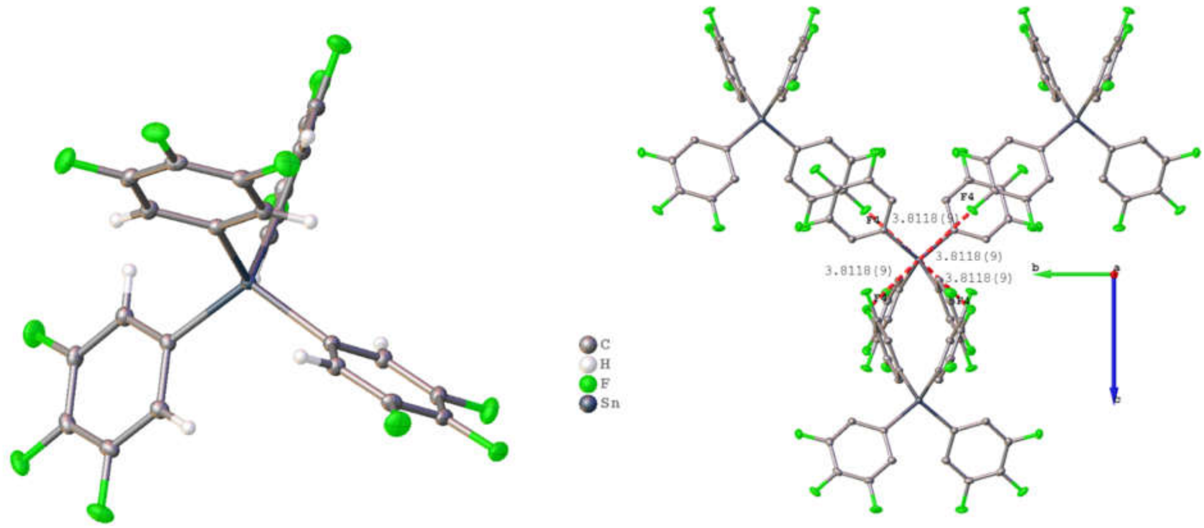

Fig. S77 Molecular view (left) and intermolecular network lateral view (right) of the crystal structure for catalyst **3**. The four intermolecular interactions are shown in dotted red line. Sn-F4 distance: 3.81 Å.

Table S14 Crystallographic data of **3** (CCDC 1999316)

Formula	$C_{24}H_8F_{12}Sn$	V (Å^3)	4271.7(2)
Space Group	$I4_1/acd$	Z	8
a (Å)	13.3697(3)	ρ_{calc} (g/cm^3)	2.000
b (Å)	13.3697(3)	F_{000}	2480.0
c (Å)	23.8975(8)	T (K)	100
α (°)	90	Reflection collected	8499
β (°)	90	R_1 ($I > 2.00\sigma(I)$)	0.0247
γ (°)	90	wR_2 (all data)	0.0731

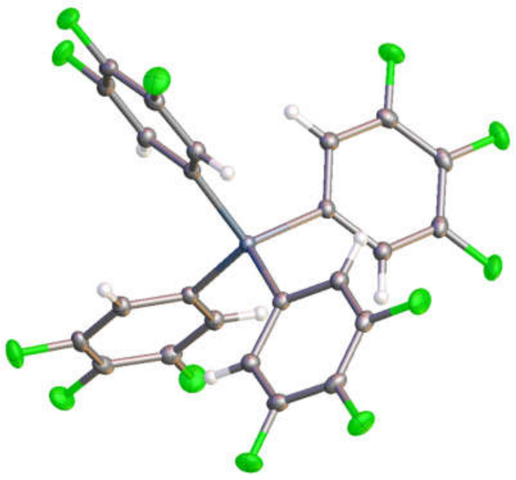

Fig. S78 Top view of the crystal structure for catalyst **4**. No intermolecular Ge-F interaction is shorter than 4.38 Å.

Table S15 Crystallographic data of **4** (CCDC 1999317)

Formula	$C_{24}H_8F_{12}Ge$	V (Å^3)	4352.32(12)
Space Group	$I4_1/acd$	Z	8
a (Å)	14.40851(16)	ρ_{calc} (g/cm^3)	1.822
b (Å)	14.40851(16)	F_{000}	2336.0
c (Å)	20.9644(4)	T (K)	100
α (°)	90	Reflection collected	17294
β (°)	90	R_1 $(I > 2.00\sigma(I))$	0.0274
γ (°)	90	wR_2 (all data)	0.0676

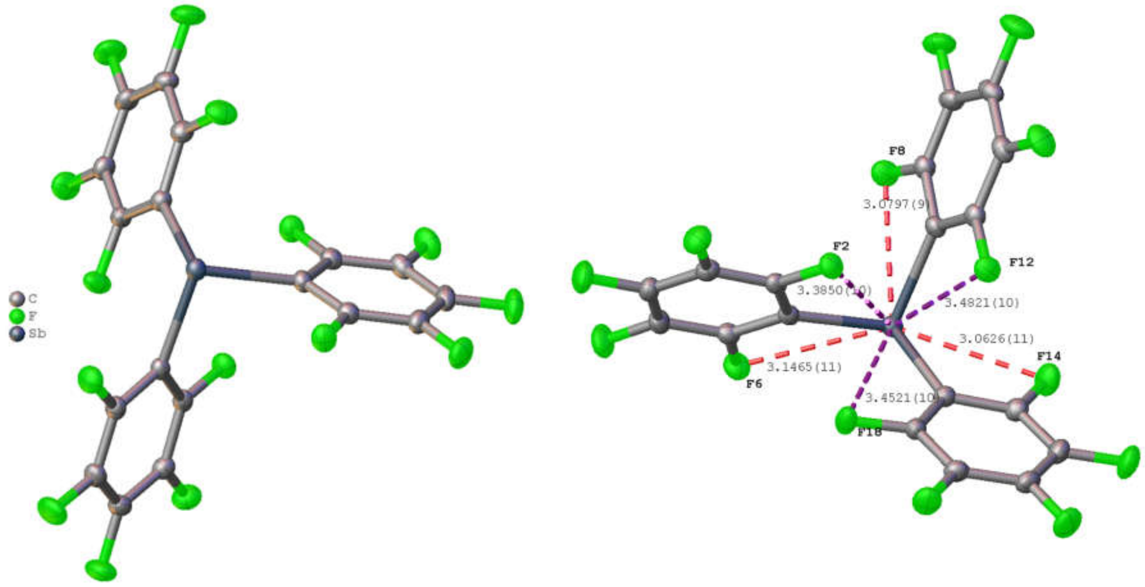

Fig. S79 Molecular view (left) of the crystal structure for catalyst **5**. In the right picture, intramolecular Sb-F interactions are shown (red shorter, purple longer). Selected distances: Sb-F6, 3.15 Å; Sb-F8, 3.08 Å; Sb-F14, 3.06 Å. The structure was already reported in reference S18.

Table S16 Crystallographic data of **5** (CCDC 1999322)

Formula	$C_{18}F_{15}Sb$	V (Å^3)	1808.52(4)
Space Group	$P2_1/n$	Z	4
a (Å)	5.93250(10)	ρ_{calc} (g/cm^3)	2.288
b (Å)	20.3604(2)	F_{000}	1176.0
c (Å)	14.9916(2)	T (K)	140
α (°)	90	Reflection collected	36719
β (°)	92.8820(10)	R_1 ($I > 2.00\sigma(I)$)	0.0220
γ (°)	90	wR_2 (all data)	0.0475

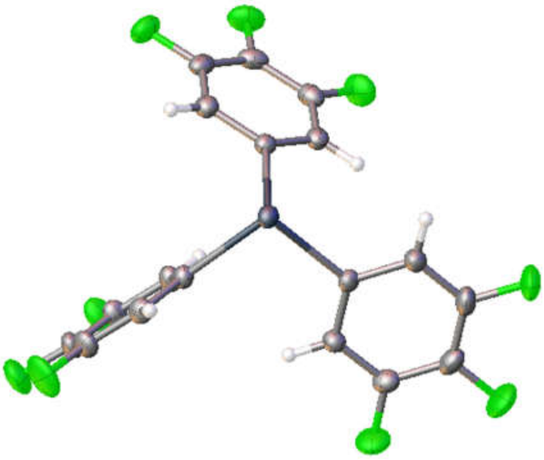

Fig. S80 Molecular view of the crystal structure for catalyst **6**.

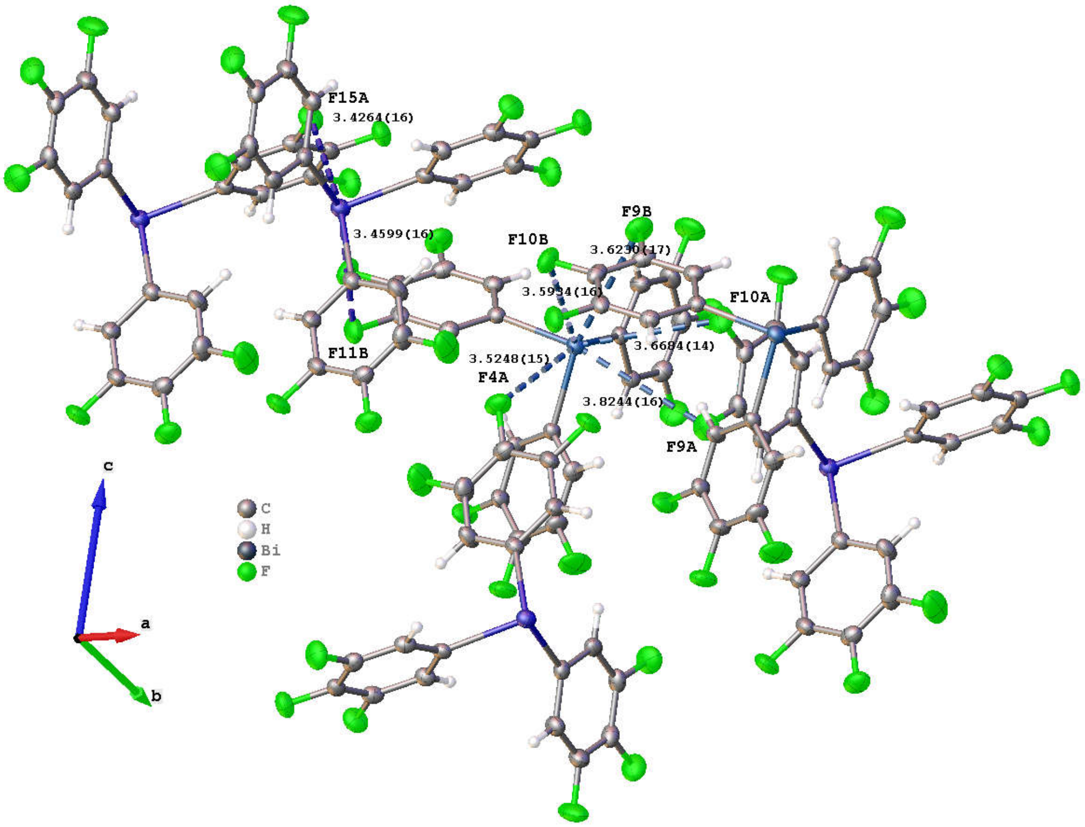

Fig. S81 Intermolecular network for catalyst **6**. Bi-F intermolecular interactions are shown in dotted blue lines.

Table S17 Crystallographic data of **6** (CCDC 199324)

Formula	$C_{18}H_6BiF_9$	V (Å^3)	1710.92(3)
Space Group	P-1	Z	4
a (Å)	7.16588(7)	ρ_{calc} (g/cm^3)	2.338
b (Å)	14.61823(16)	F_{000}	1112.0
c (Å)	17.01631(16)	T (K)	150
α (°)	98.5736(8)	Reflection collected	68969
β (°)	94.0812(8)	R_1 $(I > 2.00\sigma(I))$	0.0314
γ (°)	102.4968(8)	wR_2 (all data)	0.0535

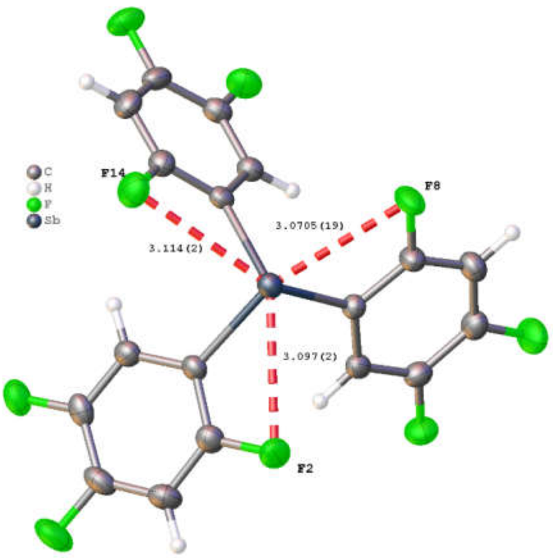

Fig. S82 Molecular view of the crystal structure for catalyst **9**. Sb-F interactions are shown in red dotted lines. Selected distance: Sb-F2, 3.10 Å; Sb-F8, 3.07 Å; Sb-F14, 3.14 Å.

Table S18 Crystallographic data of **9** (CCDC 1999320)

Formula	$C_{18}H_6F_9Sb$	V (Å^3)	812.73(3)
Space Group	P-1	Z	2
a (Å)	4.65720(10)	ρ_{calc} (g/cm^3)	2.104
b (Å)	11.8323(3)	F_{000}	492.0
c (Å)	15.3860(3)	T (K)	150
α (°)	102.271(2)	Reflection collected	19583
β (°)	90.684(2)	R_1 ($I > 2.00\sigma(I)$)	0.0308
γ (°)	100.761(2)	wR_2 (all data)	0.0857

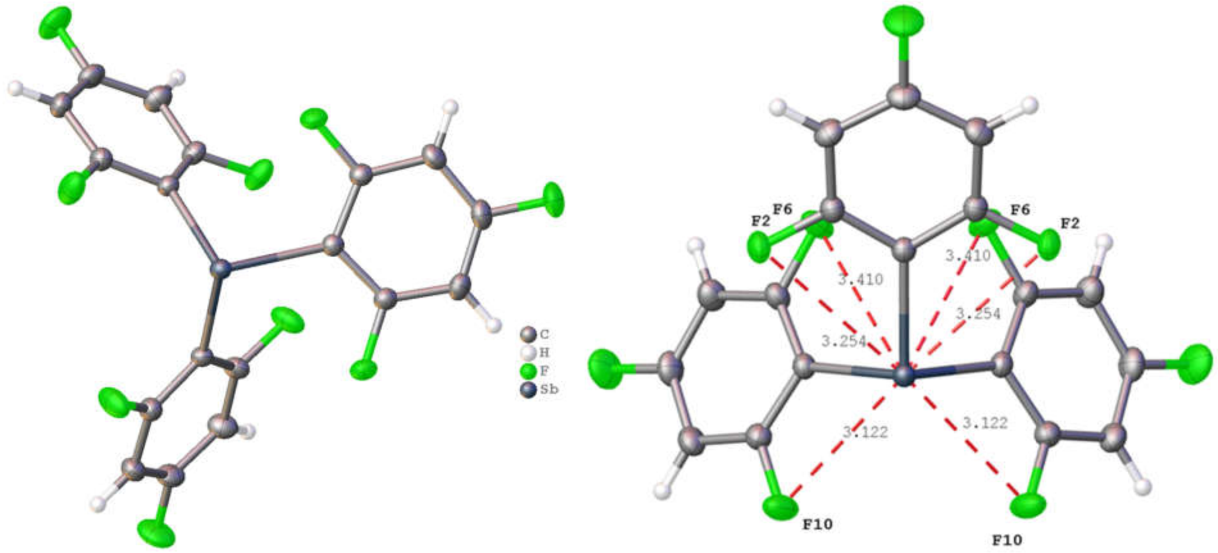

Fig. S83 Molecular view (left) and view with intramolecular interactions (right) of the crystal structure for catalyst **10**. Sb-F interactions are shown in red dotted lines. Selected distances: Sb-F2, 3.25 Å; Sb-F6, 3.34 Å; Sb-F10, 3.12 Å.

Table S19 Crystallographic data of **10** (CCDC 1999321)

Formula	$C_{18}H_6F_9Sb$	V (Å^3)	1666.33(5)
Space Group	Pnma	Z	4
a (Å)	10.2444(2)	ρ_{calc} (g/cm^3)	2.053
b (Å)	17.0576(3)	F_{000}	984.0
c (Å)	9.53580(10)	T (K)	150
α (°)	90	Reflection collected	22146
β (°)	90	R_1 ($I > 2.00\sigma(I)$)	0.0191
γ (°)	90	wR_2 (all data)	0.0495

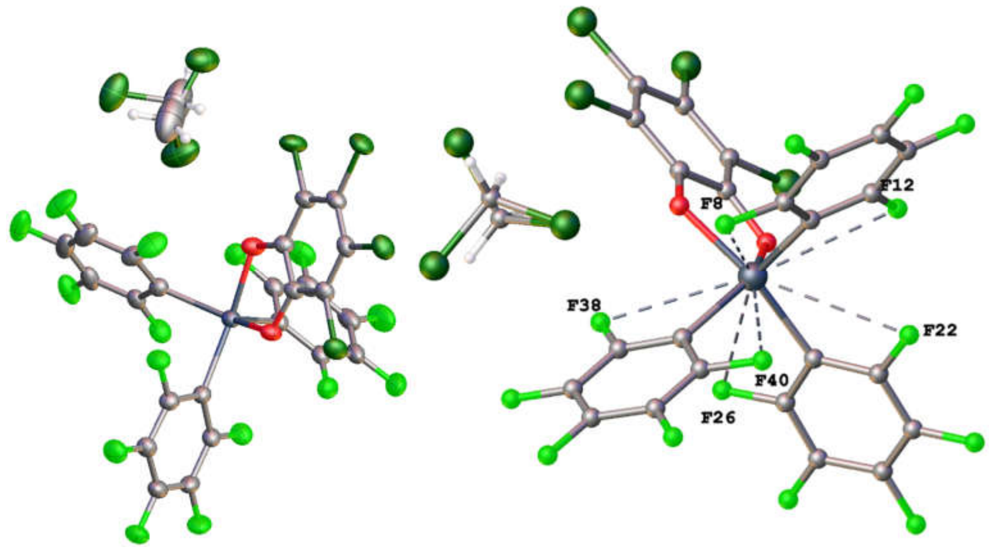

Fig. S84 Molecular view (left) and to view with intramolecular interaction (right) of the crystal structure for catalyst **11**. Sb-F interactions are shown in grey dotted lines. Selected distances: Sb-F8, 3.20 Å; Sb-F12, 3.19 Å; Sb-F26, 3.07 Å; Sb-F38, 3.24 Å; Sb-F40, 3.17 Å. The structure of the same complex crystallized with $CHCl_3$ was reported in reference S4.

Table S20 Crystallographic data of **11** (CCDC 1999323)

Formula	$C_{25}H_2Cl_6F_{15}O_2Sb$	V (Å^3)	2949.87(9)
Space Group	P2$_1$/n	Z	4
a (Å)	10.37807(18)	ρ_{calc} (g/cm^3)	2.147
b (Å)	18.8151(3)	F_{000}	1824.0
c (Å)	15.5321(2)	T (K)	150
α (°)	90	Reflection collected	51441
β (°)	103.4341(16)	R_1 ($I > 2.00\sigma(I)$)	0.0499
γ (°)	90	wR_2 (all data)	0.1409

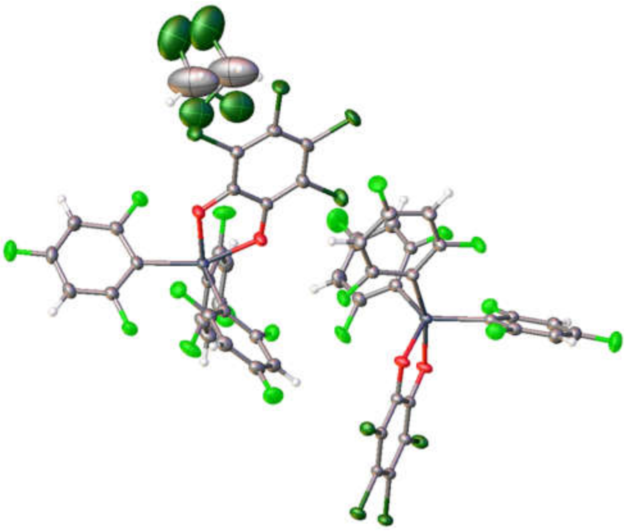

Fig. S85 View of the asymmetric unit for catalyst **13**. There are two non-symmetrically equivalent complexes per asymmetric unit.

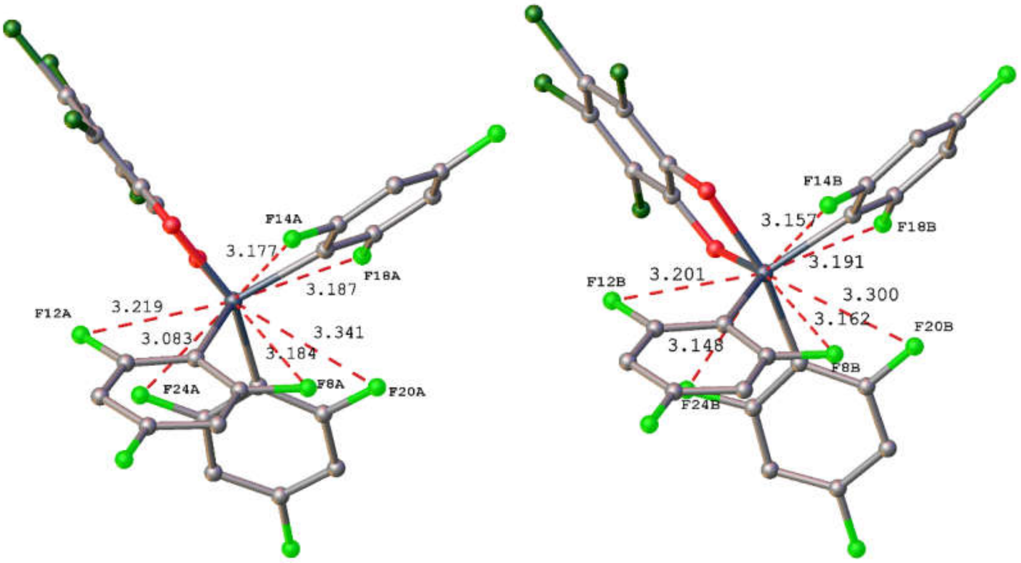

Fig. S86 Details of the two symmetrically non-equivalent complexes for **13**. Sb-F interactions are shown in red dotted lines.

Table S21 Crystallographic data of **13** (CCDC 1999326)

Formula	$C_{49}H_{14}Cl_{10}F_{18}O_4Sb_2$	V (Å^3)	2727.81(7)
Space Group	P-1	Z	2
a (Å)	11.4280(2)	ρ_{calc} (g/cm^3)	1.956
b (Å)	14.4693(2)	F_{000}	1548.0
c (Å)	17.6698(2)	T (K)	150
α (°)	103.0960(10)	Reflection collected	73727
β (°)	97.1000(10)	R_1 ($I > 2.00\sigma(I)$)	0.0305
γ (°)	102.8310(10)	wR_2 (all data)	0.0856

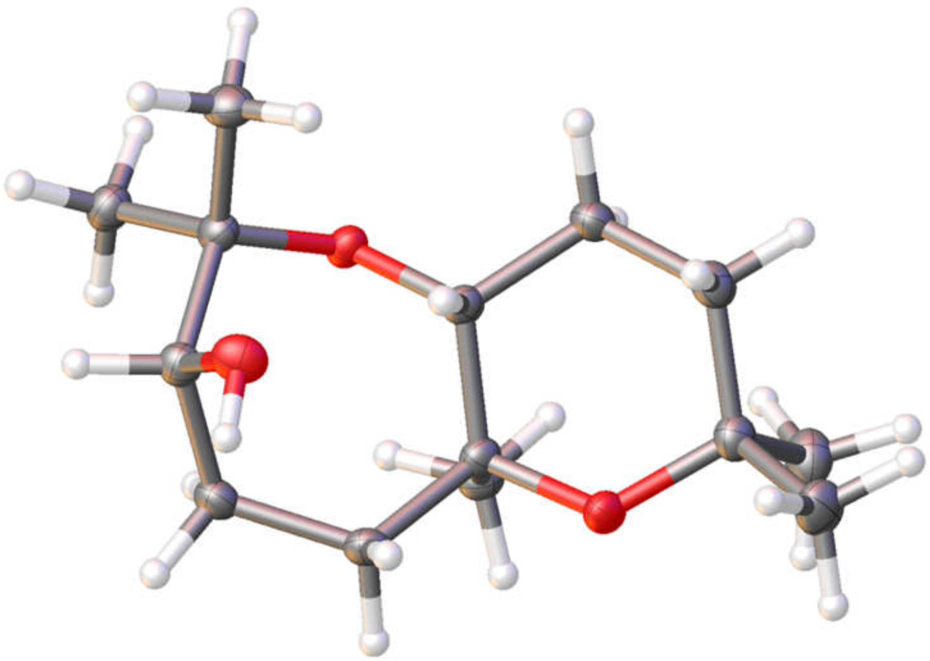

Fig. S87 Crystal structure for product *trans,trans* (AA)-**33**.

Table S22 Crystallographic data of *trans,trans* (AA)-**33** (CCDC 1999318)

Formula	$C_{14}H_{26}O_3$	V (Å^3)	1347.88(5)
Space Group	$P2_1/n$	Z	4
a (Å)	5.84981(12)	ρ_{calc} (g/cm^3)	1.194
b (Å)	15.5147(4)	F_{000}	536.0
c (Å)	14.8525(3)	T (K)	150
α (°)	90	Reflection collected	14706
β (°)	90.681(2)	R_1 ($I > 2.00\sigma(I)$)	0.0325
γ (°)	90	wR_2 (all data)	0.0812
